# Supplementary material for: Epidemiology of community-acquired pneumonia among hospitalised children in Indonesia: a multicentre, prospective study
Source: BMJ Open. 2022 Jun 21;12(6):e057957. doi: 10.1136/bmjopen-2021-057957 (PMC9214401; doi:10.1136/bmjopen-2021-057957)
Supplement: Supplementary data [file bmjopen-2021-057957supp001.pdf]

SUPPLEMENTARY MATERIALS.

Supplementary Table 1. Microbiological, Molecular and Serologic Methods

No.	Assays	Procedures
1.	Gram stain	Gram-stained smears were obtained from the most purulent portion of each induced sputum specimen. The good quality specimen was defined as <10 squamous epithelium per low-power field (magnification, 100×) [1]. The procedure of the Gram stain required four basic steps that include applied a primary stain (crystal violet) to a heat-fixed smear, followed by the addition of a mordant (Gram's Iodine), rapid decolorization with alcohol, acetone, or a mixture of alcohol and acetone and lastly, counterstained with safranin [2]. The Gram-stained smears interpreted as follows: Gram-positive lancet-shaped diplococci (GPDC) suggest *Streptococcus pneumoniae*; Gram-positive diplococci (GPDC) or cocci in chains suggest *Streptococcus pyogenes*; Gram-positive cocci in clusters (GPC-cluster) suggest *Staphylococcus aureus*; Gram-negative coccobacilli (GNCB) suggest *Hemophilus influenzae*, *Bordetella pertussis* or *Acinetobacter baumannii*; Gram-negative diplococci (GNDC) suggest *Moraxella catarrhalis*; large Gram-negative rods (GNR-large) suggest *Klebsiella pneumoniae* or *Escherichia coli*; and small Gram-negative rods (GNR-small) suggest *Pseudomonas aeruginosa* [3].
2.	Induced Sputum Culture	The most purulent portion of induced sputum was inoculated onto sheep blood, chocolate, and MacConkey agars, streaked out using a standard 4-quadrant streaking method, and incubated at 35°C for 48 hours. Cultures were examined at 24 hours and 48 hours, and predominant bacteria were identified and quantified according to the farthest quadrant with visible colonies (first quadrant, scanty; second quadrant, 1+; third quadrant, 2+; fourth quadrant, 3+) [4]. Then, the predominant bacteria isolates were inoculated into the appropriate VITEK identification strip using the VITEK® 2 COMPACT (BioMérieux, Germany). Briefly, a bacterial suspension was adjusted to a McFarland standard of 0.50 in a solution of 0.45 % sodium chloride using DensiLameter. The time between preparation of the solution and filling of the card was always less than 1 h. Analysis was done using the identification card and automatically read every 15 min. Bacteria identification and antibiotic susceptibility testing results were analyzed using the VITEK 2 software according to the manufacturer's instructions [5].
3.	Blood Culture	Up to 2 mL of blood samples (2 bottle sets) were collected and sent to the site laboratory with standardized procedures. Blood cultures were incubated for at least 5 days, unless positive, using automated systems (BacT/ALERT in Tangerang Hospital; BACTEC at other sites) [6]. Organisms were identified according to standard microbiological methods as described in induced sputum culture section. The following organisms were considered to be contaminants when identified in blood cultures: Coagulase-negative *staphylococci*, *Micrococcus* spp., *Propionibacterium* spp., Alpha-hemolytic streptococci (except

No.	Assays	Procedures
		pneumococcus, *Streptococcus anginosus*, and *Streptococcus mitis*), *Enterococcus* spp., *Corynebacterium* spp. (diphtheroids), *Bacillus* spp. (except *Bacillus anthracis*), *Pseudomonas* spp. (except *Pseudomonas aeruginosa*), *Stomatococcus, Aeroccocus, Neiserria subflava, Veillonella* spp., other environmental non-fermenting Gram negative rods, and *Candida* spp [7].
4.	Viral RNA Extraction	Viral RNA was extracted from viral transport media (VTM) containing respiratory swab as well as sputum, using the QIAamp Viral RNA Mini Kit (Qiagen, Hilden, Germany) according to the manufacturer's protocol. Briefly, 140 µl of VTM or sputum coat was lysed in 560 of carrier RNA-containing AVL buffer, followed by the binding of viral RNA to the QIAamp membrane. Contaminants were removed from viral RNA in two separate washing steps using two different wash buffers, AW1 and AW2. Viral RNA was eluted in 60 µl of AVE buffer and kept in -80° C if not directly used [8,9].
5.	Bacterial DNA Extraction	Bacterial DNA was extracted from viral transport media (VTM) containing respiratory swab as well as sputum, using the QIAamp® DNA Mini Kit (Qiagen, Hilden, Germany) according to the manufacturer's protocol. Briefly, 20 µl of QIAGEN Protease and 200 µl of VTM or sputum coat was lysed in 200 of AL buffer, followed binding of DNA to the QIAamp membrane. Contaminants were removed from DNA in two separate washing steps using two different wash buffers, AW1 and AW2. Bacterial DNA was eluted in 200 µl of AE buffer and kept in -80° C if not directly used
6.	qPCR for Respiratory Viruses	The realtime PCR for respiratory virus detection was done followed the protocol of Beld et al., 2004 and Jansen et al., 2011. Positive control is a synthetic plasmid carrying the nucleotide sequence of the detection target. Primers, probes, and positive controls were synthesized and purified by an outside vendor (Integrated DNA Technologies, Iowa, US). Realtime PCR was done using the TaqManTM Fast Virus 1-Step Master Mix (Thermo Fisher Scientific; Cat#: 4444432) in an Applied Biosystems 7500 Fast Realtime PCR System (Thermo Fisher Scientific, MA, US). The reaction mixture composition was 1X TaqManTM Fast Virus 1-Step Master Mix, 0.5 µM of each primer, 0.25 µM probe, and 4 µl RNA, in a total 20 µl volume. The cycle condition was 50° reverse transcription for 5 minutes, 95° C initial denaturation for 20 seconds, followed by 45 cycles of denaturation (95° C, 3 seconds) and annealing/elongation (55° C, 30 seconds). Realtime PCR works correctly when the positive control demonstrates the amplification curve and the template-free (negative) control demonstrates no amplification curve (no Ct values) [8,9].
7.	qPCR for Respiratory Bacteria	In real-time PCR (qPCR) a portion of bacterial DNA genome specific to the pathogen(s) of interest is amplified using a specific pair of primers and probes for each bacteria, that were selected from the available literature [10–14]. A detector (TaqMan® probe) is used in the reaction. Mastermix is prepared in a 1.5-ml tube for total reaction. qPCR assays were carried out in a total volume of 20 µL, comprising 10 µL of TaqMan® Fast Universal PCR Master Mix, 1.4 µL of nuclease-free water (Promega), 3.6 µL of oligonucleotide mixtures, and 4 µL of

No.	Assays	Procedures
		DNA extract. The cycle condition was 95° C initial denaturation for 20 seconds, followed by 45 cycles of denaturation (95° C, 3 seconds) and annealing/elongation (58° C, 30 seconds). Realtime PCR works correctly when the positive control demonstrates the amplification curve and the template-free (negative) control demonstrates no amplification curve (no Ct values)
8.	Serology Test	Assays were obtained from SERION ELISA classic kit (Institut Virion/Serion Laboratories, Germany) and used according to the insert of SERION kit. SERION ELISA classic is a qualitative and quantitative immunoassay for detecting human antibodies in serum or plasma with their corresponding antigen. The indirect enzyme immunosorbent assay in this kit was coated with specific antigens of the pathogen of interest. Patient sera are diluted in a rheumatoid factor and then diluted in Sample Diluent (containing phosphate with tween 20 and Bromphenol blue) and incubated in the coated microwells to bind serum antibody to the solid-phase antigen. The microwells are then washed to remove unreacted serum proteins, and enzyme conjugate (anti-human IgA, IgG, or IgM APC_Alkaline phosphatase) is added to label the bound antibody. After further incubation, the microwells are washed to remove unbound APC Conjugate. The pNPP (para-nitrophenyl phosphate) substrate is then added to quantitate the Conjugate-bound p-nitrophenyl phosphate portion. The colorless substrate p-nitrophenyl phosphate is then converted into the colored product p-nitrophenol. The signal intensity of this reaction product is proportional to the concentration of the analyte in the serum antibody. This timed reaction is interrupted with a Stop Solution (sodium hydroxide). Color intensity (Absorbance) is measured at a wavelength of 405nm on a microtiter plate reader or spectrophotometer within 15 minutes of adding the stop solution. Antibody activities are calculated by the SERION evaluation software [15].

Footnote References:

1. Murdoch DR, Morpeth SC, Hammitt LL, et al. Microscopic Analysis and Quality Assessment of Induced Sputum From Children With Pneumonia in the PERCH Study. *Clin Infect Dis an Off Publ Infect Dis Soc Am*. 2017;64(suppl_3):S271-S279. doi:10.1093/cid/cix083
2. Smith AC, Hussey MA. Gram stain protocols. *Am Soc Microbiol*. 2005;1:14.
3. Mardian Y, Menur Naysilla A, Lokida D, et al. Approach to Identifying Causative Pathogens of Community-Acquired Pneumonia in Children Using Culture, Molecular, and Serology Tests. *Front Pediatr*. 2021;9:498. doi:10.3389/fped.2021.629318
4. Murdoch DR, Morpeth SC, Hammitt LL, et al. The Diagnostic Utility of Induced Sputum Microscopy and Culture in Childhood Pneumonia. *Clin Infect Dis an Off Publ Infect Dis Soc Am*. 2017;64(suppl_3):S280-S288. doi:10.1093/cid/cix090
5. Książczyk M, Kuczkowski M, Dudek B, et al. Application of Routine Diagnostic Procedure, VITEK 2 Compact, MALDI-TOF MS, and PCR Assays in Identification Procedure of Bacterial Strain with Ambiguous Phenotype. *Curr Microbiol*. 2016;72(5):570-582. doi:10.1007/s00284-016-0993-0
6. Kirn TJ, Weinstein MP. Update on blood cultures: how to obtain, process, report, and interpret. *Clin Microbiol Infect Off Publ Eur Soc Clin Microbiol Infect Dis*. 2013;19(6):513-520. doi:10.1111/1469-0691.12180
7. O'Brien KL, Baggett HC, Brooks WA, et al. Causes of severe pneumonia requiring hospital admission in children without HIV infection from Africa and Asia: the PERCH multi-country case-control study. *Lancet*. 2019;394(10200):757-779. doi:10.1016/S0140-6736(19)30721-4
8. Beld M, Minnaar R, Weel J, et al. Highly sensitive assay for detection of enterovirus in clinical specimens by reverse transcription-PCR with an armored RNA internal control. *J Clin Microbiol*. 2004;42(7):3059-3064. doi:10.1128/JCM.42.7.3059-3064.2004
9. Jansen RR, Schinkel J, Koekkoek S, et al. Development and evaluation of a four-tube real time multiplex PCR assay covering fourteen respiratory viruses, and comparison to its corresponding single target counterparts. *J Clin Virol Off Publ Pan Am Soc Clin Virol*. 2011;51(3):179-185. doi:10.1016/j.jcv.2011.04.010

10. Reischl U, Lehn N, Sanden GN, Loeffelholz MJ. Real-time PCR assay targeting IS481 of Bordetella pertussis and molecular basis for detecting Bordetella holmesii. *J Clin Microbiol*. 2001;39(5):1963-1966. doi:10.1128/JCM.39.5.1963-1966.2001
11. Pitcher D, Chalker VJ, Sheppard C, George RC, Harrison TG. Real-time detection of Mycoplasma pneumoniae in respiratory samples with an internal processing control. *J Med Microbiol*. 2006;55(Pt 2):149-155. doi:10.1099/jmm.0.46281-0
12. Greiner O, Day PJR, Altwegg M, Nadal D. Quantitative detection of Moraxella catarrhalis in nasopharyngeal secretions by real-time PCR. *J Clin Microbiol*. 2003;41(4):1386-1390. doi:10.1128/JCM.41.4.1386-1390.2003
13. Meyler KL, Meehan M, Bennett D, Cunney R, Cafferkey M. Development of a diagnostic real-time polymerase chain reaction assay for the detection of invasive Haemophilus influenzae in clinical samples. *Diagn Microbiol Infect Dis*. 2012;74(4):356-362. doi:10.1016/j.diagmicrobio.2012.08.018
14. Gadsby NJ, McHugh MP, Russell CD, et al. Development of two real-time multiplex PCR assays for the detection and quantification of eight key bacterial pathogens in lower respiratory tract infections. *Clin Microbiol Infect Off Publ Eur Soc Clin Microbiol Infect Dis*. 2015;21(8):788.e1-788.e13. doi:10.1016/j.cmi.2015.05.004
15. Gasem MH, Kosasih H, Tjitra E, et al. An observational prospective cohort study of the epidemiology of hospitalized patients with acute febrile illness in Indonesia. *PLoS Negl Trop Dis*. 2020;14(1):e0007927. doi:10.1371/journal.pntd.0007927

Supplementary Table 2. Antibiotic regimens administered prior to blood culture

Antibiotic Regimen, (Dose)	All sites (N=188), Administered Dose(s) prior to blood culture, N (%)	Semarang (N=47) Administered Dose(s) prior to blood culture, N (%)	Yogyakarta (N=52) Administered Dose(s) prior to blood culture, N (%)	Tangerang (N=89) Administered Dose(s) prior to blood culture, N (%)
Ampicillin (50 mg/kg IV q6hr) + Gentamicin (2 – 7.5 mg/kg IV q24hr)	65 (34.6)<br>1x: 45 (24.0)<br>2x: 20 (10.6)	25 (53.2)<br>1x: 20 (42.6)<br>2x: 5 (10.6)	40 (76.9)<br>1x: 25 (48.1)<br>2x: 15 (28.8)	0 (0)
Cefotaxime (50 – 100 mg/kg IV q6hr)	32 (17.0)<br>All received 1 dose	0 (0)	0 (0)	32 (36.0)<br>All received 1 dose
Ceftriaxone (50 mg/kg IV q12hr)	27 (14.4)<br>All received 1 dose	0 (0)	0 (0)	27 (30.3)<br>All received 1 dose
Ampicillin (50 mg/kg IV q6hr)	14 (7.4)<br>1x: 10 (5.3)<br>2x: 4 (2.1)	5 (10.6)<br>All received 1 dose	9 (17.3)<br>1x: 5 (9.6)<br>2x: 4 (7.7)	0 (0)
Gentamicin (2 – 7.5 mg/kg IV q24hr)	3 (1.6)<br>1x: 2 (1.1)<br>2x: 1 (0.5)	3 (6.4)<br>1x: 2 (4.3)<br>2x: 1 (2.1)	0 (0)	0 (0)
Ceftazidime (50 – 100 mg/kg IV q8hr)	3 (1.6)<br>All received 1 dose	0 (0)	0 (0)	3 (3.4)<br>All received 1 dose
Cefamandole (50 – 100 mg/kg IV q12hr)	2 (1.1)<br>1x: 1 (0.5)<br>2x: 1 (0.5)	2 (4.3)<br>1x: 1 (2.1)<br>2x: 1 (2.1)	0 (0)	0 (0)
Ceftriaxone (50 mg/kg IV q12hr) + Gentamicin (2 – 7.5 mg/kg IV q24hr)	2 (1.1)<br>All received 1 dose	2 (4.3)<br>All received 1 dose	0 (0)	0 (0)
Amikacin (15 mg/kg IV q8hr) + Cefotaxime (50 – 100 mg/kg IV q6hr)	1 (0.5)<br>All received 1 dose	1 (2.1)<br>All received 1 dose	0 (0)	0 (0)
Amoxicillin syrup (40 mg/kg PO q12hr)	1 (0.5)<br>All received 1 dose	1 (2.1)<br>All received 1 dose	0 (0)	0 (0)

IV = intravenous; PO = peroral; qXhr = given at X hour intervals.

Supplementary Table 3. Pathogen distribution by WHO severity classification status and mortality.

Pathogens	WHO Classification System		*p-value*	Mortality Outcome		*p-value*
	Severe (N=89)	Non-severe (N=99)		Died (N=19)	Alive (N=169)	
**Causative Pathogen**						
H. influenzae non-type b	31 (34.8%)	42 (42.4%)	0.286	8 (42.1%)	65 (38.5%)	0.757
RSV	25 (28.1%)	26 (26.3%)	0.778	2 (10.5%)	49 (29.0%)	0.086
K. pneumoniae	15 (16.9%)	28 (28.3%)	0.062	6 (31.6%)	37 (21.9%)	0.388
S. pneumoniae	19 (21.3%)	10 (10.1%)	0.033	1 (5.2%)	28 (16.6%)	0.317
Influenza virus	9 (10.1%)	16 (16.2%)	0.223	3 (15.8%)	22 (13.0%)	0.723
S. aureus	8 (9.0%)	12 (12.1%)	0.487	0 (0.0%)	20 (11.8%)	0.230
PIV	8 (9.0%)	9 (9.1%)	0.981	1 (5.3%)	16 (9.5%)	1.000
hMPV	6 (6.7%)	5 (5.1%)	0.622	1 (5.3%)	10 (5.9%)	1.000
Rhinovirus	7 (7.9%)	3 (3.0%)	0.196	1 (5.3%)	9 (5.3%)	1.000
B. pertussis	4 (4.5%)	3 (3.0%)	0.709	2 (10.5%)	5 (3.0%)	0.150
**Infection Type**						
Bacterial pathogen	17 (19.1%)	31 (31.3%)	0.055	7 (36.8%)	41 (24.3%)	0.268
Viral pathogen	16 (18.0%)	15 (15.2%)	0.602	2 (10.5%)	29 (17.2%)	0.744
Mixed pathogen	38 (42.7%)	38 (38.4%)	0.547	5 (26.3%)	71 (42.0%)	0.186
Unknown pathogen	18 (20.2%)	15 (15.2%)	0.361	5 (26.3%)	28 (16.6%)	0.337

Differences in categorical variables were compared using Pearson χ2 or Fisher's exact test when the expected values in any of the contingency table cells were below 5.

Supplementary Table 4. Summary of fatal cases.

Case, Site, Gender (Age, mo)	Medical History	Signs and Symptoms (SS), Vital Signs (VS), Laboratory parameter (Lab) at admission	CXR	Causative Pathogen	ABX during Hospitalization	Hospitalization status	Cause of Death
#01, SMG, Male (4)	Recurrent pneumonia, congenital heart disease, severe malnutrition	• **SS:** Cough, fever, dyspnea, chest indrawing, intercostal retraction, rhonchi<br>• **VS:** 38°C, RR 44x/min, SpO_2 97%<br>• **Lab:** Hb 9.6 g/dL, WBC 24.1 $\times 10^9$/L, PLT 350 $\times 10^9$/L, NLR 4.63, CRP 25.70 mg/L, PCT 2.41 ng/mL	Alveolar infiltrate	Rhinovirus, *H. influenzae non-type b*	Ampicillin, Gentamicin, Ceftriaxon, Cefoperazone Sulbactam	On mechanical ventilator<br>ICU admission (25 days)<br>Died on Day-26	Cardiopulmonary failure<br>Sepsis
#02, SMG, Female (23)	Recurrent pneumonia, congenital heart disease, incomplete NIP (DPT-Hib), malnutrition, developmental delay	• **SS:** Cough, fever, dyspnea, chest indrawing, intercostal retraction, rhonchi<br>• **VS:** 37.5°C, RR 56x/min, SpO_2 95%<br>• **Lab:** Hb 10.6 g/dL, WBC 14.1 $\times 10^9$/L, PLT 405 $\times 10^9$/L, NLR 9.63, CRP 14.90 mg/L, PCT 0.37 ng/mL	Alveolar and interstitial infiltrates	Influenza A (H1N1)	Ampicillin, Gentamicin, Metronidazole, Ceftriaxon, Meropenem	On mechanical ventilator<br>ICU admission (9 days)<br>Died on Day 21	Cardiopulmonary failure
#03, SMG, Female (11)	Low birth weight, congenital heart disease, incomplete NIP (Measles), severe malnutrition, developmental delay	• **SS:** Cough, fever, dyspnea, diarrhea, nasal flaring, chest indrawing, intercostal retraction, rhonchi<br>• **VS:** 38.3°C, RR 45x/min, SpO_2 96%<br>• **Lab:** Hb 8.1 g/dL, WBC 15.9 $\times 10^9$/L, PLT 677 $\times 10^9$/L, NLR 1.87	Alveolar and interstitial infiltrates	Influenza A (H3N2), *B. pertussis, H. influenzae non-type b*, *K. pneumoniae*	Ampicillin, Gentamicin, Azithromycin	On nasal cannula<br>Died on day 19	Cardiopulmonary failure
#04, SMG, Male (45)	Recurrent pneumonia, frontometaphyseal dysplasia syndrome,	• **SS:** Cough, fever, dyspnea, nasal flaring, intercostal retraction, rhonchi, wheezing<br>• **VS:** 36.7°C, RR 40x/min, SpO_2 99%	Alveolar infiltrate	Unknown	Ampicillin, Gentamicin	On Nasal cannula<br>Died on day 2	Respiratory failure

Case, Site, Gender (Age, mo)	Medical History	Signs and Symptoms (SS), Vital Signs (VS), Laboratory parameter (Lab) at admission	CXR	Causative Pathogen	ABX during Hospitalization	Hospitalization status	Cause of Death
	epilepsy, developmental delay	• **Lab:** Hb 13.7 g/dL, WBC 11.3 $\times10^9$/L, PLT 277 $\times10^9$/L, NLR 0.98, CRP 0.10 mg/L, PCT 0.05 ng/mL					
#05, SMG, Male (5)	Premature birth, low birth weight, recurrent pneumonia, congenital heart disease, incomplete NIP (DPT-Hib)	• **SS:** Cough, dyspnea, nasal flaring, chest indrawing, intercostal retraction,<br>• **VS:** 36.8^{o}C, RR 30x/min, SpO_2 98%<br>• **Lab:** Hb 10.9 g/dL, WBC 12.4 $\times10^9$/L, PLT 396 $\times10^9$/L, CRP 0.80 mg/L, PCT 128 ng/mL	Alveolar infiltrate	*K. pneumoniae*	Ampicillin, Gentamicin	On Simple mask<br>ICU admission (1 day)<br>Died on day 6	Cardiopulmonary failure
#06, SMG, Female (3)	Recurrent pneumonia, incomplete NIP (DPT-Hib), malnutrition	• **SS:** Cough, dyspnea, chest indrawing, intercostal retraction, rhonchi<br>• **VS:** 36.7^{o}C, RR 42x/min, SpO_2 99%<br>• **Lab:** Hb 8.2 g/dL, WBC 16 $\times10^9$/L, PLT 499 $\times10^9$/L, ANC 6.7, NLR 0.76, CRP 13.10 mg/L, PCT 0.28 ng/mL	Alveolar infiltrate	Unknown	Ampicillin, Gentamicin, Vancomycin, Metronidazol, Meropenem	On mechanical ventilator<br>ICU admission (7 days)<br>Died on day 18	Septic shock, respiratory failure
#07, YGY, Female (10)	Congenital heart disease, incomplete NIP (DPT-Hib, and Measles), severe malnutrition, developmental delay	• **SS:** Cough, fever, dyspnea, head bobbing, chest indrawing, intercostal retraction, rhonchi<br>• **VS:** 39.0 ^{o}C, RR 64x/min, SpO_2 96%<br>• **Lab:** Hb 10.1 g/dL, WBC 12.1 $\times10^9$/L, PLT 415 $\times10^9$/L, ANC 6.0, NLR 1.15, CRP 4.90 mg/L, PCT 0.11 ng/mL	Alveolar infiltrate	hMPV, RSV A	Ampicillin, Gentamicin, Ceftriaxone, Cotrimoxazole	On mechanical ventilator/ ICU admission (13 days)<br>Died on day 17	Sepsis, Pulmonary crisis due to pulmonary hypertension
#08, YGY, Female (3)	Low birth weight, congenital heart disease, incomplete NIP (DPT-Hib), severe malnutrition	• **SS:** Cough, fever, dyspnea, chest indrawing, intercostal retraction, rhonchi<br>• **VS:** 37.2 ^{o}C, RR 49x/min, SpO_2 56%<br>• **Lab:** Hb 9.7 g/dL, WBC 11.3 $\times10^9$/L, PLT 115 $\times10^9$/L, ANC 7.0, NLR 1.92	Alveolar and interstitial infiltrates	Unknown	Ampicillin, Ceftriaxone	On nasal cannula<br>Hospital discharge on day 10<br>Died on day 29 (outside hospitalization)	Acute Respiratory Distress Syndrome
#09, YGY, Female (5)	Congenital heart disease, incomplete NIP (DPT-Hib), severe malnutrition	• **SS:** Cough, dyspnea, inability to drink, nasal flaring, chest indrawing, intercostal retraction, rhonchi<br>• **VS:** 37.0 ^{o}C, RR 60x/min, SpO_2 96%<br>• **Lab:** Hb 10.3 g/dL, WBC 26.9 $\times10^9$/L, PLT 788 $\times10^9$/L, ANC 18.5, NLR 2.97	Alveolar and interstitial infiltrates	*H. influenzae non-type b, K. pneumoniae*	Ampicillin, Gentamicin	On nasal cannula<br>Died on day 15	Aspiration, mucous hypersecretion
#10, YGY, Male (6)	Recurrent pneumonia, congenital heart disease, tuberculosis, incomplete NIP (DPT-Hib)	• **SS:** Cough, fever, dyspnea, nasal flaring, chest indrawing, intercostal retraction, rhonchi, wheezing<br>• **VS:** 37.3 ^{o}C, RR 50x/min, SpO_2 89%<br>• **Lab:** Hb 11.6 g/dL, WBC 13.3 $\times10^9$/L, PLT 189 $\times10^9$/L, ANC 3.7, NLR 0.48, CRP 4.90 mg/L, PCT 0.08 ng/mL	Alveolar and interstitial infiltrates, pleural effusion	*K. pneumoniae*	Ampicillin, Gentamicin, Ceftriaxone	On non-rebreather mask<br>Died on day 4	Septic shock
#11, TRG, Female (5)	Premature birth, developmental delay	• **SS:** Cough, fever, dyspnea, nasal flaring, rhonchi, wheezing<br>• **VS:** 37.5 ^{o}C, RR 48x/min, SpO_2 31%<br>• **Lab:** Hb 8.5 g/dL, WBC 12.1 $\times10^9$/L, PLT 208 $\times10^9$/L, ANC 8.6, NLR 3.23, CRP 0.91 mg/L, PCT 0.74 ng/mL	Alveolar infiltrate	*A. baumannii* (MDR)	Cefotaxime	On Nasal cannula<br>Hospital discharge on day 7<br>Died on day 17 (outside hospitalization)	Unknown death
#12, TRG, Female (2)	Incomplete NIP (DPT-Hib)	• **SS:** Cough, fever, dyspnea, diarrhea, skin rash, intercostal retraction, rhonchi, wheezing<br>• **VS:** 37.6 ^{o}C, RR 63x/min, SpO_2 93%<br>• **Lab:** Hb 10.5 g/dL, WBC 13.6 $\times10^9$/L, PLT 289 $\times10^9$/L, ANC 10.2, NLR 3.95, CRP 175.30 mg/L, PCT 0.7 ng/mL	Alveolar and interstitial infiltrates	Unknown	Ceftriaxone, Ceftazidime, Azithromycin	On Nasal cannula<br>Died on day 8	Sepsis
#13, TRG, Female (2)	Incomplete NIP (DPT-Hib)	• **SS:** Cough, fever, dyspnea, nasal flaring, chest indrawing, intercostal retraction, rhonchi<br>• **VS:** 36 ^{o}C, RR 45x/min, SpO_2 96%<br>• **Lab:** Hb 7.8 g/dL, WBC 21.2 $\times10^9$/L, PLT 563 $\times10^9$/L, ANC 16.5, NLR 3.9, CRP 280.30 mg/L, PCT 0.09 ng/mL	Alveolar and interstitial infiltrates, pleural effusion	Influenza B, *S. mitis* (MDR)	Ceftazidime	On non-rebreather mask ICU admission (3 days)<br>Died on day 3	Respiratory Failure

Case, Site, Gender (Age, mo)	Medical History	Signs and Symptoms (SS), Vital Signs (VS), Laboratory parameter (Lab) at admission	CXR	Causative Pathogen	ABX during Hospitalization	Hospitalization status	Cause of Death
#14, TRG, Female (2)	Congenital heart disease, incomplete NIP (DPT-Hib), severe malnutrition	• **SS:** Cough, fever, dyspnea, nasal flaring, chest indrawing, intercostal retraction, rhonchi, wheezing<br>• **VS:** 37 ^{o}C, RR 60x/min, SpO_2 76%<br>• **Lab:** Hb 9.5 g/dL, WBC 17.2 $\times10^9$/L, PLT 296 $\times10^9$/L, ANC 8.8, NLR 1.42, CRP 0.70 mg/L, PCT 0.02 ng/mL	Interstitial infiltrate	Unknown	Cefotaxime	On Simple mask<br>Died on day 2	Respiratory Failure
#15, TRG, Male (9)	Incomplete NIP (Measles)	• **SS:** Cough, fever, dyspnea, nasal flaring, chest indrawing, intercostal retraction, rhonchi<br>• **VS:** 37 ^{o}C, RR 30x/min, SpO_2 89%<br>• **Lab:** Hb 6.4 g/dL, WBC 25.7 $\times10^9$/L, PLT 801 $\times10^9$/L, ANC 18.5, NLR 3.43, CRP 33.35 mg/L, PCT 0.34 ng/mL	Interstitial infiltrate	*H. influenzae non-type b*	Cefotaxime, Ceftriaxone, Meropenem	On mechanical ventilator<br>ICU admission (8 days)<br>Died on day 12	Meningoencephalitis, Respiratory Failure
#16, TRG, Female (4)	Premature birth, low birth weight, congenital heart disease, incomplete NIP (DPT-Hib)	• **SS:** Cough, fever, dyspnea, diarrhea, chest indrawing, intercostal retraction, rhonchi<br>• **VS:** 38 ^{o}C, RR 32x/min, SpO_2 85%<br>• **Lab:** Hb 9.2 g/dL, WBC 16.8 $\times10^9$/L, PLT 224 $\times10^9$/L, ANC 9.4, NLR 2.24, CRP 2.46 mg/L, PCT 2.24 ng/mL	Alveolar and interstitial infiltrates,	*H. influenzae non-type b*, *K. pneumoniae*	Cefotaxime, Gentamicin, Ceftriaxone	On nasal cannula<br>Died on day 11	Unknown death
#17, TRG, Female (20)	Developmental delay, incomplete NIP (DPT-Hib)	• **SS:** Cough, fever, dyspnea, chest indrawing, intercostal retraction, rhonchi<br>• **VS:** 36.3^{o}C, RR 40x/min, SpO_2 75%<br>• **Lab:** Hb 7.0 g/dL, WBC 15.2 $\times10^9$/L, PLT 668 $\times10^9$/L, ANC 9.7, NLR 2.13, CRP 55.10 mg/L	Alveolar and interstitial infiltrates, pleural effusion	*H. influenzae non-type b*, *K. pneumoniae*	Cefotaxime, Gentamicin, Ceftriaxone	On mechanical ventilator<br>ICU admission (3 days)<br>Died on day 8	Septic shock, Cardiopulmonary failure
#18, TRG, Male (4)	Low birth weight, developmental delay, recurrent pneumonia, incomplete NIP (DPT-Hib), severe malnutrition	• **SS:** Cough, fever, dyspnea, nasal flaring, chest indrawing, intercostal retraction, rhonchi<br>• **VS:** 36.7 ^{o}C, RR 30x/min, SpO_2 92%<br>• **Lab:** Hb 11.6 g/dL, WBC 20.5 $\times10^9$/L, PLT 433 $\times10^9$/L, ANC 11.9, NLR 2.52, CRP 16.80 mg/L, PCT 20.1 ng/mL	Alveolar and interstitial infiltrates,	PIV 3, *H. influenzae non-type b*, *S. pneumoniae*	Ceftazidime	On non-rebreather mask<br>Died on day 3	Respiratory failure
#19, TRG, Male (15)	Incomplete NIP (DPT-Hib and Measles)	• **SS:** Cough, fever, dyspnea, rhonchi<br>• **VS:** 37.8 ^{o}C, RR 52x/min, SpO_2 80%<br>• **Lab:** Hb 9.4 g/dL, WBC 23.6 $\times10^9$/L, PLT 786 $\times10^9$/L, CRP 3.30 mg/L, PCT 0.07 ng/mL	Interstitial infiltrate	RSV B, *B. pertussis, H. influenzae non-type b*	Cefotaxime	On nasal cannula<br>Hospital discharged on day 5<br>Died on day 20 (outside hospitalization)	Unknown death

Abbreviation. SMG: Semarang site; YGY: Yogyakarta site; TGR: Tangerang site; NIP: mandatory National Immunization Program; DPT-Hib: a combined vaccine of adsorbed diphtheria, tetanus toxoids, acellular pertussis and of *Haemophilus influenzae* type b conjugate vaccines; CXR: chest X-ray; ABX: Antibiotics; RSV: Respiratory Syncytial Virus; hMPV: Human Metapneumovirus; PIV: Parainfluenza Virus; MDR: Multiple drug resistance.

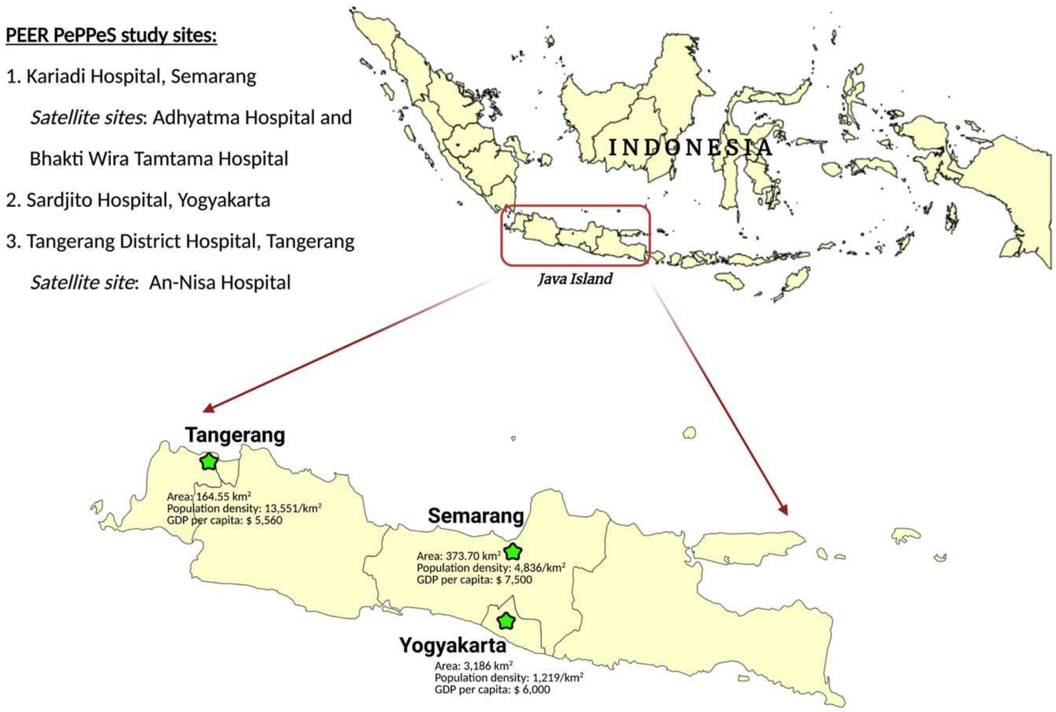

Supplementary Figure 1. PEER-PePPeS Study sites

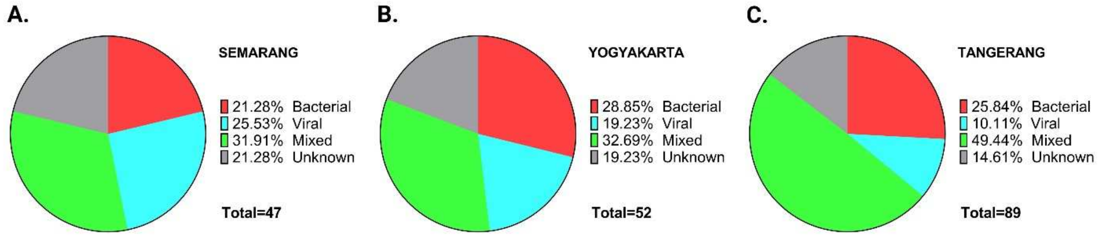

Supplementary Figure 2. Proportion of Identified Pathogen in each Sites. (A) Semarang, (B) Yogyakarta, and (C) Tangerang

BMJ Publishing Group Limited (BMJ) disclaims all liability and responsibility arising from any reliance placed on this supplemental material which has been supplied by the author(s)

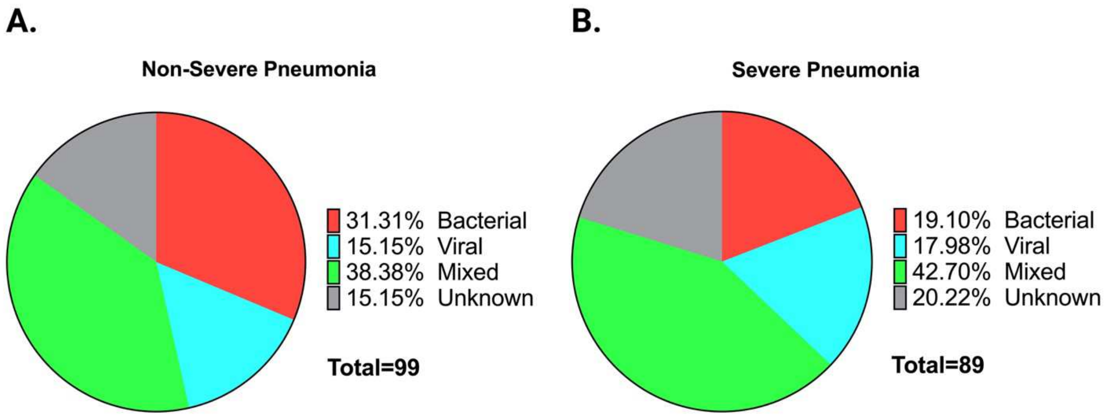

Supplementary Figure 3. Proportion of Identified Pathogen between WHO Severity Status. (A) Non-severe Pneumonia, (B) Severe Pneumonia.
