# Supplementary material for: Epidemiology of community-acquired pneumonia among hospitalised children in Indonesia: a multicentre, prospective study
Source: BMJ Open. 2022 Jun 21;12(6):e057957. doi: 10.1136/bmjopen-2021-057957 (PMC9214401; doi:10.1136/bmjopen-2021-057957)
Supplement: Author's manuscript [file bmjopen-2021-057957.draft_revisions.pdf]

BMJ Open is committed to open peer review. As part of this commitment we make the peer review history of every article we publish publicly available.

When an article is published we post the peer reviewers' comments and the authors' responses online. We also post the versions of the paper that were used during peer review. These are the versions that the peer review comments apply to.

The versions of the paper that follow are the versions that were submitted during the peer review process. They are not the versions of record or the final published versions. They should not be cited or distributed as the published version of this manuscript.

BMJ Open is an open access journal and the full, final, typeset and author-corrected version of record of the manuscript is available on our site with no access controls, subscription charges or pay-per-view fees (http://bmjopen.bmj.com).

If you have any questions on BMJ Open's open peer review process please email info.bmjopen@bmj.com

Epidemiology of community-acquired pneumonia among hospitalized children in Indonesia: a multicenter, prospective study

Journal:	*BMJ Open*
Manuscript ID	bmjopen-2021-057957
Article Type:	Original research
Date Submitted by the Author:	18-Oct-2021

Complete List of Authors:	Lokida, Dewi; Tangerang District General Hospital<br>Farida, Helmia; Rumah Sakit Umum Pusat Dr Kariadi<br>Triasih, Rina; Rumah Sakit Umum Pusat Dr Sardjito<br>Mardian, Yan; Indonesia Research Partnership on Infectious Disease<br>Kosasih, Herman; Indonesia Research Partnership on Infectious Disease<br>Naysilla, Adhella Menur; Indonesia Research Partnership on Infectious Disease<br>Budiman, Arif; Tangerang District General Hospital<br>Hayuningsih, Chakrawati; Tangerang District General Hospital<br>Anam, Moh. Syarofil; Rumah Sakit Umum Pusat Dr Kariadi<br>Wastoro, Dwi; Rumah Sakit Umum Pusat Dr Kariadi<br>Mujahidah, Mujahidah; Rumah Sakit Umum Pusat Dr Sardjito<br>Dipayana, Setya; Rumah Sakit Umum Pusat Dr Kariadi<br>Setyati, Amalia; Rumah Sakit Umum Pusat Dr Sardjito<br>Aman, Abu; Rumah Sakit Umum Pusat Dr Sardjito<br>Lukman, Nurhayati; Indonesia Research Partnership on Infectious Disease<br>Karyana, Muhammad; National Institute of Health Research and Development, Ministry of Health, Republic of Indonesia<br>Kline, Ahnika; National Institute of Allergy and Infectious Diseases<br>Neal, Aaron; National Institute of Allergy and Infectious Diseases<br>Lau, Chuen-Yen; National Cancer Institute<br>Lane, C; National Institute of Allergy and Infectious Diseases

Keywords:	Epidemiology < TROPICAL MEDICINE, INFECTIOUS DISEASES, PAEDIATRICS

SCHOLARONE™ Manuscripts

I, the Submitting Author has the right to grant and does grant on behalf of all authors of the Work (as defined in the below author licence), an exclusive licence and/or a non-exclusive licence for contributions from authors who are: i) UK Crown employees; ii) where BMJ has agreed a CC-BY licence shall apply, and/or iii) in accordance with the terms applicable for US Federal Government officers or employees acting as part of their official duties; on a worldwide, perpetual, irrevocable, royalty-free basis to BMJ Publishing Group Ltd ("BMJ") its licensees and where the relevant Journal is co-owned by BMJ to the co-owners of the Journal, to publish the Work in this journal and any other BMJ products and to exploit all rights, as set out in our licence.

The Submitting Author accepts and understands that any supply made under these terms is made by BMJ to the Submitting Author unless you are acting as an employee on behalf of your employer or a postgraduate student of an affiliated institution which is paying any applicable article publishing charge ("APC") for Open Access articles. Where the Submitting Author wishes to make the Work available on an Open Access basis (and intends to pay the relevant APC), the terms of reuse of such Open Access shall be governed by a Creative Commons licence – details of these licences and which Creative Commons licence will apply to this Work are set out in our licence referred to above.

Other than as permitted in any relevant BMJ Author's Self Archiving Policies, I confirm this Work has not been accepted for publication elsewhere, is not being considered for publication elsewhere and does not duplicate material already published. I confirm all authors consent to publication of this Work and authorise the granting of this licence.

For peer review only

Epidemiology of community-acquired pneumonia among hospitalized children in Indonesia: a multicenter, prospective study

AUTHORS:

Dewi Lokida[1], Helmia Farida[2], Rina Triasih[3], Yan Mardian[4], Herman Kosasih[4], Adhella Menur Naysilla[4], Arif Budiman[1], Chakrawati Hayuningsih[1], Moh. Syarofil Anam[2], Dwi Wastoro[2], Mujahidah[2], Setya Dipayana[2], Amalia Setyati[3], Abu Tholib Aman[3], Nurhayati Lukman[4], Muhammad Karyana[5], Ahnika Kline[6], Aaron Neal[6], Chuen-Yen Lau[7], H. Clifford Lane[6]

AFFILIATIONS:

1. Tangerang District General Hospital
2. Rumah Sakit Umum Pusat Dr Kariadi
3. Rumah Sakit Umum Pusat Dr Sardjito
4. Indonesia Research Partnership on Infectious Disease
5. National Institute of Health Research and Development, Ministry of Health, Republic of Indonesia
6. National Institute of Allergy and Infectious Diseases
7. National Cancer Institute

ABSTRACT:

Objective:

To identify etiologies of childhood community-acquired pneumonia (CAP) based on a comprehensive diagnostic approach.

Design:

"Partnerships for Enhanced Engagement in Research - Pneumonia in Pediatrics (PEER-PePPeS)" study was an observational prospective-cohort study conducted from July 2017 through September 2019.

Setting:

Government referral teaching hospitals and satellite sites in three cities in Indonesia: Semarang, Yogyakarta, and Tangerang.

Participants:

Hospitalized children aged 2–59-months who met the criteria for pneumonia were eligible. Children were excluded if they had been hospitalized for >24 hours; had malignancy or history of malignancy;

For peer review only - http://bmjopen.bmj.com/site/about/guidelines.xhtml

a history of long-term (>2 months) steroid therapy, or conditions that might interfere with compliance with the study procedures.

Main outcome(s) measure(s):

Causative bacterial, viral, or mixed pathogen for the pneumonia was determined using microbiological, molecular, and serologic test from routinely collected specimens (blood, sputum, and nasopharyngeal swabs). We applied a previously published algorithm (PEER-PePPeS rules) to determine the causative pathogen(s).

Results:

188 subjects were enrolled. Based on our algorithm, 48 (25.5%) had a bacterial infection, 31 (16.5%) had a viral infection, 76 (40.4%) had mixed bacterial and viral infections, and 33 (17.6%) were unable to be classified. The five most common causative pathogens identified were *Haemophilus influenzae* non-type B (N=73), respiratory syncytial virus (RSV) (N=51), *Klebsiella pneumoniae* (N=43), *Streptococcus pneumoniae* (N=29), and Influenza virus (N=25). RSV and Influenza virus diagnoses were highly associated with Indonesia's rainy season (November-March). The polymerase chain reaction (PCR) assays on IS specimens captured most of the pathogens identified in this study.

Conclusions:

CAP in hospitalized Indonesian children is most commonly associated with mixed infections, with *H. influenzae* non-type B and RSV being the most frequently identified pathogens. Our study highlights the importance of PCR for diagnosis and by extension, appropriate use of antimicrobials.

Keywords: Pneumonia; Children; Indonesia; Etiology; Epidemiology

STRENGTHS AND LIMITATIONS OF THIS STUDY

- Prospective multisite study conducted over 27-months
- Used a comprehensive approach (culture, molecular, and paired serology assays) to identify causative pathogens from routinely collected specimens (blood, sputum, and nasopharyngeal swabs)
- Did not include healthy control children, limiting ability to estimate the adjusted population attributable fraction (aPaF) for each pathogen.
- Did not collect lung aspirates or pleural fluid specimens, which are preferred for determination of pneumonia etiology

For peer review only

For peer review only - http://bmjopen.bmj.com/site/about/guidelines.xhtml

- Several cases of pneumonia attributed to unknown etiology, which could be due to administration of antibiotics before culture, poor sputum quality, limited bacterial and viral panels used, lack of fungal testing or another factor

INTRODUCTION

Pneumonia is the leading infectious cause of child mortality. It accounts for approximately one million deaths annually among children under 5 years old, with a greater burden in low- and middle-income countries (LMICs).[1] In 2017, pneumonia contributed to 15% of childhood deaths and was the second highest cause of death amongst Indonesian children under five years.[2] One strategy to reduce child mortality due to pneumonia in Indonesia is through implementation of the Integrated Management of Childhood Illness (IMCI) guidelines which support early detection and management of possible pneumonia in young children with the goal of reducing deaths.[3,4]

IMCI is adapted from the World Health Organization (WHO) guideline, which was based on data from the 1970s through early 1990s when bacteria such as *Haemophilus influenzae* type b (Hib) and *Streptococcus pneumoniae* caused the majority of fatal pneumonias in children.[3,5,6] WHO and other practice guidelines, including the 2011 guidelines from the British Thoracic Society (BTS) and the Pediatric Infectious Diseases Society/Infectious Diseases Society of America (IDSA/PIDS), recommend empiric antibiotics as the first-line treatment to ensure that all potential pneumonia cases receive effective antibiotic therapy in an effort to decrease mortality.[7–9]

Several recent studies of community-acquired pneumonia (CAP) in children have highlighted the role of viral etiologies. Increased recognition of viral etiologies of CAP is likely due to both enhanced molecular diagnostic capacity and wide deployment of Hib and pneumococcal conjugate vaccines [PCV].[10,11] Treatment of non-bacterial pneumonia with antibiotics may engender avoidable antimicrobial resistance. Thus, current data on the etiologies of childhood pneumonia is needed and should be regularly evaluated to inform vaccination policies, empiric management decisions, and targeted treatment.[12]

From a diagnostic standpoint, direct demonstration of organisms by culture (or staining) of lung aspirates was the standard for determining the microbial etiology of CAP.[13] In the current era, many use less-invasive biological specimens (e.g. blood, naso/oropharyngeal secretions, bronchoalveolar lavage, or induced sputum) and employ diverse methods (e.g. culture, PCR, antigen detection, or paired serology) to identify organisms.[14] However, such comprehensive methods are costly and often require specialized equipment and human resources, limiting feasibility in low-resource settings.[15,16]

For peer review only - http://bmjopen.bmj.com/site/about/guidelines.xhtml

Prospective community-based cohort studies that define pathogen(s) causing CAP in Indonesian children are scarce. We conducted a "Partnerships for Enhanced Engagement in Research - Pneumonia in Pediatrics (PEER-PePPeS)" study, which aimed to identify etiologies of childhood CAP using comprehensive diagnostic methods.

METHODS

Study design and study sites

PEER-PePPeS was a multi-site observational cohort study seeking to determine etiologies of CAP amongst children aged 2–59 months in Indonesia. The study was conducted by the Indonesia Research Partnership on Infectious Disease (INA-RESPOND) and enrolled participants initially at three government referral teaching hospitals in three provinces: Kariadi Hospital (Central Java), Sardjito Hospital (Yogyakarta), and Tangerang District Hospital (Banten), as shown in **Supplementary Fig. 1.** Satellite sites located near the primary sites were added during the study to facilitate subject recruitment. An-Nisa Hospital was Tangerang District Hospital's satellite; Adhyatma Hospital and Bhakti Wira Tamtama Hospital served as satellites sites to Kariadi Hospital; Sardjito Hospital did not have a satellite. The Sardjito and Kariadi Hospitals are tertiary health care facilities equipped with 850+ beds each and Tangerang District Hospital with 437 beds. They have specialty physicians/departments and diagnostic laboratories that can perform routine hematological, biochemical, microbiological, molecular, and serological testing.

Study Definitions

In this study, pneumonia in children was defined as cough or fever with at least one of the following: shortness of breath (indicated by at least one of the following signs: head bobbing; nasal flaring; chest indrawing or intercostal retracting), tachypnea, grunting, crackles, rhonchi, decreased vesicular breath sounds, bronchial breath sounds or chest x-ray findings consistent with pneumonia. Tachypnea was defined as respiratory rate >50/min for infants 2–12 months and >40/min for children >12–60 months.[17] Abnormal chest x-ray findings consistent with pneumonia were defined as presence of either focal or diffuse infiltrates, a silhouette sign, pleural effusion, or air bronchogram.[18] Chest x-rays were read by the pediatrician.

Based on WHO classification and treatment of childhood pneumonia at health facilities (2014 version), for children 2–59 months of age, severe pneumonia was defined as pneumonia (tachypnea and/or chest indrawing) accompanied by presence of any danger signs, which including the inability

For peer review only - http://bmjopen.bmj.com/site/about/guidelines.xhtml

to drink, persistent vomiting, convulsions, lethargy or loss of consciousness, stridor in a calm child, or severe malnutrition.[17]

Study Participants

PEER-PePPeS study enrolled children aged 2-59 months, who were hospitalized between July 18th, 2017 until September 25th, 2019, and met the definition for pneumonia. Eligible subjects were enrolled within 24 hours of admission. Children were excluded if they had been hospitalized for >24 hours; had a malignancy or history of malignancy; a history of long term (≥2 months) steroid therapy; or conditions that might interfere with compliance with the study procedures (e.g., very ill patients for whom specimens could not be obtained or living outside the area for which follow-up was practical).

Study Procedures

Demographic and anthropometric data, current signs and symptoms, pregnancy history, vaccination status, breastfeeding history, antibiotic and steroid exposure, family history, medical history, risk factors, hematology profiles, chemistry results, and chest x-ray (per standard of care) were collected at enrollment. Clinical examination (vital signs, general examination, lung auscultation, SpO2); nasopharyngeal (NP) swab for molecular tests; induced sputum (IS) for culture and molecular tests; collection of blood specimens for routine blood count, cultures, molecular tests, serologic tests, C-reactive protein (CRP), and procalcitonin (PCT) were also performed. We prospectively followed subjects daily until hospital discharge; data on vital signs, respiratory signs, intensive care admission, intubation, complications and treatment were collected. On Day 14, we performed clinical examinations and collected convalescent sera for serology tests; subjects discharged before day 14 returned to clinic for their evaluation. We conducted a telephone interview on Day 30 (±4 days) to assess clinical outcome.

This study used several widely available bacterial and viral respiratory molecular pathogen panels and serologic assays.[19–22] NP and IS specimens were tested with a PCR panel that included twelve-viruses (Influenza A, Influenza B, Adenovirus, Enterovirus, Bocavirus, Respiratory Syncytial Virus (RSV) A, RSV B, Human Metapneumovirus, Rhinovirus, Parainfluenza Virus 1-4, Coronavirus OC43, and Coronavirus NL63). NP specimens were evaluated by PCR for five bacteria (*Haemophilus influenzae*, *Streptococcus pneumoniae*, *Moraxella catarrhalis*, *Staphylococcus aureus*, and *Klebsiella pneumoniae*), while IS specimens were tested for nine (*Haemophilus influenzae, Streptococcus pneumoniae, Mycoplasma pneumoniae, Chlamydia pneumoniae, Bordetella pertussis, Moraxella catarrhalis, Staphylococcus aureus*, *Klebsiella pneumoniae,* and *Legionella pneumoniae*). Good quality

For peer review only - http://bmjopen.bmj.com/site/about/guidelines.xhtml

(<10 squamous epithelial cells per low power field[12]) IS specimens underwent culture and gram stain.[23] For whole blood, qPCR was performed for three bacteria (*Haemophilus influenzae*, *Streptococcus pneumoniae*, and *Staphylococcus aureus)*. Serologic testing for seven viruses (Influenza A, Influenza B, Adenovirus, Parvovirus B19, Echovirus/Enterovirus, RSV, Parainfluenza Virus) and four bacteria (*Mycoplasma pneumoniae, Chlamydia pneumoniae, Legionella pneumoniae,* and *Bordetella pertussis*) was performed using paired acute-convalescent sera.

Blood culture, IS culture and Gram stain, routine blood count, CRP, PCT, and chest x-ray were performed by the laboratory/radiology department at the hospital site. qPCR and serology assays were performed at the INA-RESPOND Reference Laboratory located in Tangerang District Hospital. Details of blood culture, sputum culture, molecular and serology test techniques are shown in **Supplementary Table 1**.

Pathogen Identification

Causative bacterial, viral, or mixed pathogen for the pneumonia was determined based on an algorithm (PEER-PePPeS rules) for interpretation of microbiological, molecular, and serologic test results previously published.[12] In brief, we considered all organisms detected by blood culture, detected by whole blood PCR, or that grew from good quality IS specimen in high quantities with a compatible primary Gram stain as potential causative bacterial pathogens. Bacteria commonly considered contaminants were excluded. For the nasopharynx, potential colonizing bacteria (e.g. *H. influenzae*, *S. pneumoniae*, and *S. aureus*) and potential innocent bystander viruses (e.g. Bocavirus, Adenovirus, non-SARS human Coronavirus (hCoVs), Enterovirus, and Rhinovirus) were determined to be causative based on a PCR density cut-off and/or serodiagnosis criteria for paired acute and convalescent sera (seroconversion or a two to four-fold increase in antibody titers in the convalescent specimen).[12]

Data collection and statistical analysis

Data were recorded on paper case report forms and entered in duplicate into OpenClinica (OpenClinica, LLC, MA, USA) by research staff. Categorical variables were summarized using absolute values and percentages, and continuous variables as medians and interquartile ranges (IQRs). Differences in categorical variables and continuous variables were compared using the Pearson χ^2 test and Student's t-test, respectively. Statistical analyses were performed using Statistical Package for Social Science (SPSS) software version 23 (IBM Corporation, Armonk, NY, USA). All p-values were two-sided. Level of significance was set at $P < 0.05$.

For peer review only

For peer review only - http://bmjopen.bmj.com/site/about/guidelines.xhtml

Patient and public involvement statement

Patients or the public were not involved in study design or study conduct at any stage from inception to completion and dissemination of this project. Patients who met the eligibility criteria as described above were recruited to this study.

RESULTS

Study Population

Of 444 children who were hospitalized with CAP, 188 (42.3%) were eligible and enrolled in the study. Of 256 screening failures, 31.8% were due to hospitalization >24 hours at the time of screening and 22.1% to circumstances that might interfere with the study procedures. Of the 188 enrolled children, 184 (97.9%) had radiologic evidence of pneumonia. 179 (95.1%) subjects completed the study, including 19 (10.1%) who died. Eight subjects (4.3%) were lost to follow up, and one subject (0.5%) withdrew from the study. The study flow is shown in **Figure 1**.

Demographic and clinical characteristics are presented in **Table 1**. Overall, subject characteristics were similar across the three study sites. Median age was 9 months (IQR, 5 to 20), and 54.7% of subjects were male. The most common comorbid conditions were developmental delay (27.7%), congenital heart disease (26.1%), and severe malnutrition (18.6%). The percentage of subjects who had been vaccinated (age-adjusted) against pneumococcus, influenza, Hib-DPT, and measles vaccines were 4.8%, 6.4%, 56.4%, and 76.6%, respectively.

For peer review only

Table 1. Baseline Characteristics of Subjects.

Demographic Characteristics	All (N=188)	Semarang (N=47)	Yogyakarta (N=52)	Tangerang (N=89)
Age, median (IQR) months	9 (5 – 20)	9 (5.5 – 21)	8 (4 – 13.3)	11 (5-20)
Gender, Male (%)	103 (54.7)	29 (61.7)	26 (50)	48 (53.9)
Household Characteristics, (%):				
• Low Education of Parents*	163 (86.7)	37 (78.7)	42 (80.8)	84 (94.4)
• Living in a dense neighborhood†	121 (64.4)	19 (40.4)	42 (80.8)	60 (67.4)
• Sick household contact <14 days	109 (58.0)	22 (46.8)	43 (82.7)	44 (49.4)
• Exposure to cigarette smoke	120 (63.8)	24 (51.1)	27 (51.9)	69 (77.5)
• Attending daycare	4 (2.1)	2 (4.3)	1 (1.9)	1 (1.1)
Medical history (%):				
• Premature baby	34 (18.1)	4 (8.5)	16 (30.8)	14 (15.7)
• Low birth weight	46 (24.4)	12 (25.5)	20 (38.5)	14 (15.7)
• Developmental delay	52 (27.7)	16 (34.0)	21 (40.4)	15 (16.8)

For peer review only - http://bmjopen.bmj.com/site/about/guidelines.xhtml

• Congenital heart disease	49 (26.1)	16 (34.0)	24 (46.2)	9 (10.1)
• Severe malnutrition‡	35 (18.6)	10 (21.3)	13 (25.0)	12 (13.5)
• Neurological disorder	25 (13.3)	5 (10.6)	17 (32.7)	3 (3.4)
• Asthma	9 (4.8)	3 (6.4)	1 (1.9)	5 (5.6)
• HIV disease§	2 (1.1)	1 (2.1)	1 (1.9)	0 (0)
• Tuberculosis (recent/cured)	10 (5.3)	4 (8.5)	2 (3.8)	4 (4.5)
Immunization history, fully vaccinated for age‡ (%):				
• DPT-Hib	106 (56.4)	31 (66.0)	25 (48.1)	50 (56.2)
• Influenza	12 (6.4)	3 (6.4)	6 (11.5)	3 (3.5)
• Pneumococcus	9 (4.8)	3 (6.4)	5 (9.6)	1 (1.1)
• Measles	144 (76.6)	40 (85.1)	41 (78.8)	63 (70.8)
SpO_2 <90% and/or Cyanosis, (%)	43 (22.9)	7 (14.9)	17 (32.7)	19 (21.3)
Severe pneumonia (WHO Classification 2014 version), (%)	89 (47.3)	26 (55.3)	26 (50.0)	37 (41.6)
CXR Findings (%):				
• Pleural effusion	5 (2.7)	1 (2.1)	2 (3.8)	2 (2.2)
• Interstitial infiltrate	131 (69.7)	26 (55.3)	30 (57.7)	75 (84.3)
• Alveolar infiltrate	125 (66.5)	41 (87.2)	44 (84.6)	40 (44.9)
Antibiotic administration prior to blood collection for blood culture, (%)	150 (79.8)	39 (83.0)	49 (94.2)	62 (69.7)

*Low education of parents was defined by highest level of parents' formal education being high school diploma or less; †A densely populated neighborhood was defined as >200 people/km^2 or <8 m^2/person in the subject's home; ‡ Severe malnutrition was defined as weight for height below -3 standard deviations from the median of the WHO Child Growth Standards; §Subjects were tested for HIV infection if a parent / guardian provided consent and a specimen was available (n=160); #Full vaccination was defined as being up to date for age per vaccination schedule at study enrollment.

The most common symptoms were cough (91.0%), shortness of breath (90.6%), and fever (80.9%). Signs noted during the initial examination included intercostal retraction (91.0%), rhonchi (89.4%), and chest indrawing (66.5%). Of 188 subjects, 172 (91.4%) and 167 (88.8%) had CRP and PCT measured with median values of 9.0 (IQR, 3.6 – 28.0; Ref range ≤5) mg/L and 0.2 (IQR, 0.1 – 1.7; Ref range ≤0.15) ng/mL, respectively. Interstitial infiltrate (69.7%) was the most common radiographic finding. 47.3% of cases were classified as severe pneumonia according to the WHO classification system. All 188 enrolled cases were treated with antibiotics. The combination of ampicillin and gentamicin (37.8%), cefotaxime (26.1%), and ceftriaxone (19.1%) were the three most frequent regimens used during hospitalization (data not shown).

Detection of Pathogens

Blood and sputum cultures were performed on specimens from 184 (97.9%) and 183 (97.3%) subjects, respectively. A total of 150 (79.8%) children received antibiotics prior to collection of blood

For peer review only

For peer review only - http://bmjopen.bmj.com/site/about/guidelines.xhtml

for culture. Seventy-five (41.0%) sputum culture isolates were analyzed from specimens meeting the required quality criteria. A NP or OP swab was obtained from 187 (99.5%) subjects, IS for PCR from 176 (93.6%), whole blood for PCR from 163 (86.7%), and paired acute-convalescent serum specimens for serology from 116 (61.7%) (**Fig. 1**).

The PEER-PePPeS algorithm was used to determine the causative pathogen(s) from those identified by culture, molecular, and serologic assay. Amongst the 188 study participants, 48 (25.5%) had bacterial infection, 31 (16.5%) had viral infection, 76 (40.4%) were of mixed bacterial and viral etiology, and 33 (17.6%) were of unknown etiology (**Fig. 2, Panel A**). Mixed infection, the most common overall etiology, was seen in 38.7% of 2-11 month-olds and in 42.7% of 12-59 month-olds (**Fig 2. Panel B**). Mixed infection was also the predominant etiology across all study sites (**Supplementary Fig. 2**). *H. influenzae* non-type B (N=73), RSV (N=51), *K. pneumoniae* (N=43), *S. pneumoniae* (N=29), Influenza virus (N=25), *S. aureus* (N=20), PIV (N=17), hMPV (N=11), Rhinovirus (N=10), and *B. pertussis* (N=7) were the top ten pathogens identified, more commonly appearing in mixed infection as opposed to as a sole pathogen (**Fig 2. Panel C)**. Influenza virus was significantly higher in the age group 12-59 mo vs 2-11 mo (N=16, 64%, P=0.027), while *S. aureus* was significantly more common in 2-11 mo vs 12-59 mo (N=16, 80%, P=0.024). Though not statistically significant, other pathogens trended toward more frequent detection in age group 2-11 mo (except *B. pertussis*) (**Fig 2. Panel D**). Amongst 76 mixed infection cases, RSV + *H. influenzae* non-type B was the most common co-infection (N=22, 28.9%), followed by RSV + *S. pneumoniae* (N=10, 13.2%), Influenza virus + *H. influenzae* non-type B (N=10, 13.2%), RSV + *K. pneumoniae* (N=9, 11.8%), and Parainfluenza virus + *H. influenzae* non-type B (N=9, 11.8%) (Data not shown).

We observed no difference in pathogen distribution by pneumonia severity based on WHO classification system (**Supplementary Table 2 and Supplementary Fig. 3**). By pathogen, there was no significant difference in distribution between pneumonia severity status or mortality, except for *S. pneumoniae* which was found in significantly more severe cases using the WHO system (*P*=0.033) (**Supplementary Table 2**).

A comparison of positivity rates for each causative pathogen by detection method is shown in **Table 2**. Overall, PCR captured more bacterial pathogens than culture and more viral pathogens than acute-convalescent paired serology. Paired serology was generally helpful in identifying atypical bacteria, such as *C. pneumoniae* and *L. pneumophila*, and upper respiratory tract viruses, such as Rhinovirus and Enterovirus. When comparing blood and IS culture, IS yielded more positive bacterial pathogen results. Similarly, IS PCR captured more pathogens than NP/OP PCR.

Table 2. Causative Pathogens per PEER-PePPeS Rules by Detection Method

For peer review only - http://bmjopen.bmj.com/site/about/guidelines.xhtml

Pathogen	N	Blood culture (% N)	IS culture (% N)	Whole blood PCR (% N)	NP / OP PCR (% N)	IS PCR (% N)	Serology Test (% N)
**Gram-positive cocci bacteria**							
S. pneumoniae	29	1 (3.4%)	3 (10.3%)	0 (0.0%)	21 (72.4%)	28 (96.6%)	
S. aureus	20	0 (0.0%)	7 (35%)	0 (0.0%)	11 (55%)	19 (95%)	
S. mitis	4	0 (0.0%)	4 (100%)				
S. pyogenes	1	0 (0.0%)	1 (100%)				
**Gram-negative cocci bacteria**							
M catarrhalis	2	0 (0.0%)	2 (100%)		2 (100%)	2 (100%)	
**Gram-negative rods bacteria**							
H. inf non-type b	73	0 (0.0%)	0 (0.0%)	8 (10.9%)	60 (82.2%)	71 (98.6%)	
K. pneumoniae	43	0 (0.0%)	17 (39.5%)		2 (4.7%)	34 (79.1%)	
B. pertussis	7	0 (0.0%)	0 (0.0%)			7 (100%)	
E. coli	5	1 (20%)	4 (80%)				
P. aeruginosa	4	0 (0.0%)	4 (100%)				
A. baumannii	3	0 (0.0%)	3 (100%)				
H. inf type b	2	0 (0.0%)	0 (0.0%)	0 (0.0%)	0 (0.0%)	2 (100%)	
N. meningitidis	1	1 (100%)	1 (100%)				
**Atypical-bacteria**							
C. pneumoniae	5	0 (0.0%)	0 (0.0%)			0 (0.0%)	5 (100%)
M. pneumoniae	5	0 (0.0%)	0 (0.0%)			5 (100%)	1 (20%)
L. pneumophila	1	0 (0.0%)	0 (0.0%)			0 (0.0%)	1 (100%)
**Virus**							
RSV	51				36 (70.6%)	45 (88.2%)	10 (19.6%)
RSV A	15				10 (66.7%)	13 (86.7%)	
RSV B	36				26 (72.2%)	32 (88.8%)	
Influenza Virus	25				16 (64%)	22 (88%)	9 (36%)
Inf A (H1N1)	7				7 (100%)	7 (100%)	7 (70%)
Inf A (H3N2)	3				3 (100%)	3 (100%)	
Inf B	14				6 (42.9%)	12 (85.7%)	2 (14.3%)
PIV	17				16 (94.1%)	15 (88.2%)	3 (17.6%)
PIV 1	5				5 (100%)	4 (80%)	3 (17.6%)
PIV 2	0				0 (0.0%)	0 (0.0%)	
PIV 3	11				10 (90.9%)	10 (90.9%)	
PIV 4	1				1 (100%)	1 (100%)	
hMPV	11				5 (45.5%)	10 (90.9%)	
Rhinovirus	10				10 (100%)	6 (60%)	4 (40%)
Enterovirus	5				3 (60%)	3 (60%)	3 (60%)
Bocavirus	3				2 (66.7%)	3 (100%)	
CorNL63	2				2 (100%)	2 (100%)	

Grey-box indicates the assay was not performed

Mortality

Nineteen (10.1%) subjects died during the 30-day study period. Seven were male, and most (17 subjects) were less than 1 year old. Among deceased subjects, median study duration was 12 (IQR, 4 – 17.5) days; 8 were admitted to ICU, and 6 received mechanical ventilation. Twelve died due to

For peer review only - http://bmjopen.bmj.com/site/about/guidelines.xhtml

respiratory failure, three due to sepsis, and three for unknown reasons after discharge (data not shown). Most deaths occurred in the 2-11 mo age group compared with the 12-59 mo age group (78.9% vs. 21.1%, *p*=0.036). Infection of deceased subjects was bacterial-only in 7, viral-only in 2, mixed in 5, and unknown in 5 subjects, with no significant differences between alive and deceased subjects. *H. influenzae* non-type B was the most common pathogen identified in deceased subjects (N=8), followed by *K. pneumoniae* (N=6), influenza virus (N=3), *B. pertussis* (N=2), and RSV (N=2) (**Supplementary Table 2**). Some deceased subjects had pre-existing health conditions, most common were congenital heart disease (10 subjects), severe malnutrition (7 subjects), and developmental delay (7 subjects) (data not shown).

Seasonality

During the 27-month study period, infections caused by RSV and influenza were seen year-round with peak activity occurring during the wet season (November to March) in Indonesia (66.7%, p<0.001; and 64.0%, p=0.012, respectively). However, there was little variation in detection of the most common respiratory bacterial infections by month and season. *H. influenzae* non-type B shows peaks in August (N=12, 16.4%) and March (N=11, 15.1%), while *K. pneumoniae* and *S. pneumoniae* fluctuate at lower levels throughout the year (**Figure 3**).

DISCUSSION:

PEER-PePPeS, a prospective multisite study, addresses a critical knowledge gap about the current epidemiology of pathogens causing CAP in children 2-59 months old in Indonesia. The study found: (1) mixed bacterial and viral infection is the predominant cause of childhood CAP, irrespective of age group and pneumonia severity; (2) bacterial infections were common (66% of cases) with *H. influenzae* non-b type, *K. pneumoniae*, and *S. pneumoniae* as the three most common bacterial etiologies; (3) viral pathogens were also common (57% of PEER-PePPeS subjects), with 16.5% of cases attributed to virus only and RSV and Influenza Virus being the most common viruses identified; and (4) PCR on IS specimens was the most sensitive assay for pathogen identification in this study.

A mixed bacterial and viral infection was the most commonly identified etiology in our study. While this is consistent with findings from other studies the clinical significance of the mixed infection remains controversial and it is not clear whether or not both agents are acting as true pathogens.[20,24] PEER-PePPeS did not demonstrate a correlation of mixed infection with pneumonia severity and 30-day mortality. Many deceased cases occurred at a younger age (less than 1 year old), and in the presence of comorbidities, such as congenital heart disease and severe malnutrition, as also

For peer review only - http://bmjopen.bmj.com/site/about/guidelines.xhtml

shown by previous reports.[25,26] Such factors may need to be considered in the prevention and management of childhood pneumonia to reduce mortality rate.

In recent years, there has been an increased focus on the role of respiratory viruses in childhood pneumonia, partly due to the reduction in bacterial disease associated with the use of conjugate pneumococcal and Hib vaccines and the increased capacity to detect viruses through PCR methods.[19,20,27,28] In PEER-PePPeS, viruses were found in 57% of subjects (virus only + mixed infection), with RSV and influenza virus being the viruses most commonly detected. RSV and influenza infection may be associated with Indonesia's wet/rainy season, consistent with other reports from tropical regions.[29–31] A high prevalence of RSV was also observed in the GABRIEL and PERCH international case-control studies of childhood pneumonia etiology.[20,28] In terms of mixed infections, we found that RSV + *H. influenzae* non-type B and RSV + *S. pneumoniae* were most common. Since respiratory viruses such as RSV can predispose to secondary bacterial infections, particularly *S. pneumoniae* and *H. influenzae*[32], and conversely bacteria can increase RSV susceptibility[32,33], these co-infections highlight the need for optimizing RSV surveillance, prevention and treatment.

Though Influenza virus also increases risk for secondary bacterial infections and is a major cause of childhood morbidity and mortality worldwide, data from developing countries is scarce.[34] In a previous Indonesian study of hospitalized patients with a severe acute respiratory infection (SARI), the prevalence of the Influenza virus was 10.6% in children less than 5 years old, and was never diagnosed during hospitalization.[35] PEER-PePPeS confirms the need for improved diagnostic strategies, management optimization, and influenza vaccination in children. Of note, our study was conducted before identification of COVID-19 in Indonesia[36], so did not address the role of COVID-19 in childhood pneumonia.

We also found that 66% of cases were caused by bacterial infection (bacteria only + mixed infection). Overall, *H. influenzae* non-type B was the most common bacteria implicated, followed by *K. pneumoniae* and *S. pneumoniae*. *H. influenzae* non-type B predominance was also observed in a Malaysian study, where 90% of enrolled children were vaccinated against Hib as part of the national immunization program.[22] With our moderate (56.4%) Hib vaccine coverage, high incidence of *H. influenzae* non-type B may represent true prevalence or strains not covered by Hib vaccine.[37] This finding agrees with current data that non-typeable *H. influenzae* (NTHi) can cause significant illness, and argues for strengthening the diagnostic laboratory capacity for pediatric specimens.

Identification of *K. pneumoniae* as the second most common bacterial etiology of childhood CAP is consistent with previously reported high carriage rates (~7%) in healthy Indonesian children. Carriage has been related to poor water and food hygiene and may give rise to pneumonia, especially

For peer review only - http://bmjopen.bmj.com/site/about/guidelines.xhtml

in children with malnutrition.[38] Given *K. pneumoniae's* potential for antibiotic resistance and high virulence of some strains, proactive detection and management strategies should be prioritized.[39]

The relatively low prevalence (15.4%) of *S. pneumoniae* in PEER-PePPes was surprising since carriage rates are high and PCV coverage low in Indonesia.[40] A similar relatively low prevalence of *S. pneumoniae* in childhood CAP was also reported from Malaysia, where PCV coverage is 8.7%[22] and in the PERCH study, reflecting temporal shifts in childhood pneumonia etiologies.[20] As only 4.8% of PEER-PePPeS subjects had received PCV, vaccination alone cannot account for the low *S. pneumoniae* prevalence. It is possible that antibiotic exposure prior to obtaining specimens may reduce median colonization density and lower positive findings yield of *S. pneumoniae* by both culture and PCR.[41] Moreover, our panel did not include *S. pneumoniae* paired serology, which may be useful to increase pneumococcal diagnosis in young children.[42] Nonetheless, *S. pneumoniae* remains an important etiological agent of severe/complicated CAP globally.[43] Our finding that *S. pneumoniae* was significantly associated with severe cases by the WHO classification system supports the need for ongoing surveillance, vaccination and prevention of transmission between adults and children.

Inclusion of several pathogen identification strategies in PEER-PePPes demonstrates the differential utility of assays and specimen types. Our findings highlight the value of molecular assays, especially in culture-negative cases where microorganisms may be nonrecoverable in culture due to prior antibiotics or presence of otherwise hard to culture bacteria.[44,45] PCR is also less laborious and boasts a shorter turn-around-time than conventional culture. PCR can additionally identify genes associated with antibiotic resistance, though conventional culture methods will still be required to confirm the phenotypic resistance.[46,47] Even with the limited PCR panels used in our study, molecular assays had greater sensitivity for identification of bacterial pathogens than blood or sputum culture when using the PEER-PePPeS rules. Regardless of the method of detection, targeted treatment can follow empiric treatment once an organism is identified. This facilitates optimization of management while minimizing risk of adverse events and development of antimicrobial resistance due to unnecessary, prolonged use of antibiotics.

Although sensitive for detection, PCR does not provide information regarding infectiousness or viability. Genome fragments from dead organisms may be detected, often at a low level, even after clinical resolution.[45] Furthermore, negative results may occur due to differential viral kinetics along the respiratory tract. Thus lower respiratory tract specimens, such as IS, should be sought as they originate from the actual site of infection.[10,12] Accordingly, we observed a higher yield from PCR on IS than NP specimens. This finding may be confounded as IS and NP detection panels varied slightly. We also found that the use of paired serologies increased the diagnostic yield and was useful for

For peer review only - http://bmjopen.bmj.com/site/about/guidelines.xhtml

pathogen confirmation, particularly in the setting of innocent bystander viruses and atypical bacteria.[12]

PEER-PePPeS used a comprehensive approach for pathogen detection to increase diagnostic yield. It also enrolled patients over a 27-month study period, facilitating assessment of seasonality. However, our study has several limitations. The relatively small sample size and geographic limitation to the island of Java may limit generalizability. Second, we did not enroll healthy control children, limiting the ability to estimate the adjusted population attributable fraction (aPaF) of each pathogen.[27,28] A healthy control group would have revealed the baseline carriage rate, minimizing over-attribution of disease to non-pathogenic organisms.[19,20,27,28] Third, we did not collect lung aspirates or pleural fluid specimens, which are superior for determination of pneumonia etiology. [13] Fourth, several subjects had pneumonia of unknown etiology; this may have been due to administration of antibiotics before culture, poor IS quality, the limited panel of bacterial and viral pathogens tested, lack of fungal testing, or currently unrecognized causes of pediatric pneumonia.

In conclusion, the epidemiology of childhood CAP is constantly evolving in step with social and environmental factors and thus, should be regularly assessed. Our study found that *H. influenzae* non-type B and RSV were the most common pathogens causing hospitalized CAP among Indonesian children aged 2-59 months old, suggesting a changing pathogen profile from the 1970-1990s etiology studies that mainly detected *S. pneumoniae* and *H. influenzae* type B as the most important causes of childhood pneumonia in LMICs[3,5,6]. PCR on IS had the best sensitivity for pathogen identification, highlighting the need for accessible multiplexed point-of-care molecular assays to guide management. Optimization of pathogen detection to understand changing childhood CAP epidemiology will inform public policy on prevention and management.

ACKNOWLEDGMENTS:

We are grateful to the parents and children who participated in this study. We thank the site study teams (Isabella Puspa Dewi, Fenny Aztari, Mohammad Rosyid Ridho, Aisyah Pratiwi, Niken Maretasari, Firsty Ilminova, Yanantri Binga Ramsif, Yuli Mawarti, Nenes Prastiwi, and Yanantri Binga Ramsif), INA-RESPOND Reference Laboratory team (Gustiani, Deni Pepy, Wahyu Nawang Wulan, Rizki Amalia Sari, Yuanita Djajady, and Ungke Anton Jaya), US-NIAID and Indonesia NIHRD. We appreciate the collaboration from the hospital Directors at Tangerang District Hospital, An-Nisa Hospital, Kariadi Hospital, Adhyatma Hospital, Bhakti Wira Tamtama Hospital, and Sardjito Hospital. We also would like to thank Aly Diana for her assistance during manuscript submission.

For peer review only

For peer review only - http://bmjopen.bmj.com/site/about/guidelines.xhtml

CONTRIBUTORS:

DL, HF, RT, YM, HK, AMN, ATA, CYL, HCL designed and conceptualized the study. DL, HF, RT, AB, C, MSA, DW, M, SD, AS performed clinical assessments and were responsible for data entry. DL, HF, RT, YM, HK, AMN, NL, AK, CYL designed the methodology for pathogen identification. YM, HK, AMN performed data analysis, interpretation and drafted the first manuscript. DL, HK, ATA, MK, AN, CYL, HCL assisted with manuscript writing, analysis, and interpretation of data. All authors contributed to manuscript development, edited for critical content, and have approved the final version.

FUNDING STATEMENT:

This manuscript has been funded in whole or in part with MoH Indonesia, National Academy of Sciences (Sub-Grant Number: 2000007599), and Federal funds from the NIAID, NIH, under contract Nos. HHSN261200800001E and HHSN261201500003I. The content of this publication does not necessarily reflect the views or policies of the Department of Health and Human Services, nor does mention of trade names, commercial products, or organizations imply endorsement by the U.S. Government.

COMPETING INTERESTS:

The authors declare that the research was conducted in the absence of any commercial or financial relationships that could be construed as a potential conflict of interest.

ETHICS APPROVAL:

This study was approved by the Ethical Clearance Committee of Faculty of Medicine, Universitas Indonesia (No. 567/UN2.F1/ETIK/2017). The study was conducted in accordance with the Declaration of Helsinki. Written informed consent was obtained from parents or guardians before enrollment.

DATA AVAILABILITY STATEMENT:

Data are available upon reasonable request. The anonymized data set will be shared following the signing of a data-sharing agreement, with permission of the ethical clearance committee, study authors, and all project partners, exclusively for non-commercial purposes. Please contact the corresponding author with any queries.

For peer review only

For peer review only - http://bmjopen.bmj.com/site/about/guidelines.xhtml

REFERENCES:

1 McAllister DA, Liu L, Shi T, *et al.* Global, regional, and national estimates of pneumonia morbidity and mortality in children younger than 5 years between 2000 and 2015: a systematic analysis. *The Lancet Global health* 2019;**7**:e47–57. doi:10.1016/S2214-109X(18)30408-X

2 Windi R, Efendi F, Qona'ah A, *et al.* Determinants of acute respiratory infection among children under-five years in Indonesia. *Journal of pediatric nursing* 2021;**S0882**-**5963**:00080–4. doi:10.1016/j.pedn.2021.03.010

3 Reñosa MD, Dalglish S, Bärnighausen K, *et al.* Key challenges of health care workers in implementing the integrated management of childhood illnesses (IMCI) program: a scoping review. *Global Health Action* 2020;**13**:1732669. doi:10.1080/16549716.2020.1732669

4 Sulistyawati S, Sofiana L, Amala SK, *et al.* Pneumonia a neglected disease: A mixed-method study on the case-finding program in Indonesia. *AIMS public health* 2020;**7**:81. doi:10.3934/publichealth.2020008

5 Shann F. Etiology of severe pneumonia in children in developing countries. *Pediatric infectious disease* 1986;**5**:247–52. doi:10.1097/00006454-198603000-00017

6 Feikin DR, Hammitt LL, Murdoch DR, *et al.* The Enduring Challenge of Determining Pneumonia Etiology in Children: Considerations for Future Research Priorities. *Clinical Infectious Diseases* 2017;**64**:S188–96. doi:10.1093/cid/cix143

7 Bradley JS, Byington CL, Shah SS, *et al.* The Management of Community-Acquired Pneumonia in Infants and Children Older Than 3 Months of Age: Clinical Practice Guidelines by the Pediatric Infectious Diseases Society and the Infectious Diseases Society of America. *Clinical Infectious Diseases* 2011;**53**:e25–76. doi:10.1093/cid/cir531

8 Harris M, Clark J, Coote N, *et al.* British Thoracic Society guidelines for the management of community acquired pneumonia in children: update 2011. *Thorax* 2011;**66**:ii1 LP-ii23. doi:10.1136/thoraxjnl-2011-200598

9 Mathur S, Fuchs A, Bielicki J, *et al.* Antibiotic use for community-acquired pneumonia in neonates and children: WHO evidence review. *Paediatrics and international child health* 2018;**38**:S66–75. doi:10.1080/20469047.2017.1409455

10 Mardian Y, Kosasih H, Karyana M, *et al.* Review of Current COVID-19 Diagnostics and Opportunities for Further Development. Frontiers in Medicine. 2021;**8**:562. doi:10.3389/fmed.2021.615099

For peer review only - http://bmjopen.bmj.com/site/about/guidelines.xhtml

11. Levine OS, O'Brien KL, Deloria-Knoll M, *et al.* The Pneumonia Etiology Research for Child Health Project: A 21st Century Childhood Pneumonia Etiology Study. *Clinical Infectious Diseases* 2012;**54**:S93–101. doi:10.1093/cid/cir1052
12. Mardian Y, Menur Naysilla A, Lokida D, *et al.* Approach to Identifying Causative Pathogens of Community-Acquired Pneumonia in Children Using Culture, Molecular, and Serology Tests. Frontiers in Pediatrics. 2021;**9**:498. doi:10.3389/fped.2021.629318
13. Hammitt LL, Murdoch DR, Scott JAG, *et al.* Specimen collection for the diagnosis of pediatric pneumonia. *Clinical infectious diseases : an official publication of the Infectious Diseases Society of America* 2012;**54 Suppl 2**:S132–9. doi:10.1093/cid/cir1068
14. Zar HJ, Andronikou S, Nicol MP. Advances in the diagnosis of pneumonia in children. *BMJ (Clinical research ed)* 2017;**358**:j2739. doi:10.1136/bmj.j2739
15. Thomas J, Pociute A, Kevalas R, *et al.* Blood biomarkers differentiating viral versus bacterial pneumonia aetiology: a literature review. *Italian Journal of Pediatrics* 2020;**46**:4. doi:10.1186/s13052-020-0770-3
16. Jiao F, Chen J, Wang M, *et al.* Determination of Procalcitonin, C-Reactive Protein and White Blood Cell Levels to Diagnose Community-Acquired Pneumonia (CAP). *The Indian Journal of Pediatrics* 2019;**86**:763. doi:10.1007/s12098-019-02938-z
17. World Health Organization. Revised WHO classification and treatment of childhood pneumonia at health facilities: evidence summaries. *Geneva: World Health Organization* 2014.
18. Walker CM, Abbott GF, Greene RE, *et al.* Imaging pulmonary infection: classic signs and patterns. *AJR American journal of roentgenology* 2014;**202**:479–92. doi:10.2214/AJR.13.11463
19. Jain S, Williams DJ, Arnold SR, *et al.* Community-Acquired Pneumonia Requiring Hospitalization among U.S. Children. *New England Journal of Medicine* 2015;**372**:835–45. doi:10.1056/NEJMoa1405870
20. Group PER for CH (PERCH) S. Causes of severe pneumonia requiring hospital admission in children without HIV infection from Africa and Asia: the PERCH multi-country case-control study. *Lancet (London, England)* 2019;**394**:757–79. doi:10.1016/S0140-6736(19)30721-4
21. Jansen RR, Schinkel J, Koekkoek S, *et al.* Development and evaluation of a four-tube real time multiplex PCR assay covering fourteen respiratory viruses, and comparison to its corresponding single target counterparts. *Journal of clinical virology : the official publication of the Pan American Society for Clinical Virology* 2011;**51**:179–85. doi:10.1016/j.jcv.2011.04.010

For peer review only - http://bmjopen.bmj.com/site/about/guidelines.xhtml

22 Nathan AM, Teh CSJ, Jabar KA, *et al.* Bacterial pneumonia and its associated factors in children from a developing country: A prospective cohort study. *PloS one* 2020;**15**:e0228056. doi:10.1371/journal.pone.0228056

23 Lahti E, Peltola V, Waris M, *et al.* Induced sputum in the diagnosis of childhood community-acquired pneumonia. *Thorax* 2009;**64**:252 LP – 257. doi:10.1136/thx.2008.099051

24 Honkinen M, Lahti E, Österback R, *et al.* Viruses and bacteria in sputum samples of children with community-acquired pneumonia. *Clinical Microbiology and Infection* 2012;**18**:300–7. doi:https://doi.org/10.1111/j.1469-0691.2011.03603.x

25 Dean P, Florin TA. Factors Associated With Pneumonia Severity in Children: A Systematic Review. *Journal of the Pediatric Infectious Diseases Society* 2018;**7**:323–34. doi:10.1093/jpids/piy046

26 Chen L, Miao C, Chen Y, *et al.* Age-specific risk factors of severe pneumonia among pediatric patients hospitalized with community-acquired pneumonia. *Italian Journal of Pediatrics* 2021;**47**:100. doi:10.1186/s13052-021-01042-3

27 Bhuiyan MU, Snelling TL, West R, *et al.* The contribution of viruses and bacteria to community-acquired pneumonia in vaccinated children: a case-control study. *Thorax* 2019;**74**:261 LP – 269. doi:10.1136/thoraxjnl-2018-212096

28 Bénet T, Sánchez Picot V, Messaoudi M, *et al.* Microorganisms Associated With Pneumonia in Children <5 Years of Age in Developing and Emerging Countries: The GABRIEL Pneumonia Multicenter, Prospective, Case-Control Study. *Clinical infectious diseases : an official publication of the Infectious Diseases Society of America* 2017;**65**:604–12. doi:10.1093/cid/cix378

29 Kosasih H, Klimov A, Xiyan X, *et al.* Surveillance of Influenza in Indonesia, 2003–2007. *Influenza and Other Respiratory Viruses* 2013;**7**:312–20. doi:https://doi.org/10.1111/j.1750-2659.2012.00403.x

30 Suryadevara M, Domachowske JB. Epidemiology and Seasonality of Childhood Respiratory Syncytial Virus Infections in the Tropics. Viruses. 2021;**13**. doi:10.3390/v13040696

31 Hirve S, Newman LP, Paget J, *et al.* Influenza Seasonality in the Tropics and Subtropics – When to Vaccinate? *PLOS ONE* 2016;**11**:e0153003.https://doi.org/10.1371/journal.pone.0153003

32 Griffiths C, Drews SJ, Marchant DJ. Respiratory syncytial virus: Infection, detection, and new options for prevention and treatment. *Clinical Microbiology Reviews* 2017;**30**:277–319. doi:10.1128/CMR.00010-16

33 Gulraiz F, Bellinghausen C, Bruggeman CA, *et al.* Haemophilus influenzae increases the

For peer review only

For peer review only - http://bmjopen.bmj.com/site/about/guidelines.xhtml

susceptibility and inflammatory response of airway epithelial cells to viral infections. *FASEB journal : official publication of the Federation of American Societies for Experimental Biology* 2015;**29**:849–58. doi:10.1096/fj.14-254359

34 Dananché C, Sánchez Picot V, Bénet T, *et al.* Burden of Influenza in Less Than 5-Year-Old Children Admitted to Hospital with Pneumonia in Developing and Emerging Countries: A Descriptive, Multicenter Study. *The American journal of tropical medicine and hygiene* 2018;**98**:1805–10. doi:10.4269/ajtmh.17-0494

35 Aman AT, Wibawa T, Kosasih H, *et al.* Etiologies of severe acute respiratory infection (SARI) and misdiagnosis of influenza in Indonesia, 2013-2016. *Influenza and Other Respiratory Viruses* 2021;**15**:34–44. doi:https://doi.org/10.1111/irv.12781

36 Setiawaty V, Kosasih H, Mardian Y, *et al.* The Identification of First COVID-19 Cluster in Indonesia. *The American Journal of Tropical Medicine and Hygiene*;**103**:2339–42. doi:10.4269/ajtmh.20-0554

37 Agrawal A, Murphy TF. Haemophilus influenzae infections in the H. influenzae type b conjugate vaccine era. *Journal of clinical microbiology* 2011;**49**:3728–32. doi:10.1128/JCM.05476-11

38 Helmia F, A. SJ, Hussein GM, *et al.* Nasopharyngeal Carriage of Klebsiella pneumoniae and Other Gram-Negative Bacilli in Pneumonia-Prone Age Groups in Semarang, Indonesia. *Journal of Clinical Microbiology* 2013;**51**:1614–6. doi:10.1128/JCM.00589-13

39 Pranavathiyani G, Prava J, Rajeev AC, *et al.* Novel Target Exploration from Hypothetical Proteins of Klebsiella pneumoniae MGH 78578 Reveals a Protein Involved in Host-Pathogen Interaction . Frontiers in Cellular and Infection Microbiology . 2020;**10**:109.https://www.frontiersin.org/article/10.3389/fcimb.2020.00109

40 Farida H, Severin JA, Gasem MH, *et al.* Nasopharyngeal Carriage of Streptococcus pneumonia in Pneumonia-Prone Age Groups in Semarang, Java Island, Indonesia. *PLOS ONE* 2014;**9**:e87431.https://doi.org/10.1371/journal.pone.0087431

41 Driscoll AJ, Deloria Knoll M, Hammitt LL, *et al.* The Effect of Antibiotic Exposure and Specimen Volume on the Detection of Bacterial Pathogens in Children With Pneumonia. *Clinical Infectious Diseases* 2017;**64**:S368–77. doi:10.1093/cid/cix101

42 Andrade DC, Borges IC, Vilas-Boas AL, *et al.* Infection by Streptococcus pneumoniae in children with or without radiologically confirmed pneumonia. *Jornal de pediatria* 2018;**94**:23–30. doi:10.1016/j.jped.2017.03.004

43 Principi N, Esposito S. Management of severe community-acquired pneumonia of children in developing and developed countries. *Thorax* 2011;**66**:815–22. doi:10.1136/thx.2010.142604

For peer review only

44 Lleo MM, Ghidini V, Tafi MC, *et al.* Detecting the presence of bacterial DNA by PCR can be useful in diagnosing culture-negative cases of infection, especially in patients with suspected infection and antibiotic therapy. *FEMS microbiology letters* 2014;**354**:153–60. doi:10.1111/1574-6968.12422

45 Torres A, Lee N, Cilloniz C, *et al.* Laboratory diagnosis of pneumonia in the molecular age. *European Respiratory Journal* 2016;**48**:1764 LP – 1778. doi:10.1183/13993003.01144-2016

46 Morozumi M, Chiba N, Ubukata K, *et al.* Antibiotic susceptibility in relation to genotype of Streptococcus pneumoniae, Haemophilus influenzae, and Mycoplasma pneumoniae responsible for community-acquired pneumonia in children. *Journal of Infection and Chemotherapy* 2013;**19**:432–40. doi:https://doi.org/10.1007/s10156-012-0500-x

47 Aydemir O, Aydemir Y, Ozdemir M. The role of multiplex PCR test in identification of bacterial pathogens in lower respiratory tract infections. *Pakistan journal of medical sciences* 2014;**30**:1011–6. doi:10.12669/pjms.305.5098

FIGURE CAPTIONS:

Figure 1. Subject screening, enrolment, and monitoring flowchart. CAP, community-acquired pneumonia; RR, respiratory rate; CXR, chest X-Ray; CRP, C-reactive protein; PCT, procalcitonin; NP, nasopharyngeal; IS, induced sputum; PCR, polymerase chain reaction.

Figure 2. Pathogen Distribution. (A) Overall proportion of identified viral/bacterial/mixed pathogen, (B) Viral/bacterial/mixed pathogens by age group, (C) Pattern of detection of the ten most identified pathogens, (D) Distribution of ten most identified pathogens by age group. *P<0.05

Figure 3. Distribution of the (A) monthly count and (B) seasonal pattern of infection caused by *H. influenzae* non-type B, RSV, *K. pneumoniae*, *S. pneumoniae*, and Influenza virus during a 27-month study period.

SUPPLEMENTARY INFORMATION:

Details of Microbiological, Molecular and Serologic Methods, **Supplementary Table 1**

Pathogen distribution by WHO severity classification status and mortality, **Supplementary Table 2**

PEER-PePPeS Study sites, **Supplementary Figure 1**

Proportion of Identified Pathogen in each Sites, **Supplementary Figure 2**

Proportion of Identified Pathogen between WHO Severity Status, **Supplementary Figure 3**

For peer review only

For peer review only - http://bmjopen.bmj.com/site/about/guidelines.xhtml

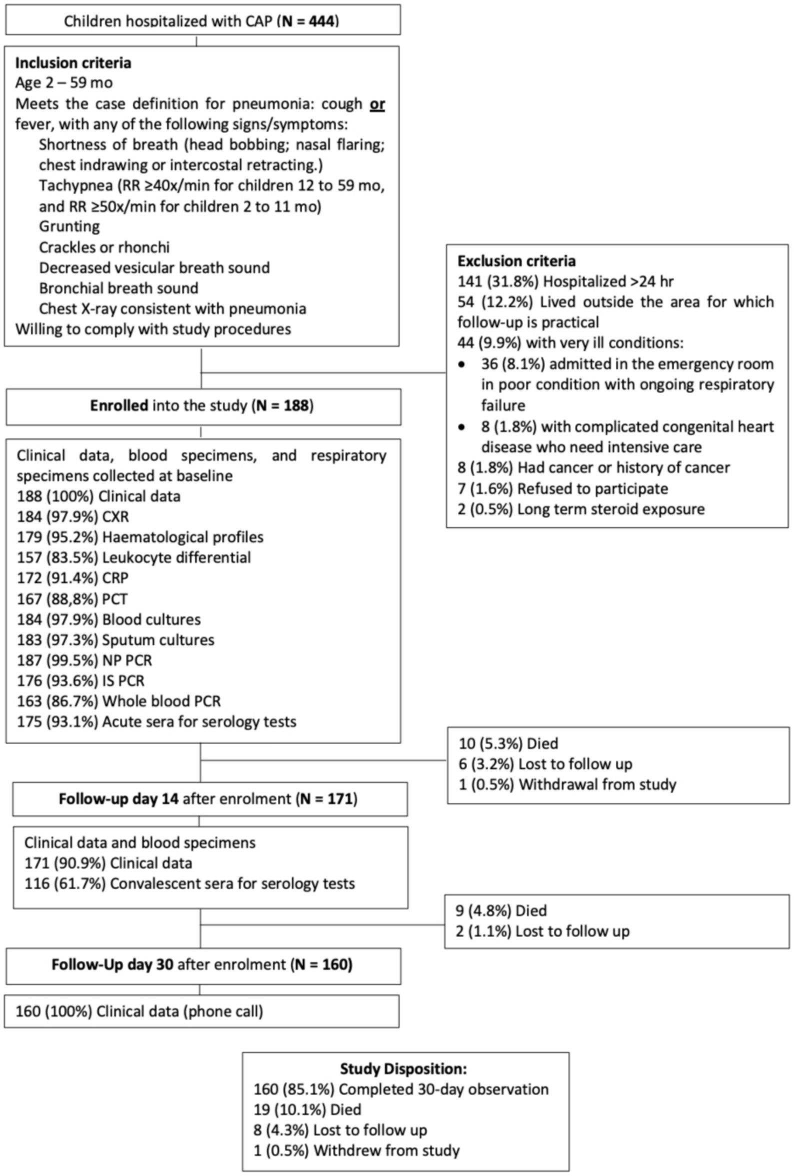

254x381mm (300 x 300 DPI)

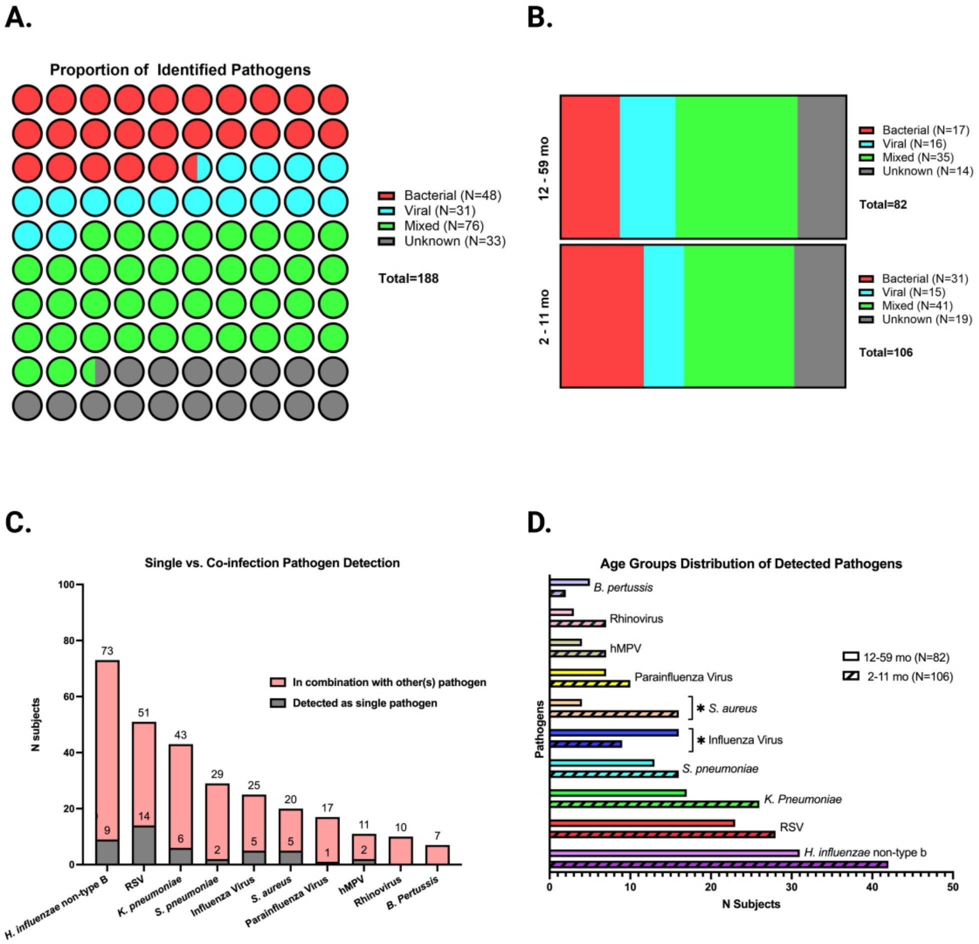

381x381mm (300 x 300 DPI)

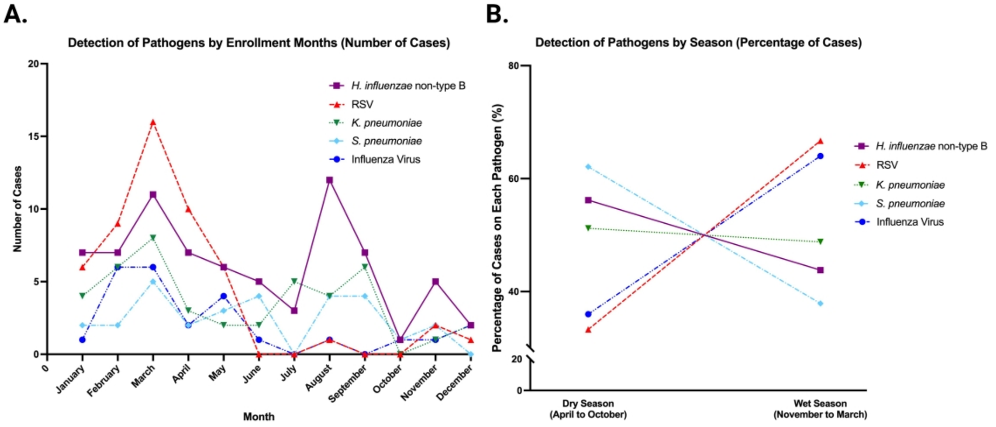

381x177mm (300 x 300 DPI)

SUPPLEMENTARY MATERIALS.

Supplementary Table 1. Microbiological, Molecular and Serologic Methods

No.	Assays	Procedures
1.	Gram stain	Gram-stained smears were obtained from the most purulent portion of each induced sputum specimen. The good quality specimen was defined as <10 squamous epithelium per low-power field (magnification, 100×) [1]. The procedure of the Gram stain required four basic steps that include applied a primary stain (crystal violet) to a heat-fixed smear, followed by the addition of a mordant (Gram's Iodine), rapid decolorization with alcohol, acetone, or a mixture of alcohol and acetone and lastly, counterstained with safranin [2]. The Gram-stained smears interpreted as follows: Gram-positive lancet-shaped diplococci (GPDC) suggest *Streptococcus pneumoniae*; Gram-positive diplococci (GPDC) or cocci in chains suggest *Streptococcus pyogenes*; Gram-positive cocci in clusters (GPC-cluster) suggest *Staphylococcus aureus*; Gram-negative coccobacilli (GNCB) suggest *Hemophilus influenzae*, *Bordetella pertussis* or *Acinetobacter baumannii*; Gram-negative diploccoci (GNDC) suggest *Moraxella catarrhalis*; large Gram-negative rods (GNR-large) suggest *Klebsiella pneumoniae* or *Escherichia coli*; and small Gram-negative rods (GNR-small) suggest *Pseudomonas aeruginosa* [3].
2.	Induced Sputum Culture	The most purulent portion of induced sputum was inoculated onto sheep blood, chocolate, and MacConkey agars, streaked out using a standard 4-quadrant streaking method, and incubated at 35°C for 48 hours. Cultures were examined at 24 hours and 48 hours, and predominant bacteria were identified and quantified according to the farthest quadrant with visible colonies (first quadrant, scanty; second quadrant, 1+; third quadrant, 2+; fourth quadrant, 3+) [4]. Then, the predominant bacteria isolates were inoculated into the appropriate VITEK identification strip using the VITEK® 2 COMPACT (BioMérieux, Germany). Briefly, a bacterial suspension was adjusted to a McFarland standard of 0.50 in a solution of 0.45 % sodium chloride using DensiLameter. The time between preparation of the solution and filling of the card was always less than 1 h. Analysis was done using the identification card and automatically read every 15 min. Bacteria identification and antibiotic susceptibility testing results were analyzed using the VITEK 2 software according to the manufacturer's instructions [5].
3.	Blood Culture	Up to 2 mL of blood samples (2 bottle sets) were collected and sent to the site laboratory with standardized procedures. Blood cultures were incubated for at least 5 days, unless positive, using automated systems (BacT/ALERT in Tangerang Hospital; BACTEC at other sites) [6]. Organisms were identified according to standard microbiological methods as described in induced sputum culture section. The following organisms were considered to be contaminants when identified in blood cultures: Coagulase-negative *staphylococci*, *Micrococcus* spp., *Propionibacterium* spp., Alpha-hemolytic streptococci (except

For peer review only

		pneumococcus, *Streptococcus anginosus*, and *Streptococcus mitis*), *Enterococcus* spp., *Corynebacterium* spp. (diphtheroids), *Bacillus* spp. (except *Bacillus anthracis*), *Pseudomonas* spp. (except *Pseudomonas aeruginosa*), *Stomatococcus, Aeroccocus, Neiserria subflava, Veillonella* spp., other environmental non-fermenting Gram negative rods, and *Candida* spp [7].
4.	Viral RNA Extraction	Viral RNA was extracted from viral transport media (VTM) containing respiratory swab as well as sputum, using the QIAamp Viral RNA Mini Kit (Qiagen, Hilden, Germany) according to the manufacturer's protocol. Briefly, 140 µl of VTM or sputum coat was lysed in 560 of carrier RNA-containing AVL buffer, followed by the binding of viral RNA to the QIAamp membrane. Contaminants were removed from viral RNA in two separate washing steps using two different wash buffers, AW1 and AW2. Viral RNA was eluted in 60 µl of AVE buffer and kept in -80° C if not directly used [8,9].
5.	Bacterial DNA Extraction	Bacterial DNA was extracted from viral transport media (VTM) containing respiratory swab as well as sputum, using the QIAamp® DNA Mini Kit (Qiagen, Hilden, Germany) according to the manufacturer's protocol. Briefly, 20 µl of QIAGEN Protease and 200 µl of VTM or sputum coat was lysed in 200 of AL buffer, followed binding of DNA to the QIAamp membrane. Contaminants were removed from DNA in two separate washing steps using two different wash buffers, AW1 and AW2. Bacterial DNA was eluted in 200 µl of AE buffer and kept in -80° C if not directly used
6.	qPCR for Respiratory Viruses	The realtime PCR for respiratory virus detection was done followed the protocol of Beld et al., 2004 and Jansen et al., 2011. Positive control is a synthetic plasmid carrying the nucleotide sequence of the detection target. Primers, probes, and positive controls were synthesized and purified by an outside vendor (Integrated DNA Technologies, Iowa, US). Realtime PCR was done using the TaqManTM Fast Virus 1-Step Master Mix (Thermo Fisher Scientific; Cat#: 4444432) in an Applied Biosystems 7500 Fast Realtime PCR System (Thermo Fisher Scientific, MA, US). The reaction mixture composition was 1X TaqManTM Fast Virus 1-Step Master Mix, 0.5 µM of each primer, 0.25 µM probe, and 4 µl RNA, in a total 20 µl volume. The cycle condition was 50° reverse transcription for 5 minutes, 95° C initial denaturation for 20 seconds, followed by 45 cycles of denaturation (95° C, 3 seconds) and annealing/elongation (55° C, 30 seconds). Realtime PCR works correctly when the positive control demonstrates the amplification curve and the template-free (negative) control demonstrates no amplification curve (no Ct values) [8,9].
7.	qPCR for Respiratory Bacteria	In real-time PCR (qPCR) a portion of bacterial DNA genome specific to the pathogen(s) of interest is amplified using a specific pair of primers and probes for each bacteria, that were selected from the available literature [10–14]. A detector (TaqMan® probe) is used in the reaction. Mastermix is prepared in a 1.5-ml tube for total reaction. qPCR assays were carried out in a total volume of 20 µL, comprising 10 µL of TaqMan® Fast Universal PCR Master Mix, 1.4 µL of nuclease-free water (Promega), 3.6 µL of oligonucleotide mixtures, and 4 µL of DNA extract. The cycle condition was 95° C initial denaturation for 20 seconds, followed by

For peer review only

		45 cycles of denaturation (95º C, 3 seconds) and annealing/elongation (58º C, 30 seconds). Realtime PCR works correctly when the positive control demonstrates the amplification curve and the template-free (negative) control demonstrates no amplification curve (no Ct values)
8.	Serology Test	Assays were obtained from SERION ELISA classic kit (Institut Virion/Serion Laboratories, Germany) and used according to the insert of SERION kit. SERION ELISA classic is a qualitative and quantitative immunoassay for detecting human antibodies in serum or plasma with their corresponding antigen. The indirect enzyme immunosorbent assay in this kit was coated with specific antigens of the pathogen of interest. Patient sera are diluted in a rheumatoid factor and then diluted in Sample Diluent (containing phosphate with tween 20 and Bromphenol blue) and incubated in the coated microwells to bind serum antibody to the solid-phase antigen. The microwells are then washed to remove unreacted serum proteins, and enzyme conjugate (anti-human IgA, IgG, or IgM APC_Alkaline phosphatase) is added to label the bound antibody. After further incubation, the microwells are washed to remove unbound APC Conjugate. The pNPP (para-nitrophenyl phosphate) substrate is then added to quantitate the Conjugate-bound p-nitrophenyl phosphate portion. The colorless substrate p-nitrophenyl phosphate is then converted into the colored product p-nitrophenol. The signal intensity of this reaction product is proportional to the concentration of the analyte in the serum antibody. This timed reaction is interrupted with a Stop Solution (sodium hydroxide). Color intensity (Absorbance) is measured at a wavelength of 405nm on a microtiter plate reader or spectrophotometer within 15 minutes of adding the stop solution. Antibody activities are calculated by the SERION evaluation software [15].

Footnote References:

1. Murdoch DR, Morpeth SC, Hammitt LL, et al. Microscopic Analysis and Quality Assessment of Induced Sputum From Children With Pneumonia in the PERCH Study. *Clin Infect Dis an Off Publ Infect Dis Soc Am*. 2017;64(suppl_3):S271-S279. doi:10.1093/cid/cix083
2. Smith AC, Hussey MA. Gram stain protocols. *Am Soc Microbiol*. 2005;1:14.
3. Mardian Y, Menur Naysilla A, Lokida D, et al. Approach to Identifying Causative Pathogens of Community-Acquired Pneumonia in Children Using Culture, Molecular, and Serology Tests. *Front Pediatr*. 2021;9:498. doi:10.3389/fped.2021.629318
4. Murdoch DR, Morpeth SC, Hammitt LL, et al. The Diagnostic Utility of Induced Sputum Microscopy and Culture in Childhood Pneumonia. *Clin Infect Dis an Off Publ Infect Dis Soc Am*. 2017;64(suppl_3):S280-S288. doi:10.1093/cid/cix090
5. Książczyk M, Kuczkowski M, Dudek B, et al. Application of Routine Diagnostic Procedure, VITEK 2 Compact, MALDI-TOF MS, and PCR Assays in Identification Procedure of Bacterial Strain with Ambiguous Phenotype. *Curr Microbiol*. 2016;72(5):570-582. doi:10.1007/s00284-016-0993-0
6. Kirn TJ, Weinstein MP. Update on blood cultures: how to obtain, process, report, and interpret. *Clin Microbiol Infect Off Publ Eur Soc Clin Microbiol Infect Dis*. 2013;19(6):513-520. doi:10.1111/1469-0691.12180
7. O'Brien KL, Baggett HC, Brooks WA, et al. Causes of severe pneumonia requiring hospital admission in children without HIV infection from Africa and Asia: the PERCH multi-country case-control study. *Lancet*. 2019;394(10200):757-779. doi:10.1016/S0140-6736(19)30721-4
8. Beld M, Minnaar R, Weel J, et al. Highly sensitive assay for detection of enterovirus in clinical specimens by reverse transcription-PCR with an armored RNA internal control. *J Clin Microbiol*. 2004;42(7):3059-3064. doi:10.1128/JCM.42.7.3059-3064.2004
9. Jansen RR, Schinkel J, Koekkoek S, et al. Development and evaluation of a four-tube real time multiplex PCR assay covering fourteen respiratory viruses, and comparison to its corresponding single target counterparts. *J Clin Virol Off Publ Pan Am Soc Clin Virol*. 2011;51(3):179-185. doi:10.1016/j.jcv.2011.04.010
10. Reischl U, Lehn N, Sanden GN, Loeffelholz MJ. Real-time PCR assay targeting IS481 of Bordetella pertussis and molecular basis for detecting Bordetella holmesii. *J Clin Microbiol*. 2001;39(5):1963-1966. doi:10.1128/JCM.39.5.1963-1966.2001
11. Pitcher D, Chalker VJ, Sheppard C, George RC, Harrison TG. Real-time detection of Mycoplasma pneumoniae in respiratory samples with an internal processing

For peer review only

control. *J Med Microbiol*. 2006;55(Pt 2):149-155. doi:10.1099/jmm.0.46281-0
12. Greiner O, Day PJR, Altwegg M, Nadal D. Quantitative detection of Moraxella catarrhalis in nasopharyngeal secretions by real-time PCR. *J Clin Microbiol*. 2003;41(4):1386-1390. doi:10.1128/JCM.41.4.1386-1390.2003
13. Meyler KL, Meehan M, Bennett D, Cunney R, Cafferkey M. Development of a diagnostic real-time polymerase chain reaction assay for the detection of invasive Haemophilus influenzae in clinical samples. *Diagn Microbiol Infect Dis*. 2012;74(4):356-362. doi:10.1016/j.diagmicrobio.2012.08.018
14. Gadsby NJ, McHugh MP, Russell CD, et al. Development of two real-time multiplex PCR assays for the detection and quantification of eight key bacterial pathogens in lower respiratory tract infections. *Clin Microbiol Infect Off Publ Eur Soc Clin Microbiol Infect Dis*. 2015;21(8):788.e1-788.e13. doi:10.1016/j.cmi.2015.05.004
15. Gasem MH, Kosasih H, Tjitra E, et al. An observational prospective cohort study of the epidemiology of hospitalized patients with acute febrile illness in Indonesia. *PLoS Negl Trop Dis*. 2020;14(1):e0007927. doi:10.1371/journal.pntd.0007927

Supplementary Table 2. Pathogen distribution by WHO severity classification status and mortality.

Pathogens	WHO Classification System		*p-value*	Mortality Outcome		*p-value*
	Severe (N=89)	Non-severe (N=99)		Died(N=19)	Alive (N=169)	
**Causative Pathogen**						
H. influenzae non-type b	31 (34.8%)	42 (42.4%)	0.286	8 (42.1%)	65 (38.5%)	0.757
RSV	25 (28.1%)	26 (26.3%)	0.778	2 (10.5%)	49 (29.0%)	0.086
K. pneumoniae	15 (16.9%)	28 (28.3%)	0.062	6 (31.6%)	37 (21.9%)	0.388
S. pneumoniae	19 (21.3%)	10 (10.1%)	0.033	1 (5.2%)	28 (16.6%)	0.317
Influenza virus	9 (10.1%)	16 (16.2%)	0.223	3 (15.8%)	22 (13.0%)	0.723
S. aureus	8 (9.0%)	12 (12.1%)	0.487	0 (0.0%)	20 (11.8%)	0.230
PIV	8 (9.0%)	9 (9.1%)	0.981	1 (5.3%)	16 (9.5%)	1.000
hMPV	6 (6.7%)	5 (5.1%)	0.622	1 (5.3%)	10 (5.9%)	1.000
Rhinovirus	7 (7.9%)	3 (3.0%)	0.196	1 (5.3%)	9 (5.3%)	1.000
B. pertussis	4 (4.5%)	3 (3.0%)	0.709	2 (10.5%)	5 (3.0%)	0.150
**Infection Type**						
Bacterial pathogen	17 (19.1%)	31 (31.3%)	0.055	7 (36.8%)	41 (24.3%)	0.268
Viral pathogen	16 (18.0%)	15 (15.2%)	0.602	2 (10.5%)	29 (17.2%)	0.744
Mixed pathogen	38 (42.7%)	38 (38.4%)	0.547	5 (26.3%)	71 (42.0%)	0.186
Unknown pathogen	18 (20.2%)	15 (15.2%)	0.361	5 (26.3%)	28 (16.6%)	0.337

For comparison, Chi-square test for categorical variables was done

For peer review only

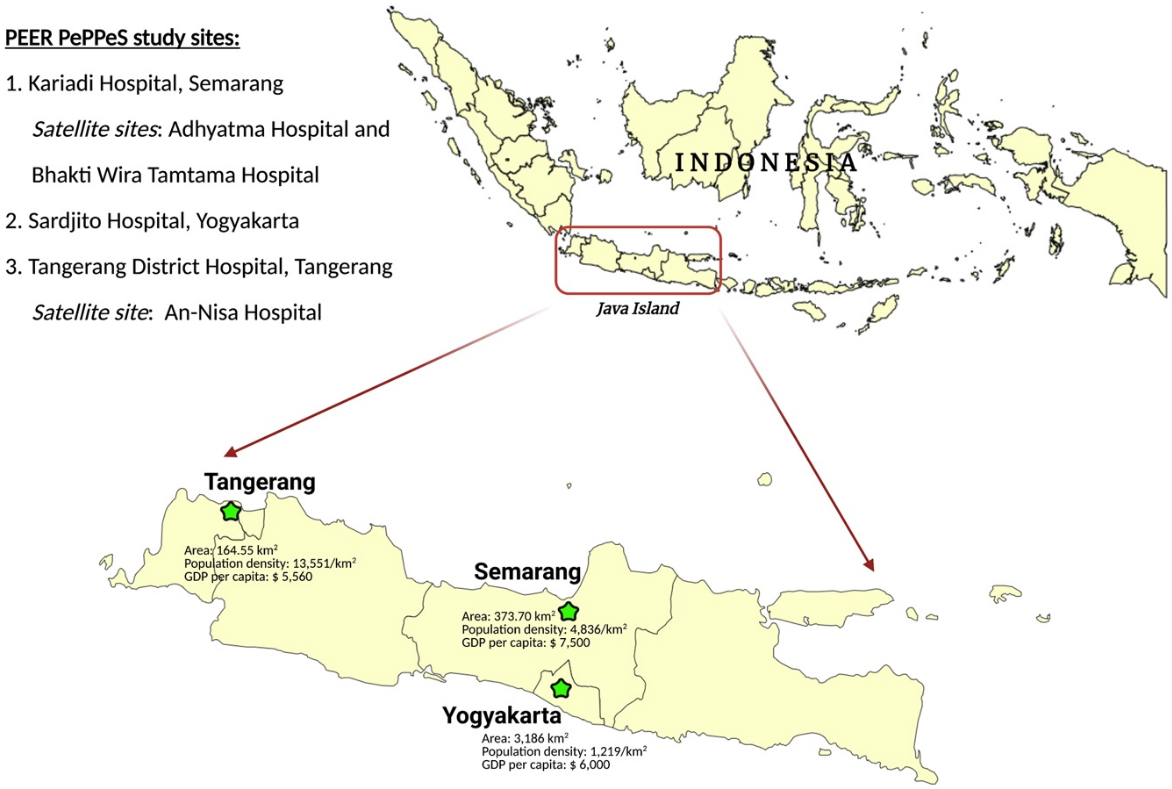

Supplementary Figure 1. PEER-PePPeS Study sites

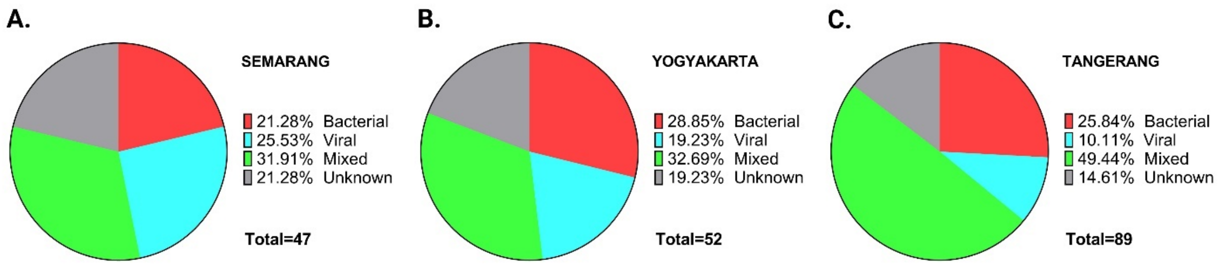

Supplementary Figure 2. Proportion of Identified Pathogen in each Sites. (A) Semarang, (B) Yogyakarta, and (C) Tangerang

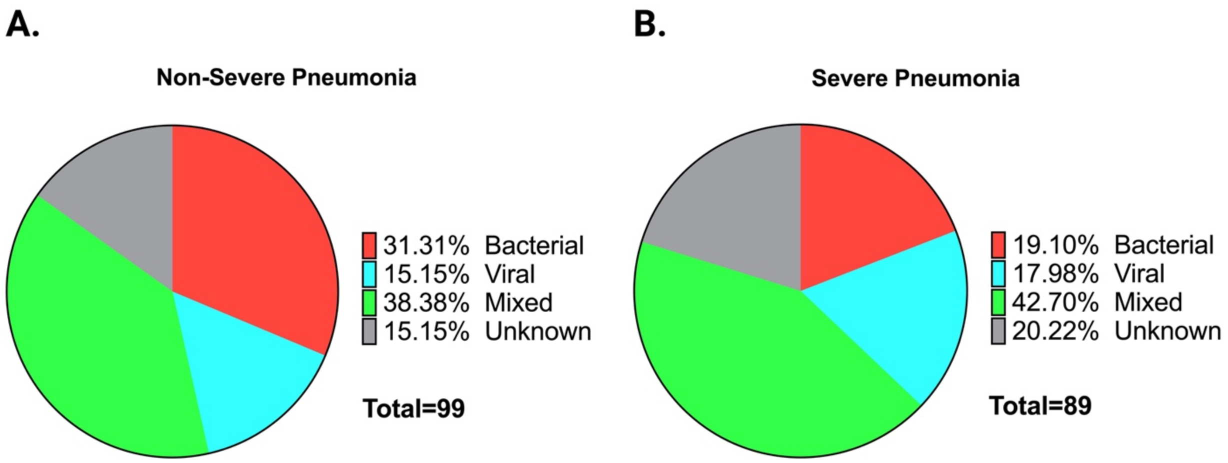

Supplementary Figure 3. Proportion of Identified Pathogen between WHO Severity Status. (A) Non-severe Pneumonia, (B) Severe Pneumonia.

For peer review only

STROBE Statement—Checklist of items that should be included in reports of ***cohort studies***

	Paragraph/ Line number	Recommendation
**Title and abstract**	Page 1-2	(*a*) Indicate the study's design with a commonly used term in the title or the abstract
		(*b*) Provide in the abstract an informative and balanced summary of what was done and what was found
**Introduction**		
Background/rationale	Introduction, paragraph 1, 2, 3, and 4 (Page 3)	Explain the scientific background and rationale for the investigation being reported
Objectives	Introduction, paragraph 5 (Page 4)	State specific objectives, including any prespecified hypotheses
**Methods**		
Study design	Methods, paragraph 1 (Page 4)	Present key elements of study design early in the paper
Setting	Methods, paragraph 1 and 4 (Page 4)	Describe the setting, locations, and relevant dates, including periods of recruitment, exposure, follow-up, and data collection
Participants	Methods, paragraph 4 (Page 5)	(*a*) Give the eligibility criteria, and the sources and methods of selection of participants
Variables	Methods, paragraph 2, 3, and 5 (Page 4-5)	Clearly define all outcomes, exposures, predictors, potential confounders, and effect modifiers. Give diagnostic criteria, if applicable
Data sources/ measurement	Methods, paragraph 5, 6, 7, and 8 (Page 5-6)	For each variable of interest, give sources of data and details of methods of assessment (measurement). Describe comparability of assessment methods if there is more than one group
Bias	Methods, paragraph 9 and 10 (Page 6-7)	Describe any efforts to address potential sources of bias
Study size	Methods, paragraph 1 and 4 (Page 4-5)	Explain how the study size was arrived at
Quantitative variables	Methods, paragraph 9 (Page 6)	Explain how quantitative variables were handled in the analyses. If applicable, describe which groupings were chosen and why
Statistical methods	Methods, paragraph 9 (Page 6)	(*a*) Describe all statistical methods, including those used to control for confounding
		(*b*) Describe any methods used to examine subgroups and interactions
		(*c*) Explain how missing data were addressed
		(*d*) If applicable, describe analytical methods taking account of sampling strategy
		(*e*) Describe any sensitivity analyses
**Results**		
Participants	Results, paragraph 1 (Page 7) and Flow diagram/Fig. 1	(a) Report numbers of individuals at each stage of study—eg numbers potentially eligible, examined for eligibility, confirmed eligible, included in the study, completing follow-up, and analysed
		(b) Give reasons for non-participation at each stage
		(c) Consider use of a flow diagram

For peer review only - http://bmjopen.bmj.com/site/about/guidelines.xhtml

Descriptive data	Results, paragraph 2 and 3. Table 1. (Page 7-8)	(a) Give characteristics of study participants (eg demographic, clinical, social) and information on exposures and potential confounders
		(b) Indicate number of participants with missing data for each variable of interest
Outcome data	Results, paragraph 4 and 5 (Page 8-9)	Report numbers of outcome events or summary measures
Main results	Results, paragraph 5, 7, and 9. Table 2. (Page 9-11). Fig. 2 and Fig. 3	(*a*) Give unadjusted estimates and, if applicable, confounder-adjusted estimates and their precision (eg, 95% confidence interval). Make clear which confounders were adjusted for and why they were included
		(*b*) Report category boundaries when continuous variables were categorized
		(*c*) If relevant, consider translating estimates of relative risk into absolute risk for a meaningful time period
Other analyses	Results, paragraph 6 (Page 9). Results, paragraph 8. (Page 10-11).	Report other analyses done—eg analyses of subgroups and interactions, and sensitivity analyses
**Discussion**		
Key results	Discussion, paragraph 1 (Page 11)	Summarise key results with reference to study objectives
Limitations	Discussion, paragraph 10 (Page 14)	Discuss limitations of the study, taking into account sources of potential bias or imprecision. Discuss both direction and magnitude of any potential bias
Interpretation	Discussion, paragraph 2-9 (Page 11-14)	Give a cautious overall interpretation of results considering objectives, limitations, multiplicity of analyses, results from similar studies, and other relevant evidence
Generalisability	Discussion, paragraph 3-5 (Page 12) Discussion, paragraph 7 (Page 13) Discussion, paragraph 10-11 (Page 14)	Discuss the generalisability (external validity) of the study results
**Other information**		
Funding	Funding statement (Page 15)	Give the source of funding and the role of the funders for the present study and, if applicable, for the original study on which the present article is based

*Give information separately for exposed and unexposed groups.

Note: An Explanation and Elaboration article discusses each checklist item and gives methodological background and published examples of transparent reporting. The STROBE checklist is best used in conjunction with this article (freely available on the Web sites of PLoS Medicine at http://www.plosmedicine.org/, Annals of Internal Medicine at http://www.annals.org/, and Epidemiology at http://www.epidem.com/). Information on the STROBE Initiative is available at www.strobe-statement.org.

For peer review only

For peer review only - http://bmjopen.bmj.com/site/about/guidelines.xhtml

BMJ Open

Epidemiology of community-acquired pneumonia among hospitalized children in Indonesia: a multicenter, prospective study

Journal:	*BMJ Open*
Manuscript ID	bmjopen-2021-057957.R1
Article Type:	Original research
Date Submitted by the Author:	11-Apr-2022

Complete List of Authors:	Lokida, Dewi; Tangerang District General Hospital<br>Farida, Helmia; Rumah Sakit Umum Pusat Dr Kariadi<br>Triasih, Rina; Rumah Sakit Umum Pusat Dr Sardjito<br>Mardian, Yan; Indonesia Research Partnership on Infectious Disease<br>Kosasih, Herman; Indonesia Research Partnership on Infectious Disease<br>Naysilla, Adhella Menur; Indonesia Research Partnership on Infectious Disease<br>Budiman, Arif; Tangerang District General Hospital<br>Hayuningsih, Chakrawati; Tangerang District General Hospital<br>Anam, Moh. Syarofil; Rumah Sakit Umum Pusat Dr Kariadi<br>Wastoro, Dwi; Rumah Sakit Umum Pusat Dr Kariadi<br>Mujahidah, Mujahidah; Rumah Sakit Umum Pusat Dr Sardjito<br>Dipayana, Setya; Rumah Sakit Umum Pusat Dr Kariadi<br>Setyati, Amalia; Rumah Sakit Umum Pusat Dr Sardjito<br>Aman, Abu; Rumah Sakit Umum Pusat Dr Sardjito<br>Lukman, Nurhayati; Indonesia Research Partnership on Infectious Disease<br>Karyana, Muhammad; National Institute of Health Research and Development, Ministry of Health, Republic of Indonesia<br>Kline, Ahnika; National Institute of Allergy and Infectious Diseases<br>Neal, Aaron; National Institute of Allergy and Infectious Diseases<br>Lau, Chuen-Yen; National Cancer Institute<br>Lane, C; National Institute of Allergy and Infectious Diseases

**Primary Subject Heading**:	Infectious diseases
Secondary Subject Heading:	Paediatrics
Keywords:	Epidemiology < TROPICAL MEDICINE, INFECTIOUS DISEASES, PAEDIATRICS

SCHOLARONE™
Manuscripts

I, the Submitting Author has the right to grant and does grant on behalf of all authors of the Work (as defined in the below author licence), an exclusive licence and/or a non-exclusive licence for contributions from authors who are: i) UK Crown employees; ii) where BMJ has agreed a CC-BY licence shall apply, and/or iii) in accordance with the terms applicable for US Federal Government officers or employees acting as part of their official duties; on a worldwide, perpetual, irrevocable, royalty-free basis to BMJ Publishing Group Ltd ("BMJ") its licensees and where the relevant Journal is co-owned by BMJ to the co-owners of the Journal, to publish the Work in this journal and any other BMJ products and to exploit all rights, as set out in our licence.

The Submitting Author accepts and understands that any supply made under these terms is made by BMJ to the Submitting Author unless you are acting as an employee on behalf of your employer or a postgraduate student of an affiliated institution which is paying any applicable article publishing charge ("APC") for Open Access articles. Where the Submitting Author wishes to make the Work available on an Open Access basis (and intends to pay the relevant APC), the terms of reuse of such Open Access shall be governed by a Creative Commons licence – details of these licences and which Creative Commons licence will apply to this Work are set out in our licence referred to above.

Other than as permitted in any relevant BMJ Author's Self Archiving Policies, I confirm this Work has not been accepted for publication elsewhere, is not being considered for publication elsewhere and does not duplicate material already published. I confirm all authors consent to publication of this Work and authorise the granting of this licence.

For peer review only

Epidemiology of Community-acquired pneumonia among hospitalized children in Indonesia: a multicenter, prospective study

AUTHORS:

Dewi Lokida[1], Helmia Farida[2], Rina Triasih[3], Yan Mardian[4*], Herman Kosasih[4], Adhella Menur Naysilla[4], Arif Budiman[1], Chakrawati[1], Moh. Syarofil Anam[2], Dwi Wastoro[2], Mujahidah[2], Setya Dipayana[2], Amalia Setyati[3], Abu Tholib Aman[3], Nurhayati Lukman[4], Muhammad Karyana[5], Ahnika Kline[6], Aaron Neal[6], Chuen-Yen Lau[7], H. Clifford Lane[6]

*Correspondence to Dr. Yan Mardian: ymardian@ina-respond.net

AFFILIATIONS:

1. Tangerang District Hospital, Tangerang, Indonesia
2. Dr. Kariadi Hospital/Diponegoro University, Semarang, Indonesia
3. Dr. Sardjito Hospital/Universitas Gadjah Mada, Yogyakarta, Indonesia,
4. Indonesia Research Partnership on Infectious Disease, Jakarta, Indonesia
5. National Institute of Health Research and Development, Ministry of Health, Republic of Indonesia, Jakarta, Indonesia
6. National Institute of Allergy and Infectious Diseases, National Institutes of Health, Bethesda, MD, United States
7. National Cancer Institute, National Institutes of Health, Bethesda, MD, United States

ABSTRACT:

Objective:

To identify etiologies of childhood community-acquired pneumonia (CAP) based on a comprehensive diagnostic approach.

Design:

"Partnerships for Enhanced Engagement in Research - Pneumonia in Pediatrics (PEER-PePPeS)" study was an observational prospective-cohort study conducted from July 2017 through September 2019.

Setting:

For peer review only

Government referral teaching hospitals and satellite sites in three cities in Indonesia: Semarang, Yogyakarta, and Tangerang.

Participants:

Hospitalized children aged 2–59-months who met the criteria for pneumonia were eligible. Children were excluded if they had been hospitalized for >24 hours; had malignancy or history of malignancy; a history of long-term (>2 months) steroid therapy, or conditions that might interfere with compliance with study procedures.

Main outcome(s) measure(s):

Causative bacterial, viral, or mixed pathogen(s) for the pneumonia were determined using microbiological, molecular, and serologic tests from routinely collected specimens (blood, sputum, and nasopharyngeal swabs). We applied a previously published algorithm (PEER-PePPeS rules) to determine the causative pathogen(s).

Results:

188 subjects were enrolled. Based on our algorithm, 48 (25.5%) had a bacterial infection, 31 (16.5%) had a viral infection, 76 (40.4%) had mixed bacterial and viral infections, and 33 (17.6%) were unable to be classified. The five most common causative pathogens identified were *Haemophilus influenzae* non-type B (N=73, 38.8%), respiratory syncytial virus (RSV) (N=51, 27.1%), *Klebsiella pneumoniae* (N=43, 22.9%), *Streptococcus pneumoniae* (N=29, 15.4%), and Influenza virus (N=25, 13.3%). RSV and Influenza virus diagnoses were highly associated with Indonesia's rainy season (November-March). The polymerase chain reaction (PCR) assays on induced sputum (IS) specimens captured most of the pathogens identified in this study.

Conclusions:

Our study found that *H. influenzae* non-type B and RSV were the most frequently identified pathogens causing hospitalized CAP among Indonesian children aged 2-59 months old. Our study also highlights the importance of PCR for diagnosis and by extension, appropriate use of antimicrobials.

Keywords: Pneumonia; Children; Indonesia; Etiology; Epidemiology

STRENGTHS AND LIMITATIONS OF THIS STUDY

- Prospective multisite study conducted over 27-months
- Used a comprehensive approach (culture, molecular, and paired serology assays) to identify causative pathogens from routinely collected specimens (blood, sputum, and nasopharyngeal swabs)

For peer review only

- The relatively small sample size, geographic limitation to the island of Java and observational design may limit generalizability and causal inference
- We did not collect lung aspirates or pleural fluid specimens, which are preferred for the determination of pneumonia etiology, and did not include healthy control children, limiting ability to estimate the adjusted population attributable fraction (aPaF) for each pathogen.
- Several cases of pneumonia attributed to unknown etiology, which could be due to administration of antibiotics before culture, poor sputum quality, limited bacterial and viral panels used, lack of fungal testing or another factor

INTRODUCTION

Pneumonia is the leading infectious cause of child mortality, with a greater burden in low- and middle-income countries (LMICs).[1] In Indonesia, pneumonia contributed to 15% of childhood deaths and was the second leading cause of death amongst children under five years in 2017.[2] Indonesian practice guidelines are adapted from the World Health Organization (WHO) guidelines, which are based on 1970's – 1990's data showing bacteria such as *Haemophilus influenzae* type b (Hib) and *Streptococcus pneumoniae* caused the majority of fatal pneumonias in children.[3–5] Therefore empiric antibiotics are considered first-line treatment for children with community-acquired pneumonia (CAP).[6–8] Despite evidence that appropriate antibiotics are lifesaving, rational selection of antibiotics for pneumonia is hampered by low adherence to guidelines and scarcity of point-of-care diagnostics.[9–11] Consequently, healthcare providers, particularly those in LMIC, are likely to overtreat non-bacterial pneumonia with antibiotics.[11,12]

Several recent studies of community-acquired pneumonia (CAP) in children have highlighted the role of viral etiologies. Increased recognition of viral etiologies of CAP is likely due to both enhanced molecular diagnostic capacity and wide deployment of Hib and pneumococcal conjugate vaccines [PCV].[13,14] Treatment of non-bacterial pneumonia with antibiotics may engender avoidable antimicrobial resistance. Thus, current data on the etiologies of childhood pneumonia is needed and should be regularly evaluated to inform vaccination policies, empiric management decisions, and targeted treatment.[12]

From a diagnostic standpoint, direct demonstration of organisms by culture (or staining) of lung aspirates has been the standard for determining microbial etiology of CAP.[15] In the current era, many use less-invasive biological specimens (e.g. blood, naso/oropharyngeal secretions, bronchoalveolar lavage, or induced sputum) and employ diverse methods (e.g. culture, PCR, antigen detection, or paired serology) to identify organisms.[16] However, such comprehensive methods are

For peer review only

costly and often require specialized equipment and human resources, limiting feasibility in low-resource settings.[17,18]

Prospective community-based cohort studies that define pathogen(s) causing CAP in Indonesian children are scarce. We conducted a “Partnerships for Enhanced Engagement in Research - Pneumonia in Pediatrics (PEER-PePPeS)” study, which aimed to identify etiologies of childhood CAP using comprehensive diagnostic methods.

METHODS

Study design and study sites

PEER-PePPeS was a multi-site observational cohort study seeking to determine etiologies of CAP amongst children aged 2–59 months in Indonesia. The study was conducted by the Indonesia Research Partnership on Infectious Disease (INA-RESPOND) and enrolled participants initially at three government referral teaching hospitals in three provinces: Kariadi Hospital (Central Java), Sardjito Hospital (Yogyakarta), and Tangerang District Hospital (Banten), as shown in **Supplementary Fig. 1.** Satellite sites located near the primary sites were added during the study to facilitate subject recruitment.

Study Definitions

In this study, pneumonia in children was defined as cough or fever with at least one of the following: shortness of breath (indicated by at least one of the following signs: head bobbing; nasal flaring; chest indrawing or intercostal retracting), tachypnea, grunting, crackles, rhonchi, decreased vesicular breath sounds, bronchial breath sounds or chest x-ray findings consistent with pneumonia. Tachypnea was defined as respiratory rate >50/min for infants 2–12 months and >40/min for children >12–60 months.[19] Abnormal chest x-ray findings consistent with pneumonia were defined as presence of either focal or diffuse infiltrates, a silhouette sign, pleural effusion, or air bronchogram.[20] Chest x-rays were read by the pediatrician.

Based on WHO classification and treatment of childhood pneumonia at health facilities (2014 version), for children 2–59 months of age, severe pneumonia was defined as pneumonia (tachypnea and/or chest indrawing) accompanied by presence of any danger signs, which included the inability to drink, persistent vomiting, convulsions, lethargy or loss of consciousness, stridor in a calm child, or severe malnutrition.[19]

Study Participants

For peer review only

PEER-PePPeS study enrolled children aged 2-59 months, who were hospitalized between July 18th, 2017 until September 25th, 2019, and met the definition for pneumonia. Eligible subjects were enrolled within 24 hours of admission. Children were excluded if they had been hospitalized for >24 hours; had a malignancy or history of malignancy; a history of long term (≥2 months) steroid therapy; or conditions that might interfere with compliance with study procedures (e.g., very ill patients for whom specimens could not be obtained or living outside the area for which follow-up was not practical).

Study Procedures

Demographic and anthropometric data, current signs and symptoms, pregnancy history, vaccination status, breastfeeding history, antibiotic and steroid exposure, family history, medical history, risk factors, hematology profiles, chemistry results, and chest x-ray (per standard of care) were collected at enrollment. Clinical examination (vital signs, general examination, lung auscultation, SpO2); nasopharyngeal (NP) swab for molecular tests; induced sputum (IS) for culture and molecular tests; collection of blood specimens for routine blood count, cultures, molecular tests, serologic tests, C-reactive protein (CRP), and procalcitonin (PCT) were also performed. We prospectively followed subjects daily until hospital discharge; data on vital signs, respiratory signs, intensive care admission, intubation, complications, and treatment were collected. On Day 14, we performed clinical examinations and collected convalescent sera for serology tests; subjects discharged before day 14 returned to clinic for their evaluation. We conducted a telephone interview on Day 30 (±4 days) to assess clinical outcome.

This study used several widely available bacterial and viral respiratory molecular pathogen panels and serologic assays.[21–24] NP and IS specimens were tested with a PCR panel that included twelve-viruses (influenza A, influenza B, adenovirus, enterovirus, bocavirus, respiratory syncytial virus (RSV) A, RSV B, human metapneumovirus (hMPV), rhinovirus, parainfluenza virus (PIV) 1-4, coronavirus OC43, and coronavirus NL63). NP specimens were evaluated by PCR for five bacteria (*Haemophilus influenzae*, *Streptococcus pneumoniae*, *Moraxella catarrhalis*, *Staphylococcus aureus*, and *Klebsiella pneumoniae*), while IS specimens were tested for nine (*Haemophilus influenzae, Streptococcus pneumoniae, Mycoplasma pneumoniae, Chlamydia pneumoniae, Bordetella pertussis, Moraxella catarrhalis, Staphylococcus aureus*, *Klebsiella pneumoniae,* and *Legionella pneumoniae*). Good quality (<10 squamous epithelial cells per low power field[12]) IS specimens underwent culture and gram stain.[25] For whole blood, qPCR was performed for three bacteria (*Haemophilus influenzae*, *Streptococcus pneumoniae*, and *Staphylococcus aureus)*. Serologic testing for seven viruses (influenza A, influenza B, adenovirus, parvovirus B19, echovirus/enterovirus, RSV, parainfluenza virus) and four

For peer review only

bacteria (*Mycoplasma pneumoniae, Chlamydia pneumoniae, Legionella pneumoniae,* and *Bordetella pertussis*) was performed using paired acute-convalescent sera.

Blood culture, IS culture and Gram stain, routine blood count, CRP, PCT, and chest x-ray were performed by the laboratory/radiology department at the hospital site. qPCR and serology assays were performed retrospectively at the INA-RESPOND Reference Laboratory located in Tangerang District Hospital. Details of blood culture, sputum culture, molecular and serology test techniques are shown in **Supplementary Table 1**.

Pathogen Identification

Causative bacterial, viral, or mixed pathogen for the pneumonia was determined based on an algorithm (PEER-PePPeS rules) for interpretation of microbiological, molecular, and serologic test results previously published.[12] In brief, we considered all organisms detected by blood culture, detected by whole blood PCR, or that grew from good quality IS specimen in high quantities with a compatible primary Gram stain as potential causative bacterial pathogens. Bacteria commonly considered contaminants were excluded. For the nasopharynx, potential colonizing bacteria (e.g. *H. influenzae*, *S. pneumoniae*, and *S. aureus*) and potential innocent bystander viruses (e.g. bocavirus, adenovirus, non-SARS human Coronavirus (hCoVs), enterovirus, and rhinovirus) were determined to be causative based on a PCR density cut-off and/or serodiagnosis criteria for paired acute and convalescent sera (seroconversion or a two to four-fold increase in antibody titers in the convalescent specimen).[12]

Data collection and statistical analysis

Data were recorded on paper case report forms and entered in duplicate into OpenClinica (OpenClinica, LLC, MA, USA) by research staff. Categorical variables were summarized using absolute values and percentages, and continuous variables as medians and interquartile ranges (IQRs). Differences in categorical variables were compared using Pearson $\chi 2$ or Fisher's exact test when the expected values in any of the contingency table cells were below 5. Differences in continuous variables were compared using One-way ANOVA or Kruskal-Wallis H-test for data which did not follow the normal distribution based on Levene's test. Statistical analyses were performed using Statistical Package for Social Science (SPSS) software version 23 (IBM Corporation, Armonk, NY, USA). All p-values were two-sided. Level of significance was set at $P < 0.05$.

Patient and public involvement statement

For peer review only

For peer review only - http://bmjopen.bmj.com/site/about/guidelines.xhtml

Patients or the public were not involved in study design or study conduct at any stage from inception to completion and dissemination of this project. Patients who met the eligibility criteria as described above were recruited to this study.

RESULTS

Study Population

Of 444 children who were hospitalized with CAP, 188 (42.3%) were eligible and enrolled in the study. Of 256 screening failures, 31.8% were due to hospitalization >24 hours at the time of screening and 22.1% to circumstances that might interfere with the study procedures. Of the 188 enrolled children, 184 (97.9%) had radiologic evidence of pneumonia. 179 (95.1%) subjects completed the study, including 19 (10.1%) who died. Eight subjects (4.3%) were lost to follow up, and one subject (0.5%) withdrew from the study. The study flow is shown in **Figure 1**.

Demographic and clinical characteristics are presented in **Table 1**. Age, gender, laboratory values, and pneumonia severity by WHO classification were similar across the three study sites. The median age was nine months (IQR, 5 to 20), and 54.7% of subjects were male. The most common comorbid conditions were developmental delay (27.7%), congenital heart disease (26.1%), and severe malnutrition (18.6%), with subjects from Yogyakarta site having the greatest proportion of those comorbidities. The percentage of subjects who had been vaccinated (age-adjusted) against pneumococcus, influenza, Hib-DPT, and measles vaccines were 2.1%, 1.1%, 55.9%, and 75.0%, respectively.

Table 1. Baseline Characteristics of Subjects.

Demographic Characteristics	All (N=188)	Semarang (N=47)	Yogyakarta (N=52)	Tangerang (N=89)	P-value
Age, median (IQR) months	9 (5 – 20)	9 (5.5 – 21)	8 (4 – 13.3)	11 (5-20)	0.442
Gender, Male, (%)	103 (54.7)	29 (61.7)	26 (50)	48 (53.9)	0.493
Household Characteristics, (%):					
• Low Education of Parents*	163 (86.7)	37 (78.7)	43 (82.7)	84 (94.3)	0.019
• Living in a dense neighborhood†	121 (64.4)	19 (40.4)	42 (80.8)	60 (67.4)	<0.001
• Living near waste disposal	70 (37.2)	12 (25.5)	29 (55.8)	29 (32.6)	0.004
• Sick household contact <14 days	109 (58.0)	22 (46.8)	43 (82.7)	44 (49.4)	<0.001
• Exposure to cigarette smoke	120 (63.8)	24 (51.1)	27 (51.9)	69 (77.5)	0.001

For peer review only - http://bmjopen.bmj.com/site/about/guidelines.xhtml

Demographic Characteristics	All (N=188)	Semarang (N=47)	Yogyakarta (N=52)	Tangerang (N=89)	P-value
• Attending daycare	4 (2.1)	2 (4.3)	1 (1.9)	1 (1.1)	0.374
Medical history (%):					
• Premature baby	34 (18.1)	4 (8.5)	16 (30.8)	14 (15.7)	0.012
• Low birth weight	46 (24.4)	12 (25.5)	20 (38.5)	14 (15.7)	0.011
• Developmental delay	52 (27.7)	16 (34.0)	21 (40.4)	15 (16.8)	0.003
• Congenital heart disease	49 (26.1)	16 (34.0)	24 (46.2)	9 (10.1)	<0.001
• Severe malnutrition[‡]	35 (18.6)	10 (21.3)	13 (25.0)	12 (13.5)	0.205
• Neurological disorder	25 (13.3)	5 (10.6)	17 (32.7)	3 (3.4)	<0.001
• Asthma	9 (4.8)	3 (6.4)	1 (1.9)	5 (5.6)	0.563
• HIV disease[§]	2 (1.1)	1 (2.1)	1 (1.9)	0 (0)	0.315
• Tuberculosis (recent/cured)	10 (5.3)	4 (8.5)	2 (3.8)	4 (4.5)	0.588
Immunization history, fully vaccinated for age[||] (%):					
• DPT-Hib	105 (55.9)	30 (63.8)	25 (48.1)	50 (56.2)	0.233
• Influenza	2 (1.1)	0 (0)	2 (3.8)	0 (0)	0.132
• Pneumococcus	4 (2.1)	0 (0)	4 (7.7)	0 (0)	0.009
• Measles	141 (75.0)	38 (80.9)	41 (78.8)	62 (69.7)	0.175
Symptoms and signs (%):					
• Cough	171 (91.0)	40 (85.1)	42 (80.8)	89 (100)	<0.001
• Shortness of breath	174 (92.6)	41 (87.2)	48 (92.3)	85 (95.5)	0.214
• Fever	152 (80.9)	34 (72.3)	35 (67.3)	83 (93.3)	<0.001
• Decreased Consciousness	7 (3.7)	1 (2.1)	1 (1.9)	5 (5.6)	0.612
• Inability to drink	13 (6.9)	4 (8.5)	5 (9.6)	4 (4.5)	0.425
• Diarrhea	36 (19.1)	6 (12.8)	4 (7.7)	26 (29.2)	0.003
• Vomiting	14 (7.4)	4 (8.5)	5 (9.6)	5 (5.6)	0.595
• Seizure	6 (3.2)	1 (2.1)	0 (0)	5 (5.6)	0.203
• Fast breathing	80 (42.6)	15 (31.9)	43 (82.7)	22 (24.7)	<0.001
• Intercostal retraction	171 (91.0)	43 (91.5)	52 (100)	76 (85.4)	0.005
• Rhonchi	168 (89.4)	42 (89.4)	39 (75.0)	87 (97.8)	<0.001
• Wheezing	35 (18.6)	9 (19.1)	10 (19.2)	16 (18.0)	1.000
• Chest indrawing	125 (66.5)	36 (76.6)	43 (82.7)	46 (51.7)	<0.001
• SpO_2 <90% and/or Cyanosis	43 (22.9)	7 (14.9)	17 (32.7)	19 (21.3)	0.098
Leukocyte count, median (IQR) × 10^3/uL	14.0 (10.4 – 18.9)	14.9 (11.1 – 18.8)	12.1 (9.8 – 17.8)	14.0 (10.4 – 19.0)	0.356
Neutrophil-lymphocyte ratio (NLR), median (IQR)	1.4 (0.9 – 2.8)	1.3 (0.9 – 2.6)	1.0 (0.6 – 2.0)	1.9 (1.1 – 3.2)	0.367

For peer review only - http://bmjopen.bmj.com/site/about/guidelines.xhtml

Demographic Characteristics	All (N=188)	Semarang (N=47)	Yogyakarta (N=52)	Tangerang (N=89)	P-value
CRP, median (IQR) mg/L	9.0 (3.6 – 28.0)	11.8 (1.6 – 23.3)	9.0 (4.9 – 21.8)	8.4 (1.5 – 34.1)	0.665
PCT, median (IQR) ng/mL	0.2 (0.1 – 1.7)	0.2 (0.1 – 1.5)	0.2 (0.1 – 1.0)	0.2 (0.1 – 2.6)	0.912
Severe pneumonia (WHO Classification 2014 version) (%)	89 (47.3)	26 (55.3)	26 (50.0)	37 (41.6)	0.281
CXR Findings (%):					
• Pleural effusion	5 (2.7)	1 (2.1)	2 (3.8)	2 (2.2)	0.850
• Interstitial infiltrate	131 (69.7)	26 (55.3)	30 (57.7)	75 (84.3)	<0.001
• Alveolar infiltrate	125 (66.5)	41 (87.2)	44 (84.6)	40 (44.9)	<0.001
Antibiotic administration prior to blood collection for blood culture (%):	150 (79.8)	39 (83.0)	49 (94.2)	62 (69.7)	0.002
• Ampicillin	14 (7.4)	5 (10.6)	9 (17.3)	0 (0)	
• Ampicillin – Gentamicin	64 (34.0)	25 (53.2)	39 (75.0)	0 (0)	
• Cefotaxime	33 (17.5)	0 (0)	0 (0)	33 (37.1)	
• Ceftriaxone	26 (13.8)	0 (0)	0 (0)	26 (29.2)	
• Others[¶]	13 (6.9)	9 (19.1)	1 (1.9)	3 (3.4)	

[*]Low education of parents was defined by highest level of parents' formal education being high school diploma or less; [†]A densely populated neighborhood was defined as >200 people/km^2 or <8 m^2/person in the subject's home; [‡] Severe malnutrition was defined as weight for height below -3 standard deviations from the median of the WHO Child Growth Standards; [§]Subjects were tested for HIV infection if a parent / guardian provided consent and a specimen was available (n=160); [||]Full vaccination was defined as being up to date for age per vaccination schedule at study enrollment; [¶]Other antibiotics include: Amoxicillin, Cefamandole, Ceftazidime, Gentamicin, Cefotaxime – Amikacin, Ceftriaxone – Gentamicin, and Azithromycin – Ampicillin – Gentamicin

The most common symptoms were shortness of breath (92.6%), cough (91.0%), and fever (80.9%). Signs noted during the initial examination included intercostal retraction (91.0%), rhonchi (89.4%), and chest indrawing (66.5%). Of 188 subjects, 172 (91.4%) and 167 (88.8%) had CRP and PCT measured with median values of 9.0 (IQR, 3.6 – 28.0; Ref range ≤5) mg/L and 0.2 (IQR, 0.1 – 1.7; Ref range ≤0.15) ng/mL, respectively. Interstitial infiltrate (69.7%) was the most common radiographic finding. 47.3% of cases were classified as severe pneumonia according to the WHO classification system. All 188 enrolled cases were treated with antibiotic, and 150 of them (79.8%) had received antibiotic prior to blood collection for blood culture, with the combination of ampicillin and gentamicin (34.0%), cefotaxime (17.5%), and ceftriaxone (13.8%) were the three most frequent regimens used.

<u>Detection of Pathogens</u>

Blood and sputum cultures were performed on specimens from 184 (97.9%) and 183 (97.3%) subjects, respectively. A total of 150 (79.8%) children received antibiotics prior to collection of blood for culture. Seventy-five (41.0%) sputum culture isolates were analyzed from specimens meeting the

For peer review only

required quality criteria. A NP or OP swab was obtained from 187 (99.5%) subjects, IS for PCR from 176 (93.6%), whole blood for PCR from 163 (86.7%), and paired acute-convalescent serum specimens for serology from 116 (61.7%) (**Fig. 1**).

The PEER-PePPeS algorithm was used to determine the causative pathogen(s) from those identified by culture, molecular, and serologic assay. Amongst the 188 study participants, 48 (25.5%) had bacterial infection, 31 (16.5%) had viral infection, 76 (40.4%) were of mixed bacterial and viral etiology, and 33 (17.6%) were of unknown etiology (**Fig. 2, Panel A**). Mixed infection, the most common overall etiology, was seen in 38.7% of 2-11 month-olds and in 42.7% of 12-59 month-olds (**Fig 2. Panel B**). Mixed infection was also the predominant etiology across all study sites (**Supplementary Fig. 2**). *H. influenzae* non-type B (N=73), RSV (N=51), *K. pneumoniae* (N=43), *S. pneumoniae* (N=29), Influenza virus (N=25), *S. aureus* (N=20), PIV (N=17), hMPV (N=11), Rhinovirus (N=10), and *B. pertussis* (N=7) were the top ten pathogens identified, more commonly appearing in mixed infection as opposed to as a sole pathogen (**Fig 2. Panel C)**. Influenza virus was significantly higher in the age group 12-59 mo vs 2-11 mo (N=16, 64%, P=0.027), while *S. aureus* was significantly more common in 2-11 mo vs 12-59 mo (N=16, 80%, P=0.024). Though not statistically significant, other pathogens trended toward more frequent detection in age group 2-11 mo (except *B. pertussis*) (**Fig 2. Panel D**). Amongst 76 mixed infection cases, RSV + *H. influenzae* non-type B was the most common co-infection (N=22, 28.9%), followed by RSV + *S. pneumoniae* (N=10, 13.2%), Influenza virus + *H. influenzae* non-type B (N=10, 13.2%), RSV + *K. pneumoniae* (N=9, 11.8%), and Parainfluenza virus + *H. influenzae* non-type B (N=9, 11.8%) (Data not shown).

We observed no difference in pathogen distribution by pneumonia severity based on WHO classification system (**Supplementary Table 2 and Supplementary Fig. 3**). By pathogen, there was no significant difference in distribution between pneumonia severity status or mortality, except for *S. pneumoniae* which was found in significantly more severe cases using the WHO system (*P*=0.033) (**Supplementary Table 2**).

A comparison of positivity rates for each causative pathogen by detection method is shown in **Table 2**. Overall, PCR captured more bacterial pathogens than culture and more viral pathogens than acute-convalescent paired serology. Paired serology was generally helpful in identifying atypical bacteria, such as *C. pneumoniae* and *L. pneumophila*, and upper respiratory tract viruses, such as Rhinovirus and Enterovirus. When comparing blood and IS culture, IS yielded more positive bacterial pathogen results. Similarly, IS PCR captured more pathogens than NP/OP PCR.

Table 2. Causative Pathogens per PEER-PePPeS Rules by Detection Method

For peer review only

For peer review only - http://bmjopen.bmj.com/site/about/guidelines.xhtml

Pathogen	N	Blood culture (% N)	IS culture (% N)	Whole blood PCR (% N)	NP / OP PCR (% N)	IS PCR (% N)	Serology Test (% N)
**Gram-positive cocci bacteria**							
S. pneumoniae	29	1 (3.4%)	3 (10.3%)	0 (0.0%)	21 (72.4%)	28 (96.6%)	
S. aureus	20	0 (0.0%)	7 (35%)	0 (0.0%)	11 (55%)	19 (95%)	
S. mitis	4	0 (0.0%)	4 (100%)				
S. pyogenes	1	0 (0.0%)	1 (100%)				
**Gram-negative cocci bacteria**							
M. catarrhalis	2	0 (0.0%)	2 (100%)		2 (100%)	2 (100%)	
**Gram-negative rods bacteria**							
H. inf non-type b	73	0 (0.0%)	0 (0.0%)	8 (10.9%)	60 (82.2%)	71 (98.6%)	
K. pneumoniae	43	0 (0.0%)	17 (39.5%)		2 (4.7%)	34 (79.1%)	
B. pertussis	7	0 (0.0%)	0 (0.0%)			7 (100%)	
E. coli	5	1 (20%)	4 (80%)				
P. aeruginosa	4	0 (0.0%)	4 (100%)				
A. baumannii	3	0 (0.0%)	3 (100%)				
H. inf type b	2	0 (0.0%)	0 (0.0%)	0 (0.0%)	0 (0.0%)	2 (100%)	
N. meningitidis	1	1 (100%)	1 (100%)				
**Atypical-bacteria**							
C. pneumoniae	5	0 (0.0%)	0 (0.0%)			0 (0.0%)	5 (100%)
M. pneumoniae	5	0 (0.0%)	0 (0.0%)			5 (100%)	1 (20%)
L. pneumophila	1	0 (0.0%)	0 (0.0%)			0 (0.0%)	1 (100%)
**Virus**							
RSV	51				36 (70.6%)	45 (88.2%)	10 (19.6%)
RSV A	15				10 (66.7%)	13 (86.7%)	
RSV B	36				26 (72.2%)	32 (88.8%)	
Influenza virus	25				16 (64%)	22 (88%)	9 (36%)
inf A (H1N1)	7				7 (100%)	7 (100%)	7 (70%)
inf A (H3N2)	3				3 (100%)	3 (100%)	
inf B	14				6 (42.9%)	12 (85.7%)	2 (14.3%)
PIV	17				16 (94.1%)	15 (88.2%)	3 (17.6%)
PIV 1	5				5 (100%)	4 (80%)	3 (17.6%)
PIV 2	0				0 (0.0%)	0 (0.0%)	
PIV 3	11				10 (90.9%)	10 (90.9%)	
PIV 4	1				1 (100%)	1 (100%)	
hMPV	11				5 (45.5%)	10 (90.9%)	
Rhinovirus	10				10 (100%)	6 (60%)	4 (40%)
Enterovirus	5				3 (60%)	3 (60%)	3 (60%)
Bocavirus	3				2 (66.7%)	3 (100%)	
hCoV-NL63	2				2 (100%)	2 (100%)	

Grey-box indicates the assay was not performed

Mortality

Nineteen (10.1%) subjects died during the 30-day study period. Seven were male, and most (17 subjects) were less than 1 year old. Among deceased subjects, median study duration was 12 (IQR, 4 – 17.5) days; 8 were admitted to ICU, and 6 received mechanical ventilation. Twelve died due to

For peer review only - http://bmjopen.bmj.com/site/about/guidelines.xhtml

respiratory failure, three due to sepsis, and three for unknown reasons after discharge (data not shown). Most deaths occurred in the 2-11 mo age group compared with the 12-59 mo age group (78.9% vs. 21.1%, *p*=0.036). Infection of deceased subjects was bacterial-only in 7, viral-only in 2, mixed in 5, and unknown in 5 subjects, with no significant differences between alive and deceased subjects. *H. influenzae* non-type B was the most common pathogen identified in deceased subjects (N=8, with the case fatality rate [CFR] in this study of 11.0%), followed by *K. pneumoniae* (N=6, CFR of 13.9%), Influenza virus (N=3, CFR of 12.0%), *B. pertussis* (N=2, CFR of 28.6%), and RSV (N=2, CFR of 3.9%) (**Supplementary Table 2**). Some deceased subjects had pre-existing health conditions or comorbidities, most common were congenital heart disease (10 subjects), severe malnutrition (7 subjects), and developmental delay (7 subjects). A clinical summary of the fatal cases is shown in **Supplementary Table 3**.

Seasonality

During the 27-month study period, infections caused by RSV and influenza were seen year-round with peak activity occurring during the wet season (November to March) in Indonesia (66.7%, p<0.001; and 64.0%, p=0.012, respectively). However, there was little variation in detection of the most common respiratory bacterial infections by month and season. *H. influenzae* non-type B shows peaks in August (N=12, 16.4%) and March (N=11, 15.1%), while *K. pneumoniae* and *S. pneumoniae* fluctuate at lower levels throughout the year (**Figure 3**).

DISCUSSION:

PEER-PePPeS, a prospective multisite study, addresses a critical knowledge gap about the current epidemiology of pathogens causing CAP in children 2-59 months old in Indonesia. There are no recent prospective Indonesian studies on this topic. Our study found: (1) mixed bacterial and viral infection is the most frequent (N=76, 40.4%) cause of childhood CAP, irrespective of age group and pneumonia severity; (2) bacterial infections were common (66% of cases) with *H. influenzae* non-b type, *K. pneumoniae*, and *S. pneumoniae* as the three most common bacterial etiologies; (3) viral pathogens were also common (57% of PEER-PePPeS subjects), with 16.5% of cases attributed to virus only and RSV and Influenza Virus being the most common viruses identified; and (4) PCR on IS specimens was the most sensitive assay for pathogen identification in this study.

While the finding that mixed bacterial and viral infection is the most identified etiology of childhood CAP is consistent with other studies, clinical significance of the mixed infection remains controversial. It is unclear if both agents act as true pathogens.[22,26] PEER-PePPeS did not

For peer review only - http://bmjopen.bmj.com/site/about/guidelines.xhtml

demonstrate a correlation of mixed infection with pneumonia severity and 30-day mortality. Many deceased cases occurred at a younger age (less than 1 year old), and in the presence of comorbidities, such as congenital heart disease and severe malnutrition, as also shown by previous reports.[27,28] Such factors may need to be considered in the prevention and management of childhood pneumonia to reduce mortality rate.

In recent years, there has been an increased focus on the role of respiratory viruses in childhood pneumonia, partly due to the reduction in bacterial disease associated with the use of conjugate pneumococcal and Hib vaccines and the increased capacity to detect viruses through PCR methods.[21,22,29,30] In PEER-PePPeS, viruses were found in 57% of subjects (virus only + mixed infection), with 16.5% of cases attributed to virus only. Thus, many patients probably received unnecessary antibiotics when they were covered empirically according to current Indonesia guidelines. Improving ability to discriminate between viral and bacterial infections would facilitate optimization of antibiotic administration and counter antimicrobial resistance, a major global health challenge.[31]

RSV and influenza virus are the most commonly detected viruses in this study and may be associated with Indonesia's wet/rainy season, consistent with other reports from tropical regions.[32–34] A high prevalence of RSV was also observed in the GABRIEL and PERCH international case-control studies of childhood pneumonia etiology.[22,30] In terms of mixed infections, we found that RSV + *H. influenzae* non-type B and RSV + *S. pneumoniae* were most common. Since respiratory viruses such as RSV can predispose to secondary bacterial infections, particularly *S. pneumoniae* and *H. influenzae*[35], and conversely bacteria can increase RSV susceptibility[35,36], these co-infections highlight the need for optimizing RSV surveillance, prevention and treatment.

Though influenza virus also increases risk for secondary bacterial infections and is a major cause of childhood morbidity and mortality worldwide, data from developing countries is scarce.[37] In a previous Indonesian study of hospitalized patients with a severe acute respiratory infection (SARI), the prevalence of the influenza virus was 10.6% in children less than 5 years old, and was never diagnosed during hospitalization.[38] PEER-PePPeS confirms the need for improved diagnostic strategies, management optimization, and influenza vaccination in children. Of note, our study was conducted before identification of COVID-19 in Indonesia[39], so did not address the role of COVID-19 in childhood pneumonia.

We also found that 66% of cases were caused by bacterial infection (bacteria only + mixed infection). Overall, *H. influenzae* non-type B was the most common bacteria implicated, followed by *K. pneumoniae* and *S. pneumoniae*. *H. influenzae* non-type B predominance was also observed in a Malaysian study, where 90% of enrolled children were vaccinated against Hib as part of the national

For peer review only - http://bmjopen.bmj.com/site/about/guidelines.xhtml

immunization program.[24] With our moderate (56.4%) Hib vaccine coverage, high incidence of *H. influenzae* non-type B may represent true prevalence or strains not covered by Hib vaccine.[40] This finding agrees with current data that non-typeable *H. influenzae* (NTHi) can cause significant illness, and argues for strengthening the diagnostic laboratory capacity for pediatric specimens.

Identification of *K. pneumoniae* as the second most common bacterial etiology of childhood CAP is consistent with previously reported high carriage rates (~7%) in healthy Indonesian children. Carriage has been related to poor water and food hygiene and may give rise to pneumonia, especially in children with malnutrition.[41] Given *K. pneumoniae's* potential for antibiotic resistance and high virulence of some strains, proactive detection and management strategies should be prioritized.[42]

The relatively low prevalence (15.4%) of *S. pneumoniae* in PEER-PePPes was surprising since carriage rates are high and PCV coverage low in Indonesia.[43] A similar relatively low prevalence of *S. pneumoniae* in childhood CAP was also reported from Malaysia, where PCV coverage is 8.7%[24] and in the PERCH study, reflecting temporal shifts in childhood pneumonia etiologies.[22] As only 4.8% of PEER-PePPeS subjects had received PCV, vaccination alone cannot account for the low *S. pneumoniae* prevalence. It is possible that antibiotic exposure prior to obtaining specimens may reduce median colonization density and lower positive findings yield of *S. pneumoniae* by both culture and PCR.[44] Moreover, our panel did not include *S. pneumoniae* paired serology, which may be useful to increase pneumococcal diagnosis in young children.[45] Nonetheless, *S. pneumoniae* remains an important etiological agent of severe/complicated CAP globally.[46] Our finding that *S. pneumoniae* was significantly associated with severe cases by the WHO classification system supports the need for ongoing surveillance, vaccination and prevention of transmission between adults and children.

Inclusion of several pathogen identification strategies in PEER-PePPes demonstrates the differential utility of assays and specimen types. Our findings highlight the value of molecular assays, especially in culture-negative cases where microorganisms may be nonrecoverable in culture due to prior antibiotics or presence of otherwise hard to culture bacteria.[47,48] PCR is also less laborious and boasts a shorter turn-around-time than conventional culture. PCR can additionally identify genes associated with antibiotic resistance, though conventional culture methods will still be required to confirm phenotypic resistance.[49,50] Even with the limited PCR panels used in our study, molecular assays had greater sensitivity for identification of bacterial pathogens than blood or sputum culture when using the PEER-PePPeS rules. Regardless of the method of detection, targeted treatment can follow empiric treatment once an organism is identified. This facilitates optimization of management while minimizing risk of adverse events and development of antimicrobial resistance due to unnecessary, prolonged use of antibiotics.

For peer review only - http://bmjopen.bmj.com/site/about/guidelines.xhtml

Although sensitive for detection, PCR does not provide information regarding infectiousness or viability. Genome fragments from dead organisms may be detected, often at a low level, even after clinical resolution.[48] Furthermore, negative results may occur due to differential viral kinetics along the respiratory tract. Thus lower respiratory tract specimens, such as IS, should be sought as they originate from the actual site of infection.[12,13] Accordingly, we observed a higher yield from PCR on IS than NP specimens. This finding may be confounded as IS and NP detection panels varied slightly. We also found that the use of paired serologies increased the diagnostic yield and was useful for pathogen confirmation, particularly in the setting of innocent bystander viruses and atypical bacteria.[12]

PEER-PePPeS used a comprehensive approach for pathogen detection to increase diagnostic yield. It also enrolled patients over a 27-month study period, facilitating assessment of seasonality. However, our study has several limitations. The relatively small sample size, geographic limitation to the island of Java and observational design may limit generalizability and causal inference. Second, most subjects (79.8%) received antibiotics before specimens' collection, which is an inherent limitation of this observational study due to early antibiotics administration as per national guideline. To overcome this, our inclusion criteria were to enroll subjects within 24 hours of admission, and specimens were collected as soon as possible to hopefully minimize the effects of antibiotics on culture results. Third, we did not enroll healthy control children, limiting the ability to estimate the adjusted population attributable fraction (aPaF) of each pathogen.[29,30] A healthy control group could have revealed baseline carriage rates, minimizing over-attribution of disease to non-pathogenic organisms.[21,22,29,30] Fourth, we did not collect lung aspirates or pleural fluid specimens, which are superior for determination of pneumonia etiology. [15] Fifth, several subjects had pneumonia of unknown etiology; this may have been due to administration of antibiotics before culture which could reduce sensitivity, poor IS quality, the limited panel of bacterial and viral pathogens tested, lack of fungal testing, or currently unrecognized causes of pediatric pneumonia.

In conclusion, the epidemiology of childhood CAP is constantly evolving in step with social and environmental factors and thus, should be regularly assessed. Our study found that *H. influenzae* non-type B and RSV were the most common pathogens causing hospitalized CAP among Indonesian children aged 2-59 months old, suggesting a changing pathogen profile from the 1970-1990s etiology studies that mainly detected *S. pneumoniae* and *H. influenzae* type B as the most important causes of childhood pneumonia in LMICs.[3–5] PCR on IS demonstrated the best sensitivity for pathogen identification. We recommend incorporating molecular assays for pathogen detection, preferably multiplexed point-of-care assays, into practice guidelines. Improvements in Indonesia's lab infrastructure during the COVID-19 pandemic can be leveraged to facilitate use of molecular assays

For peer review only

for evaluation of childhood CAP. Optimization of pathogen detection to understand changing childhood CAP epidemiology will also inform public policy on prevention and management.

ACKNOWLEDGMENTS:

We are grateful to the parents and children who participated in this study. We thank the site study teams (Isabella Puspa Dewi, Fenny Aztari, Mohammad Rosyid Ridho, Aisyah Pratiwi, Niken Maretasari, Firsty Ilminova, Yanantri Binga Ramsif, Yuli Mawarti, Nenes Prastiwi, and Yanantri Binga Ramsif), INA-RESPOND Reference Laboratory team (Gustiani, Deni Pepy, Wahyu Nawang Wulan, Rizki Amalia Sari, Yuanita Djajady, and Ungke Anton Jaya), US-NIAID and Indonesia NIHRD. We appreciate the collaboration from the hospital Directors at Tangerang District Hospital, An-Nisa Hospital, Kariadi Hospital, Adhyatma Hospital, Bhakti Wira Tamtama Hospital, and Sardjito Hospital. We also would like to thank Aly Diana for her assistance during manuscript submission.

CONTRIBUTORS:

DL, HF, RT, YM, HK, AMN, ATA, CYL, HCL designed and conceptualized the study. DL, HF, RT, AB, C, MSA, DW, M, SD, AS performed clinical assessments and were responsible for data entry. DL, HF, RT, YM, HK, AMN, NL, AK, CYL designed the methodology for pathogen identification. YM, HK, AMN performed data analysis, interpretation and drafted the first manuscript. DL, HK, ATA, MK, AN, CYL, HCL assisted with manuscript writing, analysis, and interpretation of data. All authors contributed to manuscript development, edited for critical content, and have approved the final version.

FUNDING STATEMENT:

This manuscript has been funded in whole or in part with MoH Indonesia, National Academy of Sciences (Sub-Grant Number: 2000007599), and Federal funds from the NIAID, NIH, under contract Nos. HHSN261200800001E and HHSN261201500003I. The content of this publication does not necessarily reflect the views or policies of the Department of Health and Human Services, nor does mention of trade names, commercial products, or organizations imply endorsement by the U.S. Government.

COMPETING INTERESTS:

For peer review only

The authors declare that the research was conducted in the absence of any commercial or financial relationships that could be construed as a potential conflict of interest.

ETHICS APPROVAL:

This study was approved by the Ethical Clearance Committee of Faculty of Medicine, Universitas Indonesia (No. 567/UN2.F1/ETIK/2017). The study was conducted in accordance with the Declaration of Helsinki. Written informed consent was obtained from parents or guardians before enrollment.

DATA AVAILABILITY STATEMENT:

Data are available upon reasonable request. The anonymized data set will be shared following the signing of a data-sharing agreement, with permission of the ethical clearance committee, study authors, and all project partners, exclusively for non-commercial purposes. Please contact the corresponding author with any queries.

REFERENCES:

1 McAllister DA, Liu L, Shi T, *et al.* Global, regional, and national estimates of pneumonia morbidity and mortality in children younger than 5 years between 2000 and 2015: a systematic analysis. *Lancet Glob Heal* 2019;**7**:e47–57. doi:10.1016/S2214-109X(18)30408-X

2 Windi R, Efendi F, Qona'ah A, *et al.* Determinants of acute respiratory infection among children under-five years in Indonesia. *J Pediatr Nurs* 2021;**S0882**-**5963**:00080–4. doi:10.1016/j.pedn.2021.03.010

3 Reñosa MD, Dalglish S, Bärnighausen K, *et al.* Key challenges of health care workers in implementing the integrated management of childhood illnesses (IMCI) program: a scoping review. *Glob Health Action* 2020;**13**:1732669. doi:10.1080/16549716.2020.1732669

4 Shann F. Etiology of severe pneumonia in children in developing countries. *Pediatr Infect Dis* 1986;**5**:247–52. doi:10.1097/00006454-198603000-00017

5 Feikin DR, Hammitt LL, Murdoch DR, *et al.* The Enduring Challenge of Determining Pneumonia Etiology in Children: Considerations for Future Research Priorities. *Clin Infect Dis* 2017;**64**:S188–96. doi:10.1093/cid/cix143

6 Bradley JS, Byington CL, Shah SS, *et al.* The Management of Community-Acquired Pneumonia in Infants and Children Older Than 3 Months of Age: Clinical Practice Guidelines by the Pediatric Infectious Diseases Society and the Infectious Diseases Society of America. *Clin*

For peer review only - http://bmjopen.bmj.com/site/about/guidelines.xhtml

Infect Dis 2011;**53**:e25–76. doi:10.1093/cid/cir531

7 Harris M, Clark J, Coote N, *et al.* British Thoracic Society guidelines for the management of community acquired pneumonia in children: update 2011. *Thorax* 2011;**66**:ii1 LP-ii23. doi:10.1136/thoraxjnl-2011-200598

8 Mathur S, Fuchs A, Bielicki J, *et al.* Antibiotic use for community-acquired pneumonia in neonates and children: WHO evidence review. *Paediatr Int Child Health* 2018;**38**:S66–75. doi:10.1080/20469047.2017.1409455

9 Yusuf Y, Murni I, Setyati A. Irrational use of antibiotics and clinical outcomes in children with pneumonia. *Paediatr Indones* 2018;**57**:211–5. doi:10.14238/pi57.4.2017.211-5

10 Tannous R, Haddad RN, Torbey P-H. Management of Community-Acquired Pneumonia in pediatrics: adherence to clinical guidelines. *Front Pediatr* 2020;**8**:1–6. doi:10.3389/fped.2020.00302

11 Ginsburg AS, Klugman KP. Vaccination to reduce antimicrobial resistance. *Lancet Glob Heal* 2017;**5**:e1176–7. doi:10.1016/S2214-109X(17)30364-9

12 Mardian Y, Menur Naysilla A, Lokida D, *et al.* Approach to Identifying Causative Pathogens of Community-Acquired Pneumonia in Children Using Culture, Molecular, and Serology Tests. Front. Pediatr. 2021;**9**:498. doi:10.3389/fped.2021.629318

13 Mardian Y, Kosasih H, Karyana M, *et al.* Review of Current COVID-19 Diagnostics and Opportunities for Further Development. Front. Med. 2021;**8**:562. doi:10.3389/fmed.2021.615099

14 Levine OS, O'Brien KL, Deloria-Knoll M, *et al.* The Pneumonia Etiology Research for Child Health Project: A 21st Century Childhood Pneumonia Etiology Study. *Clin Infect Dis* 2012;**54**:S93–101. doi:10.1093/cid/cir1052

15 Hammitt LL, Murdoch DR, Scott JAG, *et al.* Specimen collection for the diagnosis of pediatric pneumonia. *Clin Infect Dis* 2012;**54 Suppl 2**:S132–9. doi:10.1093/cid/cir1068

16 Zar HJ, Andronikou S, Nicol MP. Advances in the diagnosis of pneumonia in children. *BMJ* 2017;**358**:j2739. doi:10.1136/bmj.j2739

17 Thomas J, Pociute A, Kevalas R, *et al.* Blood biomarkers differentiating viral versus bacterial pneumonia aetiology: a literature review. *Ital J Pediatr* 2020;**46**:4. doi:10.1186/s13052-020-0770-3

18 Jiao F, Chen J, Wang M, *et al.* Determination of Procalcitonin, C-Reactive Protein and White Blood Cell Levels to Diagnose Community-Acquired Pneumonia (CAP). *Indian J Pediatr* 2019;**86**:763. doi:10.1007/s12098-019-02938-z

19 World Health Organization. *Revised WHO classification and treatment of childhood*

For peer review only

pneumonia at health facilities: evidence summaries. Geneva PP - Geneva: : World Health Organization 2014. https://www.ncbi.nlm.nih.gov/books/NBK264162/

20 Walker CM, Abbott GF, Greene RE, *et al.* Imaging pulmonary infection: classic signs and patterns. *AJR Am J Roentgenol* 2014;**202**:479–92. doi:10.2214/AJR.13.11463

21 Jain S, Williams DJ, Arnold SR, *et al.* Community-Acquired Pneumonia Requiring Hospitalization among U.S. Children. *N Engl J Med* 2015;**372**:835–45. doi:10.1056/NEJMoa1405870

22 Group PER for CH (PERCH) S. Causes of severe pneumonia requiring hospital admission in children without HIV infection from Africa and Asia: the PERCH multi-country case-control study. *Lancet (London, England)* 2019;**394**:757–79. doi:10.1016/S0140-6736(19)30721-4

23 Jansen RR, Schinkel J, Koekkoek S, *et al.* Development and evaluation of a four-tube real time multiplex PCR assay covering fourteen respiratory viruses, and comparison to its corresponding single target counterparts. *J Clin Virol Off Publ Pan Am Soc Clin Virol* 2011;**51**:179–85. doi:10.1016/j.jcv.2011.04.010

24 Nathan AM, Teh CSJ, Jabar KA, *et al.* Bacterial pneumonia and its associated factors in children from a developing country: A prospective cohort study. *PLoS One* 2020;**15**:e0228056. doi:10.1371/journal.pone.0228056

25 Lahti E, Peltola V, Waris M, *et al.* Induced sputum in the diagnosis of childhood community-acquired pneumonia. *Thorax* 2009;**64**:252 LP – 257. doi:10.1136/thx.2008.099051

26 Honkinen M, Lahti E, Österback R, *et al.* Viruses and bacteria in sputum samples of children with community-acquired pneumonia. *Clin Microbiol Infect* 2012;**18**:300–7. doi:https://doi.org/10.1111/j.1469-0691.2011.03603.x

27 Dean P, Florin TA. Factors Associated With Pneumonia Severity in Children: A Systematic Review. *J Pediatric Infect Dis Soc* 2018;**7**:323–34. doi:10.1093/jpids/piy046

28 Chen L, Miao C, Chen Y, *et al.* Age-specific risk factors of severe pneumonia among pediatric patients hospitalized with community-acquired pneumonia. *Ital J Pediatr* 2021;**47**:100. doi:10.1186/s13052-021-01042-3

29 Bhuiyan MU, Snelling TL, West R, *et al.* The contribution of viruses and bacteria to community-acquired pneumonia in vaccinated children: a case-control study. *Thorax* 2019;**74**:261 LP – 269. doi:10.1136/thoraxjnl-2018-212096

30 Bénet T, Sánchez Picot V, Messaoudi M, *et al.* Microorganisms Associated With Pneumonia in Children <5 Years of Age in Developing and Emerging Countries: The GABRIEL Pneumonia Multicenter, Prospective, Case-Control Study. *Clin Infect Dis an Off Publ Infect Dis Soc Am* 2017;**65**:604–12. doi:10.1093/cid/cix378

For peer review only

31 Lucien MAB, Canarie MF, Kilgore PE, *et al.* Antibiotics and antimicrobial resistance in the COVID-19 era: Perspective from resource-limited settings. *Int J Infect Dis* 2021;**104**:250–4. doi:https://doi.org/10.1016/j.ijid.2020.12.087

32 Kosasih H, Klimov A, Xiyan X, *et al.* Surveillance of Influenza in Indonesia, 2003–2007. *Influenza Other Respi Viruses* 2013;**7**:312–20. doi:https://doi.org/10.1111/j.1750-2659.2012.00403.x

33 Suryadevara M, Domachowske JB. Epidemiology and Seasonality of Childhood Respiratory Syncytial Virus Infections in the Tropics. Viruses. 2021;**13**. doi:10.3390/v13040696

34 Hirve S, Newman LP, Paget J, *et al.* Influenza Seasonality in the Tropics and Subtropics – When to Vaccinate? *PLoS One* 2016;**11**:e0153003. doi:10.1371/journal.pone.0153003

35 Griffiths C, Drews SJ, Marchant DJ. Respiratory syncytial virus: Infection, detection, and new options for prevention and treatment. *Clin Microbiol Rev* 2017;**30**:277–319. doi:10.1128/CMR.00010-16

36 Gulraiz F, Bellinghausen C, Bruggeman CA, *et al.* Haemophilus influenzae increases the susceptibility and inflammatory response of airway epithelial cells to viral infections. *FASEB J Off Publ Fed Am Soc Exp Biol* 2015;**29**:849–58. doi:10.1096/fj.14-254359

37 Dananché C, Sánchez Picot V, Bénet T, *et al.* Burden of Influenza in Less Than 5-Year-Old Children Admitted to Hospital with Pneumonia in Developing and Emerging Countries: A Descriptive, Multicenter Study. *Am J Trop Med Hyg* 2018;**98**:1805–10. doi:10.4269/ajtmh.17-0494

38 Aman AT, Wibawa T, Kosasih H, *et al.* Etiologies of severe acute respiratory infection (SARI) and misdiagnosis of influenza in Indonesia, 2013-2016. *Influenza Other Respi Viruses* 2021;**15**:34–44. doi:https://doi.org/10.1111/irv.12781

39 Setiawaty V, Kosasih H, Mardian Y, *et al.* The Identification of First COVID-19 Cluster in Indonesia. *Am J Trop Med Hyg*;**103**:2339–42. doi:10.4269/ajtmh.20-0554

40 Agrawal A, Murphy TF. Haemophilus influenzae infections in the H. influenzae type b conjugate vaccine era. *J Clin Microbiol* 2011;**49**:3728–32. doi:10.1128/JCM.05476-11

41 Helmia F, A. SJ, Hussein GM, *et al.* Nasopharyngeal Carriage of Klebsiella pneumoniae and Other Gram-Negative Bacilli in Pneumonia-Prone Age Groups in Semarang, Indonesia. *J Clin Microbiol* 2013;**51**:1614–6. doi:10.1128/JCM.00589-13

42 Pranavathiyani G, Prava J, Rajeev AC, *et al.* Novel Target Exploration from Hypothetical Proteins of Klebsiella pneumoniae MGH 78578 Reveals a Protein Involved in Host-Pathogen Interaction. *Front Cell Infect Microbiol* 2020;**10**:109. doi:10.3389/fcimb.2020.00109

43 Farida H, Severin JA, Gasem MH, *et al.* Nasopharyngeal Carriage of Streptococcus pneumonia

For peer review only

in Pneumonia-Prone Age Groups in Semarang, Java Island, Indonesia. *PLoS One* 2014;**9**:e87431. doi:10.1371/journal.pone.0087431

44 Driscoll AJ, Deloria Knoll M, Hammitt LL, *et al.* The Effect of Antibiotic Exposure and Specimen Volume on the Detection of Bacterial Pathogens in Children With Pneumonia. *Clin Infect Dis* 2017;**64**:S368–77. doi:10.1093/cid/cix101

45 Andrade DC, Borges IC, Vilas-Boas AL, *et al.* Infection by Streptococcus pneumoniae in children with or without radiologically confirmed pneumonia. *J Pediatr (Rio J)* 2018;**94**:23–30. doi:10.1016/j.jped.2017.03.004

46 Principi N, Esposito S. Management of severe community-acquired pneumonia of children in developing and developed countries. *Thorax* 2011;**66**:815–22. doi:10.1136/thx.2010.142604

47 Lleo MM, Ghidini V, Tafi MC, *et al.* Detecting the presence of bacterial DNA by PCR can be useful in diagnosing culture-negative cases of infection, especially in patients with suspected infection and antibiotic therapy. *FEMS Microbiol Lett* 2014;**354**:153–60. doi:10.1111/1574-6968.12422

48 Torres A, Lee N, Cilloniz C, *et al.* Laboratory diagnosis of pneumonia in the molecular age. *Eur Respir J* 2016;**48**:1764 LP – 1778. doi:10.1183/13993003.01144-2016

49 Morozumi M, Chiba N, Ubukata K, *et al.* Antibiotic susceptibility in relation to genotype of Streptococcus pneumoniae, Haemophilus influenzae, and Mycoplasma pneumoniae responsible for community-acquired pneumonia in children. *J Infect Chemother* 2013;**19**:432–40. doi:10.1007/s10156-012-0500-x

50 Aydemir O, Aydemir Y, Ozdemir M. The role of multiplex PCR test in identification of bacterial pathogens in lower respiratory tract infections. *Pakistan J Med Sci* 2014;**30**:1011–6. doi:10.12669/pjms.305.5098

FIGURE CAPTIONS:

Figure 1. Subject screening, enrolment, and monitoring flowchart. CAP, community-acquired pneumonia; RR, respiratory rate; CXR, chest X-Ray; CRP, C-reactive protein; PCT, procalcitonin; NP, nasopharyngeal; IS, induced sputum; PCR, polymerase chain reaction.

Figure 2. Pathogen Distribution. (A) Overall proportion of identified viral/bacterial/mixed pathogen, (B) Viral/bacterial/mixed pathogens by age group, (C) Pattern of detection of the ten most identified pathogens, (D) Distribution of ten most identified pathogens by age group. *P<0.05

For peer review only

Figure 3. Distribution of the (A) monthly count and (B) seasonal pattern of infection caused by *H. influenzae* non-type B, RSV, *K. pneumoniae*, *S. pneumoniae*, and Influenza virus during a 27-month study period.

SUPPLEMENTARY INFORMATION:

Details of Microbiological, Molecular and Serologic Methods, **Supplementary Table 1**

Pathogen distribution by WHO severity classification status and mortality, **Supplementary Table 2**

Summary of fatal cases, **Supplementary Table 3.**

PEER-PePPeS Study sites, **Supplementary Figure 1**

Proportion of Identified Pathogen in each Sites, **Supplementary Figure 2**

Proportion of Identified Pathogen between WHO Severity Status, **Supplementary Figure 3**

For peer review only

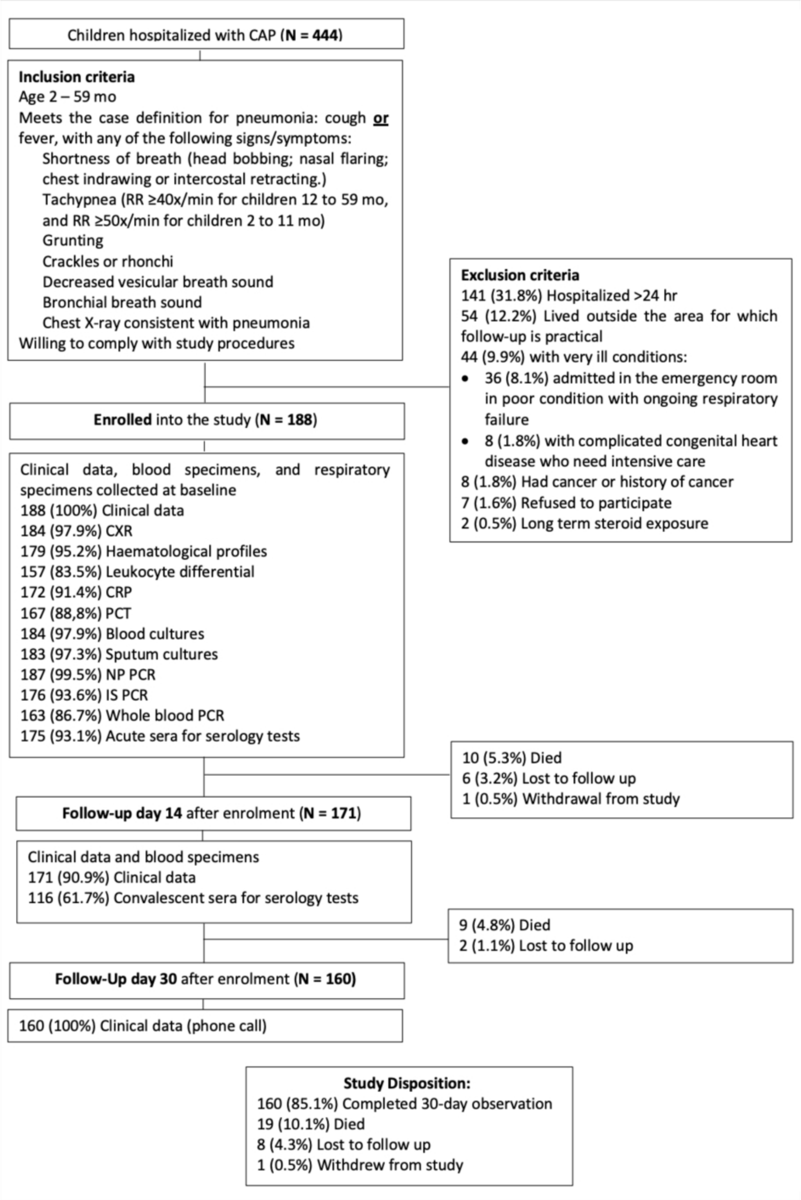

Figure 1. Subject screening, enrolment, and monitoring flowchart. CAP, community-acquired pneumonia; RR, respiratory rate; CXR, chest X-Ray; CRP, C-reactive protein; PCT, procalcitonin; NP, nasopharyngeal; IS, induced sputum; PCR, polymerase chain reaction.

254x381mm (300 x 300 DPI)

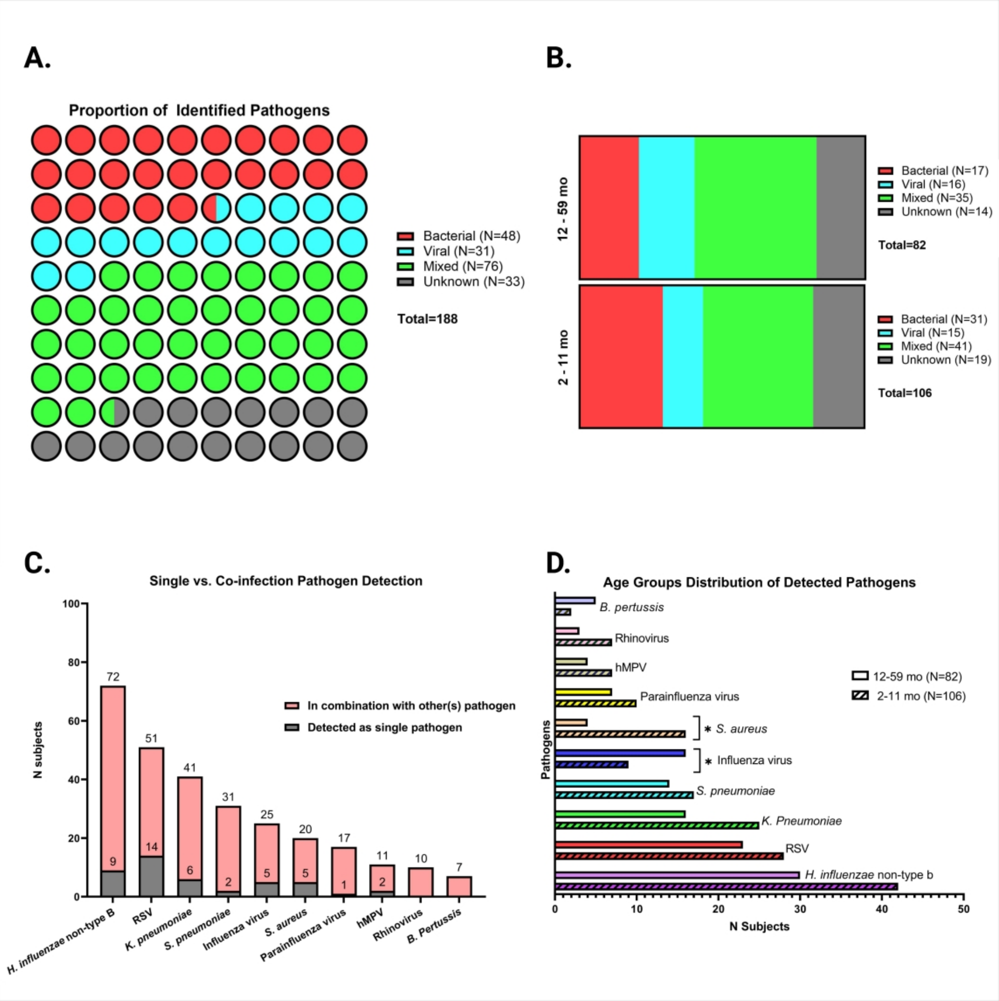

Figure 2. Pathogen Distribution. (A) Overall proportion of identified viral/bacterial/mixed pathogen, (B) Viral/bacterial/mixed pathogens by age group, (C) Pattern of detection of the ten most identified pathogens, (D) Distribution of ten most identified pathogens by age group. *P<0.05

381x381mm (300 x 300 DPI)

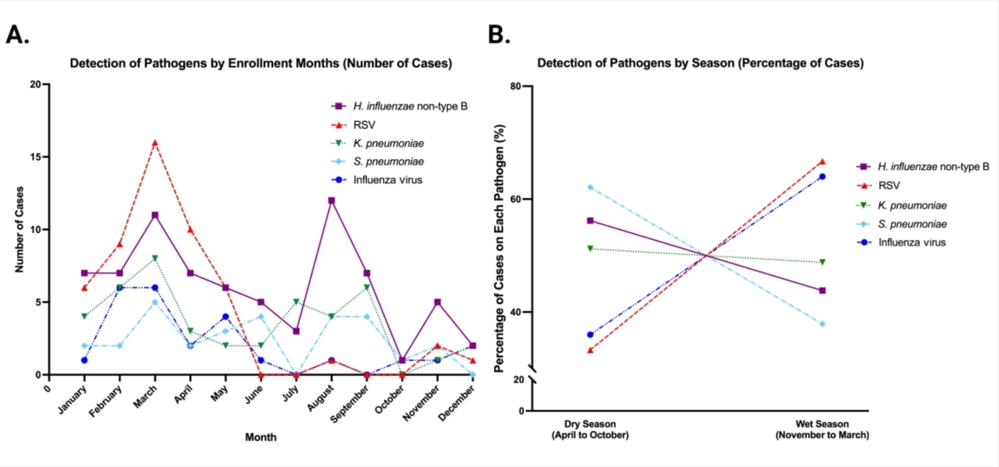

Figure 3. Distribution of the (A) monthly count and (B) seasonal pattern of infection caused by H. influenzae non-type B, RSV, K. pneumoniae, S. pneumoniae, and Influenza virus during a 27-month study period.

381x177mm (300 x 300 DPI)

SUPPLEMENTARY MATERIALS.

Supplementary Table 1. Microbiological, Molecular and Serologic Methods

No.	Assays	Procedures
1.	Gram stain	Gram-stained smears were obtained from the most purulent portion of each induced sputum specimen. The good quality specimen was defined as <10 squamous epithelium per low-power field (magnification, 100×) [1]. The procedure of the Gram stain required four basic steps that include applied a primary stain (crystal violet) to a heat-fixed smear, followed by the addition of a mordant (Gram's Iodine), rapid decolorization with alcohol, acetone, or a mixture of alcohol and acetone and lastly, counterstained with safranin [2]. The Gram-stained smears interpreted as follows: Gram-positive lancet-shaped diplococci (GPDC) suggest *Streptococcus pneumoniae*; Gram-positive diplococci (GPDC) or cocci in chains suggest *Streptococcus pyogenes*; Gram-positive cocci in clusters (GPC-cluster) suggest *Staphylococcus aureus*; Gram-negative coccobacilli (GNCB) suggest *Hemophilus influenzae*, *Bordetella pertussis* or *Acinetobacter baumannii*; Gram-negative diploccoci (GNDC) suggest *Moraxella catarrhalis*; large Gram-negative rods (GNR-large) suggest *Klebsiella pneumoniae* or *Escherichia coli*; and small Gram-negative rods (GNR-small) suggest *Pseudomonas aeruginosa* [3].
2.	Induced Sputum Culture	The most purulent portion of induced sputum was inoculated onto sheep blood, chocolate, and MacConkey agars, streaked out using a standard 4-quadrant streaking method, and incubated at 35°C for 48 hours. Cultures were examined at 24 hours and 48 hours, and predominant bacteria were identified and quantified according to the farthest quadrant with visible colonies (first quadrant, scanty; second quadrant, 1+; third quadrant, 2+; fourth quadrant, 3+) [4]. Then, the predominant bacteria isolates were inoculated into the appropriate VITEK identification strip using the VITEK® 2 COMPACT (BioMérieux, Germany). Briefly, a bacterial suspension was adjusted to a McFarland standard of 0.50 in a solution of 0.45 % sodium chloride using DensiLameter. The time between preparation of the solution and filling of the card was always less than 1 h. Analysis was done using the identification card and automatically read every 15 min. Bacteria identification and antibiotic susceptibility testing results were analyzed using the VITEK 2 software according to the manufacturer's instructions [5].
3.	Blood Culture	Up to 2 mL of blood samples (2 bottle sets) were collected and sent to the site laboratory with standardized procedures. Blood cultures were incubated for at least 5 days, unless positive, using automated systems (BacT/ALERT in Tangerang Hospital; BACTEC at other sites) [6]. Organisms were identified according to standard microbiological methods as described in induced sputum culture section. The following organisms were considered to be contaminants when identified in blood cultures: Coagulase-negative *staphylococci*, *Micrococcus* spp., *Propionibacterium* spp., Alpha-hemolytic streptococci (except

For peer review only

No.	Assays	Procedures
		pneumococcus, *Streptococcus anginosus*, and *Streptococcus mitis*), *Enterococcus* spp., *Corynebacterium* spp. (diphtheroids), *Bacillus* spp. (except *Bacillus anthracis*), *Pseudomonas* spp. (except *Pseudomonas aeruginosa*), *Stomatococcus, Aeroccocus, Neiserria subflava, Veillonella* spp., other environmental non-fermenting Gram negative rods, and *Candida* spp [7].
4.	Viral RNA Extraction	Viral RNA was extracted from viral transport media (VTM) containing respiratory swab as well as sputum, using the QIAamp Viral RNA Mini Kit (Qiagen, Hilden, Germany) according to the manufacturer's protocol. Briefly, 140 µl of VTM or sputum coat was lysed in 560 of carrier RNA-containing AVL buffer, followed by the binding of viral RNA to the QIAamp membrane. Contaminants were removed from viral RNA in two separate washing steps using two different wash buffers, AW1 and AW2. Viral RNA was eluted in 60 µl of AVE buffer and kept in -80° C if not directly used [8,9].
5.	Bacterial DNA Extraction	Bacterial DNA was extracted from viral transport media (VTM) containing respiratory swab as well as sputum, using the QIAamp® DNA Mini Kit (Qiagen, Hilden, Germany) according to the manufacturer's protocol. Briefly, 20 µl of QIAGEN Protease and 200 µl of VTM or sputum coat was lysed in 200 of AL buffer, followed binding of DNA to the QIAamp membrane. Contaminants were removed from DNA in two separate washing steps using two different wash buffers, AW1 and AW2. Bacterial DNA was eluted in 200 µl of AE buffer and kept in -80° C if not directly used
6.	qPCR for Respiratory Viruses	The realtime PCR for respiratory virus detection was done followed the protocol of Beld et al., 2004 and Jansen et al., 2011. Positive control is a synthetic plasmid carrying the nucleotide sequence of the detection target. Primers, probes, and positive controls were synthesized and purified by an outside vendor (Integrated DNA Technologies, Iowa, US). Realtime PCR was done using the TaqManTM Fast Virus 1-Step Master Mix (Thermo Fisher Scientific; Cat#: 4444432) in an Applied Biosystems 7500 Fast Realtime PCR System (Thermo Fisher Scientific, MA, US). The reaction mixture composition was 1X TaqManTM Fast Virus 1-Step Master Mix, 0.5 µM of each primer, 0.25 µM probe, and 4 µl RNA, in a total 20 µl volume. The cycle condition was 50° reverse transcription for 5 minutes, 95° C initial denaturation for 20 seconds, followed by 45 cycles of denaturation (95° C, 3 seconds) and annealing/elongation (55° C, 30 seconds). Realtime PCR works correctly when the positive control demonstrates the amplification curve and the template-free (negative) control demonstrates no amplification curve (no Ct values) [8,9].
7.	qPCR for Respiratory Bacteria	In real-time PCR (qPCR) a portion of bacterial DNA genome specific to the pathogen(s) of interest is amplified using a specific pair of primers and probes for each bacteria, that were selected from the available literature [10–14]. A detector (TaqMan® probe) is used in the reaction. Mastermix is prepared in a 1.5-ml tube for total reaction. qPCR assays were carried out in a total volume of 20 µL, comprising 10 µL of TaqMan® Fast Universal PCR Master Mix, 1.4 µL of nuclease-free water (Promega), 3.6 µL of oligonucleotide mixtures, and 4 µL of

For peer review only

No.	Assays	Procedures
		DNA extract. The cycle condition was 95° C initial denaturation for 20 seconds, followed by 45 cycles of denaturation (95° C, 3 seconds) and annealing/elongation (58° C, 30 seconds). Realtime PCR works correctly when the positive control demonstrates the amplification curve and the template-free (negative) control demonstrates no amplification curve (no Ct values)
8.	Serology Test	Assays were obtained from SERION ELISA classic kit (Institut Virion/Serion Laboratories, Germany) and used according to the insert of SERION kit. SERION ELISA classic is a qualitative and quantitative immunoassay for detecting human antibodies in serum or plasma with their corresponding antigen. The indirect enzyme immunosorbent assay in this kit was coated with specific antigens of the pathogen of interest. Patient sera are diluted in a rheumatoid factor and then diluted in Sample Diluent (containing phosphate with tween 20 and Bromphenol blue) and incubated in the coated microwells to bind serum antibody to the solid-phase antigen. The microwells are then washed to remove unreacted serum proteins, and enzyme conjugate (anti-human IgA, IgG, or IgM APC_Alkaline phosphatase) is added to label the bound antibody. After further incubation, the microwells are washed to remove unbound APC Conjugate. The pNPP (para-nitrophenyl phosphate) substrate is then added to quantitate the Conjugate-bound p-nitrophenyl phosphate portion. The colorless substrate p-nitrophenyl phosphate is then converted into the colored product p-nitrophenol. The signal intensity of this reaction product is proportional to the concentration of the analyte in the serum antibody. This timed reaction is interrupted with a Stop Solution (sodium hydroxide). Color intensity (Absorbance) is measured at a wavelength of 405nm on a microtiter plate reader or spectrophotometer within 15 minutes of adding the stop solution. Antibody activities are calculated by the SERION evaluation software [15].

Footnote References:

1. Murdoch DR, Morpeth SC, Hammitt LL, et al. Microscopic Analysis and Quality Assessment of Induced Sputum From Children With Pneumonia in the PERCH Study. *Clin Infect Dis an Off Publ Infect Dis Soc Am*. 2017;64(suppl_3):S271-S279. doi:10.1093/cid/cix083
2. Smith AC, Hussey MA. Gram stain protocols. *Am Soc Microbiol*. 2005;1:14.
3. Mardian Y, Menur Naysilla A, Lokida D, et al. Approach to Identifying Causative Pathogens of Community-Acquired Pneumonia in Children Using Culture, Molecular, and Serology Tests. *Front Pediatr*. 2021;9:498. doi:10.3389/fped.2021.629318
4. Murdoch DR, Morpeth SC, Hammitt LL, et al. The Diagnostic Utility of Induced Sputum Microscopy and Culture in Childhood Pneumonia. *Clin Infect Dis an Off Publ Infect Dis Soc Am*. 2017;64(suppl_3):S280-S288. doi:10.1093/cid/cix090
5. Książczyk M, Kuczkowski M, Dudek B, et al. Application of Routine Diagnostic Procedure, VITEK 2 Compact, MALDI-TOF MS, and PCR Assays in Identification Procedure of Bacterial Strain with Ambiguous Phenotype. *Curr Microbiol*. 2016;72(5):570-582. doi:10.1007/s00284-016-0993-0
6. Kirn TJ, Weinstein MP. Update on blood cultures: how to obtain, process, report, and interpret. *Clin Microbiol Infect Off Publ Eur Soc Clin Microbiol Infect Dis*. 2013;19(6):513-520. doi:10.1111/1469-0691.12180
7. O'Brien KL, Baggett HC, Brooks WA, et al. Causes of severe pneumonia requiring hospital admission in children without HIV infection from Africa and Asia: the PERCH multi-country case-control study. *Lancet*. 2019;394(10200):757-779. doi:10.1016/S0140-6736(19)30721-4
8. Beld M, Minnaar R, Weel J, et al. Highly sensitive assay for detection of enterovirus in clinical specimens by reverse transcription-PCR with an armored RNA internal control. *J Clin Microbiol*. 2004;42(7):3059-3064. doi:10.1128/JCM.42.7.3059-3064.2004
9. Jansen RR, Schinkel J, Koekkoek S, et al. Development and evaluation of a four-tube real time multiplex PCR assay covering fourteen respiratory viruses, and comparison to its corresponding single target counterparts. *J Clin Virol Off Publ Pan Am Soc Clin Virol*. 2011;51(3):179-185. doi:10.1016/j.jcv.2011.04.010

For peer review only

10. Reischl U, Lehn N, Sanden GN, Loeffelholz MJ. Real-time PCR assay targeting IS481 of Bordetella pertussis and molecular basis for detecting Bordetella holmesii. *J Clin Microbiol*. 2001;39(5):1963-1966. doi:10.1128/JCM.39.5.1963-1966.2001

11. Pitcher D, Chalker VJ, Sheppard C, George RC, Harrison TG. Real-time detection of Mycoplasma pneumoniae in respiratory samples with an internal processing control. *J Med Microbiol*. 2006;55(Pt 2):149-155. doi:10.1099/jmm.0.46281-0

12. Greiner O, Day PJR, Altwegg M, Nadal D. Quantitative detection of Moraxella catarrhalis in nasopharyngeal secretions by real-time PCR. *J Clin Microbiol*. 2003;41(4):1386-1390. doi:10.1128/JCM.41.4.1386-1390.2003

13. Meyler KL, Meehan M, Bennett D, Cunney R, Cafferkey M. Development of a diagnostic real-time polymerase chain reaction assay for the detection of invasive Haemophilus influenzae in clinical samples. *Diagn Microbiol Infect Dis*. 2012;74(4):356-362. doi:10.1016/j.diagmicrobio.2012.08.018

14. Gadsby NJ, McHugh MP, Russell CD, et al. Development of two real-time multiplex PCR assays for the detection and quantification of eight key bacterial pathogens in lower respiratory tract infections. *Clin Microbiol Infect Off Publ Eur Soc Clin Microbiol Infect Dis*. 2015;21(8):788.e1-788.e13. doi:10.1016/j.cmi.2015.05.004

15. Gasem MH, Kosasih H, Tjitra E, et al. An observational prospective cohort study of the epidemiology of hospitalized patients with acute febrile illness in Indonesia. *PLoS Negl Trop Dis*. 2020;14(1):e0007927. doi:10.1371/journal.pntd.0007927

Supplementary Table 2. Pathogen distribution by WHO severity classification status and mortality.

Pathogens	WHO Classification System		*p-value*	Mortality Outcome		*p-value*
	Severe (N=89)	Non-severe (N=99)		Died (N=19)	Alive (N=169)	
**Causative Pathogen**						
H. influenzae non-type b	31 (34.8%)	42 (42.4%)	0.286	8 (42.1%)	65 (38.5%)	0.757
RSV	25 (28.1%)	26 (26.3%)	0.778	2 (10.5%)	49 (29.0%)	0.086
K. pneumoniae	15 (16.9%)	28 (28.3%)	0.062	6 (31.6%)	37 (21.9%)	0.388
S. pneumoniae	19 (21.3%)	10 (10.1%)	0.033	1 (5.2%)	28 (16.6%)	0.317
Influenza virus	9 (10.1%)	16 (16.2%)	0.223	3 (15.8%)	22 (13.0%)	0.723
S. aureus	8 (9.0%)	12 (12.1%)	0.487	0 (0.0%)	20 (11.8%)	0.230
PIV	8 (9.0%)	9 (9.1%)	0.981	1 (5.3%)	16 (9.5%)	1.000
hMPV	6 (6.7%)	5 (5.1%)	0.622	1 (5.3%)	10 (5.9%)	1.000
Rhinovirus	7 (7.9%)	3 (3.0%)	0.196	1 (5.3%)	9 (5.3%)	1.000
B. pertussis	4 (4.5%)	3 (3.0%)	0.709	2 (10.5%)	5 (3.0%)	0.150
**Infection Type**						
Bacterial pathogen	17 (19.1%)	31 (31.3%)	0.055	7 (36.8%)	41 (24.3%)	0.268
Viral pathogen	16 (18.0%)	15 (15.2%)	0.602	2 (10.5%)	29 (17.2%)	0.744
Mixed pathogen	38 (42.7%)	38 (38.4%)	0.547	5 (26.3%)	71 (42.0%)	0.186
Unknown pathogen	18 (20.2%)	15 (15.2%)	0.361	5 (26.3%)	28 (16.6%)	0.337

Differences in categorical variables were compared using Pearson χ2 or Fisher's exact test when the expected values in any of the contingency table cells were below 5.

Supplementary Table 3. Summary of fatal cases.

Case, Site, Gender (Age, mo)	Medical History	Signs and Symptoms (SS), Vital Signs (VS), Laboratory parameter (Lab) at admission	CXR	Causative Pathogen	ABX during Hospitalization	Hospitalization status	Cause of Death
#01, SMG, Male (4)	Recurrent pneumonia, congenital heart disease, severe malnutrition	• **SS:** Cough, fever, dyspnea, chest indrawing, intercostal retraction, rhonchi<br>• **VS:** 38°C, RR 44x/min, SpO_2 97%<br>• **Lab:** Hb 9.6 g/dL, WBC 24.1 $\times 10^9$/L, PLT 350 $\times 10^9$/L, NLR 4.63, CRP 25.70 mg/L, PCT 2.41 ng/mL	Alveolar infiltrate	Rhinovirus, *H. influenzae non-type b*	Ampicillin, Gentamicin, Ceftriaxon, Cefoperazone Sulbactam	On mechanical ventilator<br>ICU admission (25 days)<br>Died on Day-26	Cardiopulmonary failure<br>Sepsis

For peer review only

Case, Site, Gender (Age, mo)	Medical History	Signs and Symptoms (SS), Vital Signs (VS), Laboratory parameter (Lab) at admission	CXR	Causative Pathogen	ABX during Hospitalization	Hospitalization status	Cause of Death
#02, SMG, Female (23)	Recurrent pneumonia, congenital heart disease, incomplete NIP (DPT-Hib), malnutrition, developmental delay	• **SS:** Cough, fever, dyspnea, chest indrawing, intercostal retraction, rhonchi<br>• **VS:** 37.5°C, RR 56x/min, SpO_2 95%<br>• **Lab:** Hb 10.6 g/dL, WBC 14.1 ×10^9/L, PLT 405 ×10^9/L, NLR 9.63, CRP 14.90 mg/L, PCT 0.37 ng/mL	Alveolar and interstitial infiltrates	Influenza A (H1N1)	Ampicillin, Gentamicin, Metronidazole, Ceftriaxon, Meropenem	On mechanical ventilator<br>ICU admission (9 days)<br>Died on Day 21	Cardiopulmonary failure
#03, SMG, Female (11)	Low birth weight, congenital heart disease, incomplete NIP (Measles), severe malnutrition, developmental delay	• **SS:** Cough, fever, dyspnea, diarrhea, nasal flaring, chest indrawing, intercostal retraction, rhonchi<br>• **VS:** 38.3°C, RR 45x/min, SpO_2 96%<br>• **Lab:** Hb 8.1 g/dL, WBC 15.9 ×10^9/L, PLT 677 ×10^9/L, NLR 1.87	Alveolar and interstitial infiltrates	Influenza A (H3N2), *B. pertussis, H. influenzae non-type b, K. pneumoniae*	Ampicillin, Gentamicin, Azithromycin	On nasal cannula<br>Died on day 19	Cardiopulmonary failure
#04, SMG, Male (45)	Recurrent pneumonia, frontometaphyseal dysplasia syndrome, epilepsy, developmental delay	• **SS:** Cough, fever, dyspnea, nasal flaring, intercostal retraction, rhonchi, wheezing<br>• **VS:** 36.7°C, RR 40x/min, SpO_2 99%<br>• **Lab:** Hb 13.7 g/dL, WBC 11.3 ×10^9/L, PLT 277 ×10^9/L, NLR 0.98, CRP 0.10 mg/L, PCT 0.05 ng/mL	Alveolar infiltrate	Unknown	Ampicillin, Gentamicin	On Nasal cannula<br>Died on day 2	Respiratory failure
#05, SMG, Male (5)	Premature birth, low birth weight, recurrent pneumonia, congenital heart disease, incomplete NIP (DPT-Hib)	• **SS:** Cough, dyspnea, nasal flaring, chest indrawing, intercostal retraction,<br>• **VS:** 36.8°C, RR 30x/min, SpO_2 98%<br>• **Lab:** Hb 10.9 g/dL, WBC 12.4 ×10^9/L, PLT 396 ×10^9/L, CRP 0.80 mg/L, PCT 128 ng/mL	Alveolar infiltrate	*K. pneumoniae*	Ampicillin, Gentamicin	On Simple mask<br>ICU admission (1 day)<br>Died on day 6	Cardiopulmonary failure
#06, SMG, Female (3)	Recurrent pneumonia, incomplete NIP (DPT-Hib), malnutrition	• **SS:** Cough, dyspnea, chest indrawing, intercostal retraction, rhonchi<br>• **VS:** 36.7°C, RR 42x/min, SpO_2 99%<br>• **Lab:** Hb 8.2 g/dL, WBC 16 ×10^9/L, PLT 499 ×10^9/L, ANC 6.7, NLR 0.76, CRP 13.10 mg/L, PCT 0.28 ng/mL	Alveolar infiltrate	Unknown	Ampicillin, Gentamicin, Vancomycin, Metronidazol, Meropenem	On mechanical ventilator<br>ICU admission (7 days)<br>Died on day 18	Septic shock, respiratory failure
#07, YGY, Female (10)	Congenital heart disease, incomplete NIP (DPT-Hib, and Measles), severe malnutrition, developmental delay	• **SS:** Cough, fever, dyspnea, head bobbing, chest indrawing, intercostal retraction, rhonchi<br>• **VS:** 39.0 °C, RR 64x/min, SpO_2 96%<br>• **Lab:** Hb 10.1 g/dL, WBC 12.1 ×10^9/L, PLT 415 ×10^9/L, ANC 6.0, NLR 1.15, CRP 4.90 mg/L, PCT 0.11 ng/mL	Alveolar infiltrate	hMPV, RSV A	Ampicillin, Gentamicin, Ceftriaxone, Cotrimoxazole	On mechanical ventilator/ ICU admission (13 days)<br>Died on day 17	Sepsis, Pulmonary crisis due to pulmonary hypertension
#08, YGY, Female (3)	Low birth weight, congenital heart disease, incomplete NIP (DPT-Hib), severe malnutrition	• **SS:** Cough, fever, dyspnea, chest indrawing, intercostal retraction, rhonchi<br>• **VS:** 37.2 °C, RR 49x/min, SpO_2 56%<br>• **Lab:** Hb 9.7 g/dL, WBC 11.3 ×10^9/L, PLT 115 ×10^9/L, ANC 7.0, NLR 1.92	Alveolar and interstitial infiltrates	Unknown	Ampicillin, Ceftriaxone	On nasal cannula<br>Hospital discharge on day 10<br>Died on day 29 (outside hospitalization)	Acute Respiratory Distress Syndrome
#09, YGY, Female (5)	Congenital heart disease, incomplete NIP (DPT-Hib), severe malnutrition	• **SS:** Cough, dyspnea, inability to drink, nasal flaring, chest indrawing, intercostal retraction, rhonchi<br>• **VS:** 37.0 °C, RR 60x/min, SpO_2 96%<br>• **Lab:** Hb 10.3 g/dL, WBC 26.9 ×10^9/L, PLT 788 ×10^9/L, ANC 18.5, NLR 2.97	Alveolar and interstitial infiltrates	*H. influenzae non-type b, K. pneumoniae*	Ampicillin, Gentamicin	On nasal cannula<br>Died on day 15	Aspiration, mucous hypersecretion
#10, YGY, Male (6)	Recurrent pneumonia, congenital heart disease, tuberculosis, incomplete NIP (DPT-Hib)	• **SS:** Cough, fever, dyspnea, nasal flaring, chest indrawing, intercostal retraction, rhonchi, wheezing<br>• **VS:** 37.3 °C, RR 50x/min, SpO_2 89%<br>• **Lab:** Hb 11.6 g/dL, WBC 13.3 ×10^9/L, PLT 189 ×10^9/L, ANC 3.7, NLR 0.48, CRP 4.90 mg/L, PCT 0.08 ng/mL	Alveolar and interstitial infiltrates, pleural effusion	*K. pneumoniae*	Ampicillin, Gentamicin, Ceftriaxone	On non-rebreather mask<br>Died on day 4	Septic shock
#11, TRG, Female (5)	Premature birth, developmental delay	• **SS:** Cough, fever, dyspnea, nasal flaring, rhonchi, wheezing	Alveolar infiltrate	*A. baumannii* (MDR)	Cefotaxime	On Nasal cannula<br>Hospital discharge on day 7	Unknown death

For peer review only

Case, Site, Gender (Age, mo)	Medical History	Signs and Symptoms (SS), Vital Signs (VS), Laboratory parameter (Lab) at admission	CXR	Causative Pathogen	ABX during Hospitalization	Hospitalization status	Cause of Death
		• **VS:** 37.5 °C, RR 48x/min, SpO_2 31%<br>• **Lab:** Hb 8.5 g/dL, WBC 12.1 ×10^9/L, PLT 208 ×10^9/L, ANC 8.6, NLR 3.23, CRP 0.91 mg/L, PCT 0.74 ng/mL				Died on day 17 (outside hospitalization)	
#12, TRG, Female (2)	Incomplete NIP (DPT-Hib)	• **SS:** Cough, fever, dyspnea, diarrhea, skin rash, intercostal retraction, rhonchi, wheezing<br>• **VS:** 37.6 °C, RR 63x/min, SpO_2 93%<br>• **Lab:** Hb 10.5 g/dL, WBC 13.6 ×10^9/L, PLT 289 ×10^9/L, ANC 10.2, NLR 3.95, CRP 175.30 mg/L, PCT 0.7 ng/mL	Alveolar and interstitial infiltrates	Unknown	Ceftriaxone, Ceftazidime, Azithromycin	On Nasal cannula<br>Died on day 8	Sepsis
#13, TRG, Female (2)	Incomplete NIP (DPT-Hib)	• **SS:** Cough, fever, dyspnea, nasal flaring, chest indrawing, intercostal retraction, rhonchi<br>• **VS:** 36 °C, RR 45x/min, SpO_2 96%<br>• **Lab:** Hb 7.8 g/dL, WBC 21.2 ×10^9/L, PLT 563 ×10^9/L, ANC 16.5, NLR 3.9, CRP 280.30 mg/L, PCT 0.09 ng/mL	Alveolar and interstitial infiltrates, pleural effusion	Influenza B, *S. mitis* (MDR)	Ceftazidime	On non-rebreather mask ICU admission (3 days)<br>Died on day 3	Respiratory Failure
#14, TRG, Female (2)	Congenital heart disease, incomplete NIP (DPT-Hib), severe malnutrition	• **SS:** Cough, fever, dyspnea, nasal flaring, chest indrawing, intercostal retraction, rhonchi, wheezing<br>• **VS:** 37 °C, RR 60x/min, SpO_2 76%<br>• **Lab:** Hb 9.5 g/dL, WBC 17.2 ×10^9/L, PLT 296 ×10^9/L, ANC 8.8, NLR 1.42, CRP 0.70 mg/L, PCT 0.02 ng/mL	Interstitial infiltrate	Unknown	Cefotaxime	On Simple mask<br>Died on day 2	Respiratory Failure
#15, TRG, Male (9)	Incomplete NIP (Measles)	• **SS:** Cough, fever, dyspnea, nasal flaring, chest indrawing, intercostal retraction, rhonchi<br>• **VS:** 37 °C, RR 30x/min, SpO_2 89%<br>• **Lab:** Hb 6.4 g/dL, WBC 25.7 ×10^9/L, PLT 801 ×10^9/L, ANC 18.5, NLR 3.43, CRP 33.35 mg/L, PCT 0.34 ng/mL	Interstitial infiltrate	*H. influenzae non-type b*	Cefotaxime, Ceftriaxone, Meropenem	On mechanical ventilator<br>ICU admission (8 days)<br>Died on day 12	Meningoencephalitis, Respiratory Failure
#16, TRG, Female (4)	Premature birth, low birth weight, congenital heart disease, incomplete NIP (DPT-Hib)	• **SS:** Cough, fever, dyspnea, diarrhea, chest indrawing, intercostal retraction, rhonchi<br>• **VS:** 38 °C, RR 32x/min, SpO_2 85%<br>• **Lab:** Hb 9.2 g/dL, WBC 16.8 ×10^9/L, PLT 224 ×10^9/L, ANC 9.4, NLR 2.24, CRP 2.46 mg/L, PCT 2.24 ng/mL	Alveolar and interstitial infiltrates,	*H. influenzae non-type b*, *K. pneumoniae*	Cefotaxime, Gentamicin, Ceftriaxone	On nasal cannula<br>Died on day 11	Unknown death
#17, TRG, Female (20)	Developmental delay, incomplete NIP (DPT-Hib)	• **SS:** Cough, fever, dyspnea, chest indrawing, intercostal retraction, rhonchi<br>• **VS:** 36.3°C, RR 40x/min, SpO_2 75%<br>• **Lab:** Hb 7.0 g/dL, WBC 15.2 ×10^9/L, PLT 668 ×10^9/L, ANC 9.7, NLR 2.13, CRP 55.10 mg/L	Alveolar and interstitial infiltrates, pleural effusion	*H. influenzae non-type b*, *K. pneumoniae*	Cefotaxime, Gentamicin, Ceftriaxone	On mechanical ventilator<br>ICU admission (3 days)<br>Died on day 8	Septic shock, Cardiopulmonary failure
#18, TRG, Male (4)	Low birth weight, developmental delay, recurrent pneumonia, incomplete NIP (DPT-Hib), severe malnutrition	• **SS:** Cough, fever, dyspnea, nasal flaring, chest indrawing, intercostal retraction, rhonchi<br>• **VS:** 36.7 °C, RR 30x/min, SpO_2 92%<br>• **Lab:** Hb 11.6 g/dL, WBC 20.5 ×10^9/L, PLT 433 ×10^9/L, ANC 11.9, NLR 2.52, CRP 16.80 mg/L, PCT 20.1 ng/mL	Alveolar and interstitial infiltrates,	PIV 3, *H. influenzae non-type b*, *S. pneumoniae*	Ceftazidime	On non-rebreather mask<br>Died on day 3	Respiratory failure
#19, TRG, Male (15)	Incomplete NIP (DPT-Hib and Measles)	• **SS:** Cough, fever, dyspnea, rhonchi<br>• **VS:** 37.8 °C, RR 52x/min, SpO_2 80%<br>• **Lab:** Hb 9.4 g/dL, WBC 23.6 ×10^9/L, PLT 786 ×10^9/L, CRP 3.30 mg/L, PCT 0.07 ng/mL	Interstitial infiltrate	RSV B, *B. pertussis*, *H. influenzae non-type b*	Cefotaxime	On nasal cannula<br>Hospital discharged on day 5<br>Died on day 20 (outside hospitalization)	Unknown death

Abbreviation. SMG: Semarang site; YGY: Yogyakarta site; TGR: Tangerang site; NIP: mandatory National Immunization Program; DPT-Hib: a combined vaccine of adsorbed diphtheria, tetanus toxoids, acellular pertussis and of *Haemophilus influenzae* type b conjugate vaccines; CXR: chest X-ray; ABX: Antibiotics; RSV: Respiratory Syncytial Virus; hMPV: Human Metapneumovirus; PIV: Parainfluenza Virus; MDR: Multiple drug resistance.

For peer review only

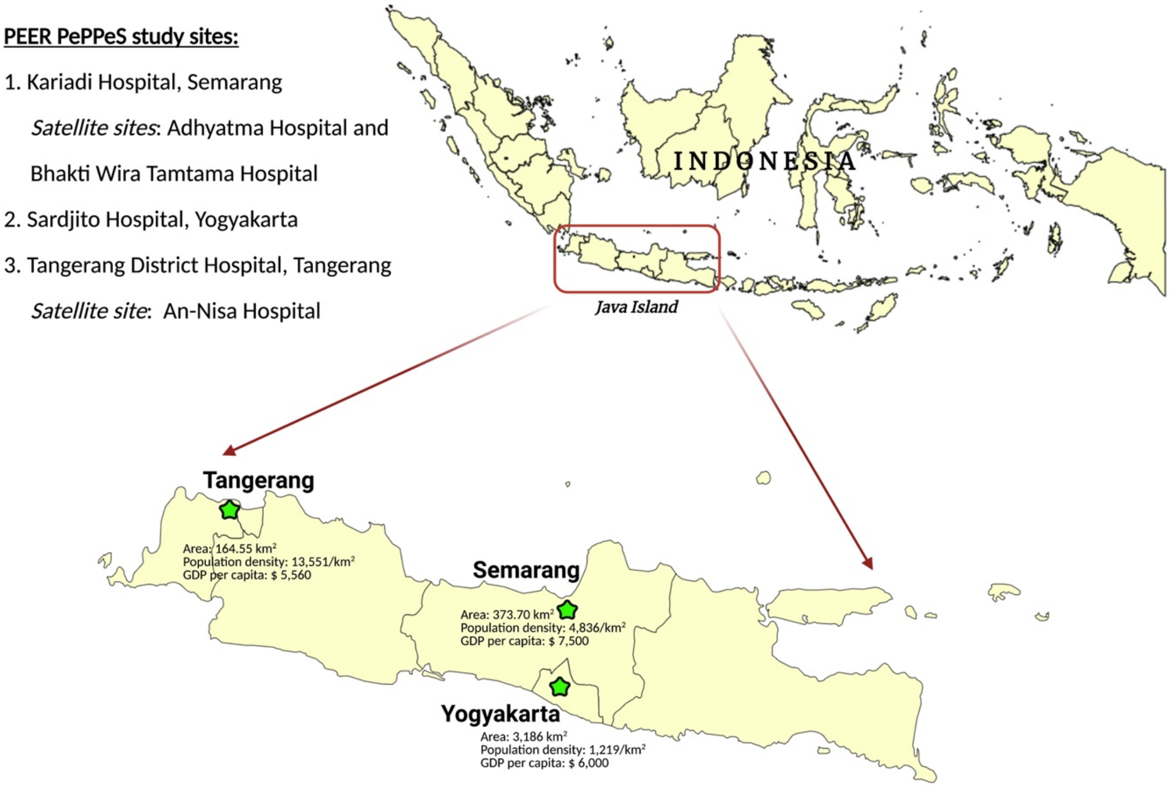

Supplementary Figure 1. PEER-PePPeS Study sites

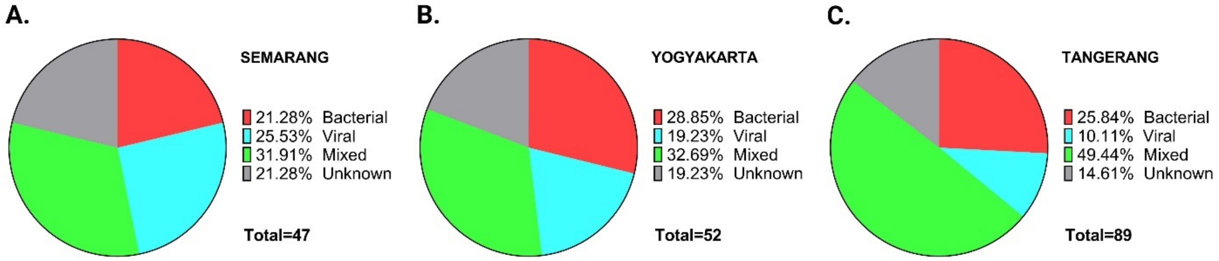

Supplementary Figure 2. Proportion of Identified Pathogen in each Sites. (A) Semarang, (B) Yogyakarta, and (C) Tangerang

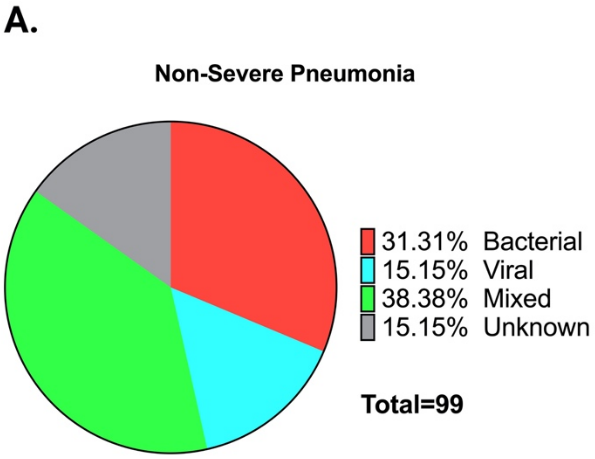

Supplementary Figure 3. Proportion of Identified Pathogen between WHO Severity Status. (A) Non-severe Pneumonia, (B) Severe Pneumonia.

STROBE Statement—Checklist of items that should be included in reports of ***cohort studies***

	Paragraph/ Line number	Recommendation
**Title and abstract**	Page 1-2	(*a*) Indicate the study's design with a commonly used term in the title or the abstract
		(*b*) Provide in the abstract an informative and balanced summary of what was done and what was found
**Introduction**		
Background/rationale	Introduction, paragraph 1-3 (Page 3-4)	Explain the scientific background and rationale for the investigation being reported
Objectives	Introduction, paragraph 4 (Page 4)	State specific objectives, including any prespecified hypotheses
**Methods**		
Study design	Methods, paragraph 1 (Page 4)	Present key elements of study design early in the paper
Setting	Methods, paragraph 1 and 4 (Page 4-5)	Describe the setting, locations, and relevant dates, including periods of recruitment, exposure, follow-up, and data collection
Participants	Methods, paragraph 4 (Page 5)	(*a*) Give the eligibility criteria, and the sources and methods of selection of participants
Variables	Methods, paragraph 2, 3, and 5 (Page 4-5)	Clearly define all outcomes, exposures, predictors, potential confounders, and effect modifiers. Give diagnostic criteria, if applicable
Data sources/ measurement	Methods, paragraph 5, 6, 7, and 8 (Page 5-6)	For each variable of interest, give sources of data and details of methods of assessment (measurement). Describe comparability of assessment methods if there is more than one group
Bias	Methods, paragraph 9 and 10 (Page 6-7)	Describe any efforts to address potential sources of bias
Study size	Methods, paragraph 1 and 4 (Page 4-5)	Explain how the study size was arrived at
Quantitative variables	Methods, paragraph 9 (Page 6)	Explain how quantitative variables were handled in the analyses. If applicable, describe which groupings were chosen and why
Statistical methods	Methods, paragraph 9 (Page 6)	(*a*) Describe all statistical methods, including those used to control for confounding
		(*b*) Describe any methods used to examine subgroups and interactions
		(*c*) Explain how missing data were addressed
		(*d*) If applicable, describe analytical methods taking account of sampling strategy
		(*e*) Describe any sensitivity analyses
**Results**		
Participants	Results, paragraph 1 (Page 7) and Flow diagram/Fig. 1	(a) Report numbers of individuals at each stage of study—eg numbers potentially eligible, examined for eligibility, confirmed eligible, included in the study, completing follow-up, and analysed
		(b) Give reasons for non-participation at each stage
		(c) Consider use of a flow diagram
Descriptive data	Results, paragraph 2 and 3. Table 1. (Page 7-9)	(a) Give characteristics of study participants (eg demographic, clinical, social) and information on exposures and potential confounders
		(b) Indicate number of participants with missing data for each variable of interest
Outcome data	Results, paragraph 4 and 5 (Page 9-10)	Report numbers of outcome events or summary measures
Main results		(*a*) Give unadjusted estimates and, if applicable, confounder-adjusted estimates and their precision (eg, 95% confidence

For peer review only - http://bmjopen.bmj.com/site/about/guidelines.xhtml

	Results, paragraph 5, 7, and 9. Table 2. (Page 10-12). Fig. 2 and Fig. 3	interval). Make clear which confounders were adjusted for and why they were included
		(*b*) Report category boundaries when continuous variables were categorized
		(*c*) If relevant, consider translating estimates of relative risk into absolute risk for a meaningful time period
Other analyses	Results, paragraph 6 (Page 10). Results, paragraph 8. (Page 11-12).	Report other analyses done—eg analyses of subgroups and interactions, and sensitivity analyses
**Discussion**		
Key results	Discussion, paragraph 1 (Page 12)	Summarise key results with reference to study objectives
Limitations	Discussion, paragraph 11 (Page 15)	Discuss limitations of the study, taking into account sources of potential bias or imprecision. Discuss both direction and magnitude of any potential bias
Interpretation	Discussion, paragraph 2-10 (Page 12-15)	Give a cautious overall interpretation of results considering objectives, limitations, multiplicity of analyses, results from similar studies, and other relevant evidence
Generalisability	Discussion, paragraph 3-8 (Page 13-14) Discussion, paragraph 12 (Page 15-16)	Discuss the generalisability (external validity) of the study results
**Other information**		
Funding	Funding statement (Page 16)	Give the source of funding and the role of the funders for the present study and, if applicable, for the original study on which the present article is based

*Give information separately for exposed and unexposed groups.

Note: An Explanation and Elaboration article discusses each checklist item and gives methodological background and published examples of transparent reporting. The STROBE checklist is best used in conjunction with this article (freely available on the Web sites of PLoS Medicine at http://www.plosmedicine.org/, Annals of Internal Medicine at http://www.annals.org/, and Epidemiology at http://www.epidem.com/). Information on the STROBE Initiative is available at www.strobe-statement.org.

For peer review only

BMJ Open

Epidemiology of community-acquired pneumonia among hospitalized children in Indonesia: a multicenter, prospective study

Journal:	*BMJ Open*
Manuscript ID	bmjopen-2021-057957.R2
Article Type:	Original research
Date Submitted by the Author:	25-May-2022

Complete List of Authors:	Lokida, Dewi; Tangerang District General Hospital<br>Farida, Helmia; Rumah Sakit Umum Pusat Dr Kariadi<br>Triasih, Rina; Rumah Sakit Umum Pusat Dr Sardjito<br>Mardian, Yan; Indonesia Research Partnership on Infectious Disease<br>Kosasih, Herman; Indonesia Research Partnership on Infectious Disease<br>Naysilla, Adhella Menur; Indonesia Research Partnership on Infectious Disease<br>Budiman, Arif; Tangerang District General Hospital<br>Hayuningsih, Chakrawati; Tangerang District General Hospital<br>Anam, Moh. Syarofil; Rumah Sakit Umum Pusat Dr Kariadi<br>Wastoro, Dwi; Rumah Sakit Umum Pusat Dr Kariadi<br>Mujahidah, Mujahidah; Rumah Sakit Umum Pusat Dr Sardjito<br>Dipayana, Setya; Rumah Sakit Umum Pusat Dr Kariadi<br>Setyati, Amalia; Rumah Sakit Umum Pusat Dr Sardjito<br>Aman, Abu; Rumah Sakit Umum Pusat Dr Sardjito<br>Lukman, Nurhayati; Indonesia Research Partnership on Infectious Disease<br>Karyana, Muhammad; National Institute of Health Research and Development, Ministry of Health, Republic of Indonesia<br>Kline, Ahnika; National Institute of Allergy and Infectious Diseases<br>Neal, Aaron; National Institute of Allergy and Infectious Diseases<br>Lau, Chuen-Yen; National Cancer Institute<br>Lane, C; National Institute of Allergy and Infectious Diseases

**Primary Subject Heading**:	Infectious diseases
Secondary Subject Heading:	Paediatrics
Keywords:	Epidemiology < TROPICAL MEDICINE, INFECTIOUS DISEASES, PAEDIATRICS

SCHOLARONE™
Manuscripts

I, the Submitting Author has the right to grant and does grant on behalf of all authors of the Work (as defined in the below author licence), an exclusive licence and/or a non-exclusive licence for contributions from authors who are: i) UK Crown employees; ii) where BMJ has agreed a CC-BY licence shall apply, and/or iii) in accordance with the terms applicable for US Federal Government officers or employees acting as part of their official duties; on a worldwide, perpetual, irrevocable, royalty-free basis to BMJ Publishing Group Ltd ("BMJ") its licensees and where the relevant Journal is co-owned by BMJ to the co-owners of the Journal, to publish the Work in this journal and any other BMJ products and to exploit all rights, as set out in our licence.

The Submitting Author accepts and understands that any supply made under these terms is made by BMJ to the Submitting Author unless you are acting as an employee on behalf of your employer or a postgraduate student of an affiliated institution which is paying any applicable article publishing charge ("APC") for Open Access articles. Where the Submitting Author wishes to make the Work available on an Open Access basis (and intends to pay the relevant APC), the terms of reuse of such Open Access shall be governed by a Creative Commons licence – details of these licences and which Creative Commons licence will apply to this Work are set out in our licence referred to above.

Other than as permitted in any relevant BMJ Author's Self Archiving Policies, I confirm this Work has not been accepted for publication elsewhere, is not being considered for publication elsewhere and does not duplicate material already published. I confirm all authors consent to publication of this Work and authorise the granting of this licence.

For peer review only

Epidemiology of Community-acquired pneumonia among hospitalized children in Indonesia: a multicenter, prospective study

AUTHORS:

Dewi Lokida[1], Helmia Farida[2], Rina Triasih[3], Yan Mardian[4*], Herman Kosasih[4], Adhella Menur Naysilla[4], Arif Budiman[1], Chakrawati[1], Moh. Syarofil Anam[2], Dwi Wastoro[2], Mujahidah[2], Setya Dipayana[2], Amalia Setyati[3], Abu Tholib Aman[3], Nurhayati Lukman[4], Muhammad Karyana[5], Ahnika Kline[6], Aaron Neal[6], Chuen-Yen Lau[7], H. Clifford Lane[6]

*Correspondence to Dr. Yan Mardian: ymardian@ina-respond.net

AFFILIATIONS:

1. Tangerang District Hospital, Tangerang, Indonesia
2. Dr. Kariadi Hospital/Diponegoro University, Semarang, Indonesia
3. Dr. Sardjito Hospital/Universitas Gadjah Mada, Yogyakarta, Indonesia,
4. Indonesia Research Partnership on Infectious Disease, Jakarta, Indonesia
5. National Institute of Health Research and Development, Ministry of Health, Republic of Indonesia, Jakarta, Indonesia
6. National Institute of Allergy and Infectious Diseases, National Institutes of Health, Bethesda, MD, United States
7. National Cancer Institute, National Institutes of Health, Bethesda, MD, United States

ABSTRACT:

Objective:

To identify etiologies of childhood community-acquired pneumonia (CAP) based on a comprehensive diagnostic approach.

Design:

"Partnerships for Enhanced Engagement in Research - Pneumonia in Pediatrics (PEER-PePPeS)" study was an observational prospective-cohort study conducted from July 2017 through September 2019.

Setting:

For peer review only

For peer review only - http://bmjopen.bmj.com/site/about/guidelines.xhtml

Government referral teaching hospitals and satellite sites in three cities in Indonesia: Semarang, Yogyakarta, and Tangerang.

Participants:

Hospitalized children aged 2–59-months who met the criteria for pneumonia were eligible. Children were excluded if they had been hospitalized for >24 hours; had malignancy or history of malignancy; a history of long-term (>2 months) steroid therapy, or conditions that might interfere with compliance with study procedures.

Main outcome(s) measure(s):

Causative bacterial, viral, or mixed pathogen(s) for pneumonia were determined using microbiological, molecular, and serologic tests from routinely collected specimens (blood, sputum, and nasopharyngeal swabs). We applied a previously published algorithm (PEER-PePPeS rules) to determine the causative pathogen(s).

Results:

188 subjects were enrolled. Based on our algorithm, 48 (25.5%) had a bacterial infection, 31 (16.5%) had a viral infection, 76 (40.4%) had mixed bacterial and viral infections, and 33 (17.6%) were unable to be classified. The five most common causative pathogens identified were *Haemophilus influenzae* non-type B (N=73, 38.8%), respiratory syncytial virus (RSV) (N=51, 27.1%), *Klebsiella pneumoniae* (N=43, 22.9%), *Streptococcus pneumoniae* (N=29, 15.4%), and Influenza virus (N=25, 13.3%). RSV and Influenza virus diagnoses were highly associated with Indonesia's rainy season (November-March). The polymerase chain reaction (PCR) assays on induced sputum (IS) specimens captured most of the pathogens identified in this study.

Conclusions:

Our study found that *H. influenzae* non-type B and RSV were the most frequently identified pathogens causing hospitalized CAP among Indonesian children aged 2-59 months old. Our study also highlights the importance of PCR for diagnosis and by extension, appropriate use of antimicrobials.

Keywords: Pneumonia; Children; Indonesia; Etiology; Epidemiology

STRENGTHS AND LIMITATIONS OF THIS STUDY

- Prospective multisite study conducted over 27-months
- Used a comprehensive diagnostic approach (culture, molecular testing, and paired serologic assays) to identify causative pathogens from routinely collected specimens (blood, sputum, and nasopharyngeal swabs)

For peer review only

- The relatively small sample size, geographic limitation to the island of Java and observational design limit generalizability and causal inference
- We did not collect lung aspirates or pleural fluid specimens, which are preferred for determination of pneumonia etiology, and did not include healthy control children, limiting ability to estimate the adjusted population attributable fraction (aPaF) for each pathogen.
- Several cases of pneumonia were attributed to unknown etiology, which could be due to administration of antibiotics before culture, poor sputum quality, limited bacterial and viral panels, lack of fungal testing or another factor

INTRODUCTION

Pneumonia is the leading infectious cause of child mortality, with a greater burden in low- and middle-income countries (LMICs).[1] In Indonesia, pneumonia contributed to 15% of childhood deaths and was the second leading cause of death amongst children under five years in 2017.[2] Indonesian practice guidelines are adapted from the World Health Organization (WHO) guidelines, which are based on 1970's – 1990's data showing bacteria such as *Haemophilus influenzae* type b (Hib) and *Streptococcus pneumoniae* caused the majority of fatal pneumonias in children.[3–5] Therefore empiric antibiotics are considered first-line treatment for children with community-acquired pneumonia (CAP).[6–8] Despite evidence that appropriate antibiotics are lifesaving, rational selection of antibiotics for pneumonia is hampered by low adherence to guidelines and scarcity of point-of-care diagnostics.[9–11] Consequently, healthcare providers, particularly those in LMIC, are likely to overtreat non-bacterial pneumonia with antibiotics.[11,12]

Several recent studies of community-acquired pneumonia (CAP) in children have highlighted the role of viral etiologies. Increased recognition of viral etiologies of CAP is likely due to both enhanced molecular diagnostic capacity and wide deployment of Hib and pneumococcal conjugate vaccines [PCV].[13,14] Treatment of non-bacterial pneumonia with antibiotics may engender avoidable antimicrobial resistance. Thus, current data on the etiologies of childhood pneumonia is needed and should be regularly evaluated to inform vaccination policies, empiric management decisions, and targeted treatment.[12]

From a diagnostic standpoint, direct demonstration of organisms by culture (or staining) of lung aspirates has been the standard for determining microbial etiology of CAP.[15] In the current era, many use less-invasive biological specimens (e.g. blood, naso/oropharyngeal secretions, bronchoalveolar lavage, or induced sputum) and employ diverse methods (e.g. culture, PCR, antigen detection, or paired serology) to identify organisms.[16] However, such comprehensive methods are

For peer review only - http://bmjopen.bmj.com/site/about/guidelines.xhtml

costly and often require specialized equipment and human resources, limiting feasibility in low-resource settings.[17,18]

Prospective community-based cohort studies that define pathogen(s) causing CAP in Indonesian children are scarce. We conducted a “Partnerships for Enhanced Engagement in Research - Pneumonia in Pediatrics (PEER-PePPeS)” study, which aimed to identify etiologies of childhood CAP using comprehensive diagnostic methods.

METHODS

Study design and study sites

PEER-PePPeS was a multi-site observational cohort study seeking to determine etiologies of CAP amongst children aged 2–59 months in Indonesia. The study was conducted by the Indonesia Research Partnership on Infectious Disease (INA-RESPOND) and enrolled participants initially at three government referral teaching hospitals in three provinces: Kariadi Hospital (Central Java), Sardjito Hospital (Yogyakarta), and Tangerang District Hospital (Banten), as shown in **Supplementary Fig. 1.** Satellite sites located near the primary sites were added during the study to facilitate subject recruitment.

Study Definitions

In this study, pneumonia in children was defined as cough or fever with at least one of the following: shortness of breath (indicated by at least one of the following signs: head bobbing; nasal flaring; chest indrawing or intercostal retracting), tachypnea, grunting, crackles, rhonchi, decreased vesicular breath sounds, bronchial breath sounds or chest x-ray findings consistent with pneumonia. Tachypnea was defined as respiratory rate >50/min for infants 2–12 months and >40/min for children >12–60 months.[19] Abnormal chest x-ray findings consistent with pneumonia were defined as presence of either focal or diffuse infiltrates, a silhouette sign, pleural effusion, or air bronchogram.[20] Chest x-rays were read by the pediatrician.

Based on WHO classification and treatment of childhood pneumonia at health facilities (2014 version), for children 2–59 months of age, severe pneumonia is defined as pneumonia (tachypnea and/or chest indrawing) accompanied by presence of any danger signs, including inability to drink, persistent vomiting, convulsions, lethargy or loss of consciousness, stridor in a calm child, or severe malnutrition.[19]

Study Participants

For peer review only

PEER-PePPeS study enrolled children aged 2-59 months, who were hospitalized between July 18th, 2017 until September 25th, 2019, and met the definition for pneumonia. Eligible subjects were enrolled within 24 hours of admission. Children were excluded if they had been hospitalized for >24 hours; had a malignancy or history of malignancy; a history of long term (≥2 months) steroid therapy; or conditions that might interfere with compliance with study procedures (e.g., very ill patients for whom specimens could not be obtained or living outside the area for which follow-up was practical).

Study Procedures

Demographic and anthropometric data, current signs and symptoms, pregnancy history, vaccination status, breastfeeding history, antibiotic and steroid exposure, family history, medical history, risk factors, hematology profiles, chemistry results, and chest x-ray (per standard of care) were collected at enrollment. Clinical examination (vital signs, general examination, lung auscultation, SpO2); nasopharyngeal (NP) swab for molecular tests; induced sputum (IS) for culture and molecular tests; collection of blood specimens for routine blood count, cultures, molecular tests, serologic tests, C-reactive protein (CRP), and procalcitonin (PCT) were also performed. We prospectively followed subjects daily until hospital discharge; data on vital signs, respiratory signs, intensive care admission, intubation, complications, and treatment were collected. On Day 14, we performed clinical examinations and collected convalescent sera for serology tests; subjects discharged before day 14 returned to clinic for their evaluation. We conducted a telephone interview on Day 30 (±4 days) to assess clinical outcome.

This study used several widely available bacterial and viral respiratory molecular pathogen panels and serologic assays.[21–24] NP and IS specimens were tested with a PCR panel that included twelve-viruses (influenza A, influenza B, adenovirus, enterovirus, bocavirus, respiratory syncytial virus (RSV) A, RSV B, human metapneumovirus (hMPV), rhinovirus, parainfluenza virus (PIV) 1-4, coronavirus OC43, and coronavirus NL63). NP specimens were evaluated by PCR for five bacteria (*Haemophilus influenzae*, *Streptococcus pneumoniae*, *Moraxella catarrhalis*, *Staphylococcus aureus*, and *Klebsiella pneumoniae*), while IS specimens were tested for nine (*Haemophilus influenzae, Streptococcus pneumoniae, Mycoplasma pneumoniae, Chlamydia pneumoniae, Bordetella pertussis, Moraxella catarrhalis, Staphylococcus aureus*, *Klebsiella pneumoniae,* and *Legionella pneumoniae*). Good quality (<10 squamous epithelial cells per low power field[12]) IS specimens underwent culture and gram stain.[25] For whole blood, qPCR was performed for three bacteria (*Haemophilus influenzae*, *Streptococcus pneumoniae*, and *Staphylococcus aureus)*. Serologic testing for seven viruses (influenza A, influenza B, adenovirus, parvovirus B19, echovirus/enterovirus, RSV, parainfluenza virus) and four

For peer review only - http://bmjopen.bmj.com/site/about/guidelines.xhtml

bacteria (*Mycoplasma pneumoniae, Chlamydia pneumoniae, Legionella pneumoniae,* and *Bordetella pertussis*) was performed using paired acute-convalescent sera.

Blood culture, IS culture and Gram stain, routine blood count, CRP, PCT, and chest x-ray were performed by the laboratory/radiology department at the hospital site. qPCR and serology assays were performed retrospectively at the INA-RESPOND Reference Laboratory located in Tangerang District Hospital. Details of blood culture, sputum culture, molecular and serology test techniques are shown in **Supplementary Table 1**.

Pathogen Identification

Causative bacterial, viral, or mixed pathogen for the pneumonia was determined based on an algorithm (PEER-PePPeS rules) for interpretation of microbiological, molecular, and serologic test results published previously.[12] In brief, we considered all organisms detected by blood culture, detected by whole blood PCR, or that grew from good quality IS specimen in high quantities with a compatible primary Gram stain as potential causative bacterial pathogens. Bacteria commonly considered contaminants were excluded. For the nasopharynx, potential colonizing bacteria (e.g. *H. influenzae*, *S. pneumoniae*, and *S. aureus*) and potential innocent bystander viruses (e.g. bocavirus, adenovirus, non-SARS human Coronavirus (hCoVs), enterovirus, and rhinovirus) were determined to be causative based on a PCR density cut-off and/or serodiagnosis criteria for paired acute and convalescent sera (seroconversion or a two to four-fold increase in antibody titers in the convalescent specimen).[12]

Data collection and statistical analysis

Data were recorded on paper case report forms and entered in duplicate into OpenClinica (OpenClinica, LLC, MA, USA) by research staff. Categorical variables were summarized using absolute values and percentages, and continuous variables as medians and interquartile ranges (IQRs). Differences in categorical variables were compared using Pearson $\chi2$ or Fisher's exact test when the expected values in any of the contingency table cells were below 5. Differences in continuous variables were compared using One-way ANOVA or Kruskal-Wallis H-test for data which did not follow the normal distribution based on Levene's test. Statistical analyses were performed using Statistical Package for Social Science (SPSS) software version 23 (IBM Corporation, Armonk, NY, USA). All p-values were two-sided. Level of significance was set at $P < 0.05$.

Patient and public involvement statement

For peer review only - http://bmjopen.bmj.com/site/about/guidelines.xhtml

Patients or the public were not involved in study design or study conduct at any stage from inception to completion and dissemination of this project. Patients who met the eligibility criteria as described above were recruited to this study.

RESULTS

Study Population

Of 444 children who were hospitalized with CAP, 188 (42.3%) were eligible and enrolled in the study. Of 256 screening failures, 31.8% were due to hospitalization >24 hours at the time of screening and 22.1% to circumstances that might interfere with the study procedures. Of the 188 enrolled children, 184 (97.9%) had radiologic evidence of pneumonia. 179 (95.1%) subjects completed the study, including 19 (10.1%) who died. Eight subjects (4.3%) were lost to follow up, and one subject (0.5%) withdrew from the study. The study flow is shown in **Figure 1**.

Demographic and clinical characteristics are presented in **Table 1**. Age, gender, laboratory values, and pneumonia severity by WHO classification were similar across the three study sites. The median age was nine months (IQR, 5 to 20), and 54.7% of subjects were male. The most common comorbid conditions / medical histories were developmental delay (27.7%), congenital heart disease (26.1%), low birth weight (24.4%), and severe malnutrition (18.6%), with subjects from Yogyakarta site having the greatest proportion of those comorbidities. The percentage of subjects who had been vaccinated (age-adjusted) against pneumococcus, influenza, Hib-DPT, and measles vaccines were 2.1%, 1.1%, 55.9%, and 75.0%, respectively.

Table 1. Baseline Characteristics of Subjects.

Demographic Characteristics	All (N=188)	Semarang (N=47)	Yogyakarta (N=52)	Tangerang (N=89)	P-value
Age, median (IQR) months	9 (5 – 20)	9 (5.5 – 21)	8 (4 – 13.3)	11 (5-20)	0.442
Gender, Male, (%)	103 (54.7)	29 (61.7)	26 (50)	48 (53.9)	0.493
Household Characteristics, (%):					
• Low Education of Parents*	163 (86.7)	37 (78.7)	43 (82.7)	84 (94.3)	0.019
• Living in a dense neighborhood†	121 (64.4)	19 (40.4)	42 (80.8)	60 (67.4)	<0.001
• Exposure to cigarette smoke	120 (63.8)	24 (51.1)	27 (51.9)	69 (77.5)	0.001
• Sick household contact <14 days	109 (58.0)	22 (46.8)	43 (82.7)	44 (49.4)	<0.001
• Living near waste disposal	70 (37.2)	12 (25.5)	29 (55.8)	29 (32.6)	0.004
• Attending daycare	4 (2.1)	2 (4.3)	1 (1.9)	1 (1.1)	0.374
Medical history (%):					

For peer review only - http://bmjopen.bmj.com/site/about/guidelines.xhtml

Demographic Characteristics	All (N=188)	Semarang (N=47)	Yogyakarta (N=52)	Tangerang (N=89)	P-value
• Developmental delay	52 (27.7)	16 (34.0)	21 (40.4)	15 (16.8)	0.003
• Congenital heart disease	49 (26.1)	16 (34.0)	24 (46.2)	9 (10.1)	<0.001
• Low birth weight	46 (24.4)	12 (25.5)	20 (38.5)	14 (15.7)	0.011
• Severe malnutrition‡	35 (18.6)	10 (21.3)	13 (25.0)	12 (13.5)	0.205
• Premature baby	34 (18.1)	4 (8.5)	16 (30.8)	14 (15.7)	0.012
• Neurological disorder	25 (13.3)	5 (10.6)	17 (32.7)	3 (3.4)	<0.001
• Tuberculosis (recent/cured)	10 (5.3)	4 (8.5)	2 (3.8)	4 (4.5)	0.588
• Asthma	9 (4.8)	3 (6.4)	1 (1.9)	5 (5.6)	0.563
• HIV disease§	2 (1.1)	1 (2.1)	1 (1.9)	0 (0)	0.315
Immunization history, fully vaccinated for age‖ (%):					
• Measles	141 (75.0)	38 (80.9)	41 (78.8)	62 (69.7)	0.175
• DPT-Hib	105 (55.9)	30 (63.8)	25 (48.1)	50 (56.2)	0.233
• Pneumococcus	4 (2.1)	0 (0)	4 (7.7)	0 (0)	0.009
• Influenza	2 (1.1)	0 (0)	2 (3.8)	0 (0)	0.132
Symptoms and signs (%):					
• Shortness of breath	174 (92.6)	41 (87.2)	48 (92.3)	85 (95.5)	0.214
• Cough	171 (91.0)	40 (85.1)	42 (80.8)	89 (100)	<0.001
• Intercostal retraction	171 (91.0)	43 (91.5)	52 (100)	76 (85.4)	0.005
• Rhonchi	168 (89.4)	42 (89.4)	39 (75.0)	87 (97.8)	<0.001
• Fever	152 (80.9)	34 (72.3)	35 (67.3)	83 (93.3)	<0.001
• Chest indrawing	125 (66.5)	36 (76.6)	43 (82.7)	46 (51.7)	<0.001
• Fast breathing	80 (42.6)	15 (31.9)	43 (82.7)	22 (24.7)	<0.001
• SpO_2 <90% and/or Cyanosis	43 (22.9)	7 (14.9)	17 (32.7)	19 (21.3)	0.098
• Diarrhea	36 (19.1)	6 (12.8)	4 (7.7)	26 (29.2)	0.003
• Wheezing	35 (18.6)	9 (19.1)	10 (19.2)	16 (18.0)	1.000
• Vomiting	14 (7.4)	4 (8.5)	5 (9.6)	5 (5.6)	0.595
• Inability to drink	13 (6.9)	4 (8.5)	5 (9.6)	4 (4.5)	0.425
• Decreased Consciousness	7 (3.7)	1 (2.1)	1 (1.9)	5 (5.6)	0.612
• Seizure	6 (3.2)	1 (2.1)	0 (0)	5 (5.6)	0.203
Leukocyte count, median (IQR) × 10^3/uL	14.0 (10.4 – 18.9)	14.9 (11.1 – 18.8)	12.1 (9.8 – 17.8)	14.0 (10.4 – 19.0)	0.356
Neutrophil-lymphocyte ratio (NLR), median (IQR)	1.4 (0.9 – 2.8)	1.3 (0.9 – 2.6)	1.0 (0.6 – 2.0)	1.9 (1.1 – 3.2)	0.367
CRP, median (IQR) mg/L	9.0 (3.6 – 28.0)	11.8 (1.6 – 23.3)	9.0 (4.9 – 21.8)	8.4 (1.5 – 34.1)	0.665
PCT, median (IQR) ng/mL	0.2 (0.1 – 1.7)	0.2 (0.1 – 1.5)	0.2 (0.1 – 1.0)	0.2 (0.1 – 2.6)	0.912

For peer review only - http://bmjopen.bmj.com/site/about/guidelines.xhtml

For peer review only

Demographic Characteristics	All (N=188)	Semarang (N=47)	Yogyakarta (N=52)	Tangerang (N=89)	P-value
Severe pneumonia (WHO Classification 2014 version) (%)	89 (47.3)	26 (55.3)	26 (50.0)	37 (41.6)	0.281
CXR Findings (%):					
• Interstitial infiltrate	131 (69.7)	26 (55.3)	30 (57.7)	75 (84.3)	<0.001
• Alveolar infiltrate	125 (66.5)	41 (87.2)	44 (84.6)	40 (44.9)	<0.001
• Pleural effusion	5 (2.7)	1 (2.1)	2 (3.8)	2 (2.2)	0.850
Antibiotic administration prior to blood culture (%):	150 (79.8)	39 (83.0)	49 (94.2)	62 (69.7)	0.002

[*]Low education of parents was defined by highest level of parents' formal education being high school diploma or less; [†]A densely populated neighborhood was defined as >200 people/km^2 or <8 m^2/person in the subject's home; [‡] Severe malnutrition was defined as weight for height below -3 standard deviations from the median of the WHO Child Growth Standards; [§]Subjects were tested for HIV infection if a parent / guardian provided consent and a specimen was available (n=160); [||]Full vaccination was defined as being up to date for age per vaccination schedule at study enrollment.

The most common symptoms were shortness of breath (92.6%), cough (91.0%), and fever (80.9%). Signs noted during the initial examination included intercostal retraction (91.0%), rhonchi (89.4%), and chest indrawing (66.5%). Of 188 subjects, 172 (91.4%) and 167 (88.8%) had CRP and PCT measured with median values of 9.0 (IQR, 3.6 – 28.0; Ref range ≤5) mg/L and 0.2 (IQR, 0.1 – 1.7; Ref range ≤0.15) ng/mL, respectively. Interstitial infiltrate (69.7%) was the most common radiographic finding. 47.3% of cases were classified as severe pneumonia according to the WHO classification system. All 188 enrolled cases were treated with antibiotics, and 150 of them (79.8%) had received 1 to 2 doses of antibiotics prior to collection of blood culture in the emergency unit, with the combination of ampicillin and gentamicin (34.6%), cefotaxime (17.0%), and ceftriaxone (14.4%) being the three most frequent regimens used. Details of antibiotic regimens administered before blood culture, including dosage and given frequency, are presented in **Supplementary Table 2.**

Detection of Pathogens

Blood and sputum cultures were performed on specimens from 184 (97.9%) and 183 (97.3%) subjects, respectively. A total of 150 (79.8%) children received antibiotics prior to collection of blood for culture. Seventy-five (41.0%) sputum culture isolates were analyzed from specimens meeting the required quality criteria. A NP or OP swab was obtained from 187 (99.5%) subjects, IS for PCR from 176 (93.6%), whole blood for PCR from 163 (86.7%), and paired acute-convalescent serum specimens for serology from 116 (61.7%) (**Fig. 1**).

The PEER-PePPeS algorithm was used to determine the causative pathogen(s) from those identified by culture, molecular, and serologic assay. Amongst the 188 study participants, 48 (25.5%)

For peer review only

had bacterial infection, 31 (16.5%) had viral infection, 76 (40.4%) were of mixed bacterial and viral etiology, and 33 (17.6%) were of unknown etiology (**Fig. 2, Panel A**). Mixed infection, the most common overall etiology, was seen in 38.7% of 2-11 month-olds and in 42.7% of 12-59 month-olds (**Fig 2. Panel B**). Mixed infection was also the predominant etiology across all study sites (**Supplementary Fig. 2**). *H. influenzae* non-type B (N=73, 38.8%), RSV (N=51, 27.1%), *K. pneumoniae* (N=43, 22.9%), *S. pneumoniae* (N=29, 15.4%), Influenza virus (N=25, 13.3%), *S. aureus* (N=20, 10.6%), PIV (N=17, 9.0%), hMPV (N=11, 5.8%), Rhinovirus (N=10, 5.3%), and *B. pertussis* (N=7, 3.7%) were the top ten pathogens identified, more commonly appearing in mixed infection as opposed to as a sole pathogen (**Fig 2. Panel C**). Influenza virus was significantly higher in the age group 12-59 mo vs 2-11 mo (N=16, 64%, P=0.027), while *S. aureus* was significantly more common in 2-11 mo vs 12-59 mo (N=16, 80%, P=0.024). Though not statistically significant, other pathogens trended toward more frequent detection in age group 2-11 mo (except *B. pertussis*) (**Fig 2. Panel D**). Amongst 76 mixed infection cases, RSV + *H. influenzae* non-type B was the most common co-infection (N=22, 28.9%), followed by RSV + *S. pneumoniae* (N=10, 13.2%), Influenza virus + *H. influenzae* non-type B (N=10, 13.2%), RSV + *K. pneumoniae* (N=9, 11.8%), and Parainfluenza virus + *H. influenzae* non-type B (N=9, 11.8%) (Data not shown).

We observed no difference in pathogen distribution by pneumonia severity based on WHO classification system (**Supplementary Table 3 and Supplementary Fig. 3**). By pathogen, there was no significant difference in distribution between pneumonia severity status or mortality, except for *S. pneumoniae* which was found in significantly more severe cases using the WHO system (P=0.033) (**Supplementary Table 3**).

A comparison of positivity rates for each causative pathogen by detection method is shown in **Table 2**. Overall, PCR captured more bacterial pathogens than culture and more viral pathogens than acute-convalescent paired serology. Paired serology was generally helpful in identifying atypical bacteria, such as *C. pneumoniae* and *L. pneumophila*, and upper respiratory tract viruses, such as Rhinovirus and Enterovirus. When comparing blood and IS culture, IS yielded more positive bacterial pathogen results. Similarly, IS PCR captured more pathogens than NP/OP PCR.

Table 2. Causative Pathogens per PEER-PePPeS Rules by Detection Method

For peer review only - http://bmjopen.bmj.com/site/about/guidelines.xhtml

Pathogen	N	Blood culture N (%)	IS culture N (%)	Whole blood PCR N (%)	NP / OP PCR N (%)	IS PCR N (%)	Serology Test N (%)
**Gram-positive cocci bacteria**							
S. pneumoniae	29	1 (3.4%)	3 (10.3%)	--	21 (72.4%)	28 (96.6%)	
S. aureus	20	--	7 (35%)	--	11 (55%)	19 (95%)	
S. mitis	4	--	4 (100%)				
S. pyogenes	1	--	1 (100%)				
**Gram-negative cocci bacteria**							
M. catarrhalis	2	--	2 (100%)		2 (100%)	2 (100%)	
**Gram-negative rods bacteria**							
H. inf non-type b	73	--	--	8 (10.9%)	60 (82.2%)	71 (98.6%)	
K. pneumoniae	43	--	17 (39.5%)		2 (4.7%)	34 (79.1%)	
B. pertussis	7	--	--			7 (100%)	
E. coli	5	1 (20%)	4 (80%)				
P. aeruginosa	4	--	4 (100%)				
A. baumannii	3	--	3 (100%)				
H. inf type b	2	--	--	--	--	2 (100%)	
N. meningitidis	1	1 (100%)	1 (100%)				
**Atypical-bacteria**							
C. pneumoniae	5	--	--			--	5 (100%)
M. pneumoniae	5	--	--			5 (100%)	1 (20%)
L. pneumophila	1	--	--			--	1 (100%)
**Virus**							
RSV	51				36 (70.6%)	45 (88.2%)	10 (19.6%)
RSV A	15				10 (66.7%)	13 (86.7%)	
RSV B	36				26 (72.2%)	32 (88.8%)	
Influenza virus	25				16 (64%)	22 (88%)	9 (36%)
inf A (H1N1)	7				7 (100%)	7 (100%)	7 (70%)
inf A (H3N2)	3				3 (100%)	3 (100%)	
inf B	14				6 (42.9%)	12 (85.7%)	2 (14.3%)
PIV	17				16 (94.1%)	15 (88.2%)	3 (17.6%)
PIV 1	5				5 (100%)	4 (80%)	3 (17.6%)
PIV 2	0				--	--	
PIV 3	11				10 (90.9%)	10 (90.9%)	
PIV 4	1				1 (100%)	1 (100%)	
hMPV	11				5 (45.5%)	10 (90.9%)	
Rhinovirus	10				10 (100%)	6 (60%)	4 (40%)
Enterovirus	5				3 (60%)	3 (60%)	3 (60%)
Bocavirus	3				2 (66.7%)	3 (100%)	
hCoV-NL63	2				2 (100%)	2 (100%)	

Grey-box indicates the assay was not performed

Mortality

Nineteen (10.1%) of the 188 subjects died during the 30-day study period. Seven (36.8%) of these 19 were male, and most (N=17, 89.5%) were less than 1 year old. Among the 19 deceased subjects, median study duration was 12 (IQR, 4 – 17.5) days; eight (42.1%) were admitted to ICU, and

For peer review only

For peer review only - http://bmjopen.bmj.com/site/about/guidelines.xhtml

six (31.6%) received mechanical ventilation. Twelve (63.2%) died due to respiratory failure, three (15.8%) due to sepsis, and three (15.8%) for unknown reasons after discharge (data not shown). Most deaths occurred in the 2-11 mo age group compared with the 12-59 mo age group (78.9% vs. 21.1%, *p*=0.036). Causative pathogens for deceased subjects were bacterial-only in seven (36.8%), viral-only in two (10.5%), mixed in five (26.3%), and unknown in five subjects (26.3%). There were no significant differences in pathogen distribution between subjects that survived and died. *H. influenzae* non-type B was the most common pathogen identified in deceased subjects (N=8, with the case fatality rate [CFR] in this study of 11.0%), followed by *K. pneumoniae* (N=6, CFR of 13.9%), Influenza virus (N=3, CFR of 12.0%), *B. pertussis* (N=2, CFR of 28.6%), and RSV (N=2, CFR of 3.9%) (**Supplementary Table 3**). Pre-existing conditions amongst deceased subjects included congenital heart disease (N=10, 52.6%), severe malnutrition (N=7, 36.8%), and developmental delay (N=7, 36.8%). A clinical summary of the fatal cases is shown in **Supplementary Table 4**.

Seasonality

During the 27-month study period, infections caused by RSV and influenza were seen year-round with peak activity occurring during the wet season (November to March) in Indonesia (66.7%, $p<0.001$; and 64.0%, $p=0.012$, respectively). However, there was little variation in detection of the most common respiratory bacterial infections by month and season. *H. influenzae* non-type B shows peaks in August (N=12, 16.4%) and March (N=11, 15.1%), while *K. pneumoniae* and *S. pneumoniae* fluctuate at lower levels throughout the year (**Figure 3**).

DISCUSSION:

PEER-PePPeS, a prospective multisite study, characterized the current epidemiology of CAP in children 2-59 months old in Indonesia. No recent prospective Indonesian studies address this topic. Our study found: (1) mixed bacterial and viral infection is the most frequent (N=76, 40.4%) cause of childhood CAP, irrespective of age group and pneumonia severity; (2) bacterial infections were common (66% of cases) with *H. influenzae* non-b type, *K. pneumoniae*, and *S. pneumoniae* as the three most common bacterial etiologies; (3) viral pathogens were also common (57% of PEER-PePPeS subjects), with 16.5% of cases attributed to virus only and RSV and Influenza Virus being the most common viruses identified; and (4) PCR on IS specimens was the most sensitive assay for pathogen identification.

While our findings are consistent with other studies, clinical significance of mixed infection remains controversial. It is unclear if both agents act as true pathogens. [22,26] PEER-PePPeS did not

For peer review only - http://bmjopen.bmj.com/site/about/guidelines.xhtml

For peer review only

demonstrate a correlation of mixed infection with pneumonia severity and 30-day mortality. Many deaths occurred at a younger age (<1 year old) and with comorbidities, such as congenital heart disease and severe malnutrition, similar to previous reports. [27,28] Such factors should be considered in prevention and management of childhood pneumonia to reduce mortality rate.

In recent years, there has been an increased focus on the role of respiratory viruses in childhood pneumonia, partly attributable to use of conjugate pneumococcal and Hib vaccines and increased detection by PCR.[21,22,29,30] In PEER-PePPeS, viruses were found in 57% of subjects (virus only + mixed infection), with 16.5% of cases attributed to virus only. Thus, many patients probably received unnecessary antibiotics when covered empirically per current Indonesian guidelines. Improving ability to discriminate between viral and bacterial infections would facilitate optimization of antibiotic administration and counter antimicrobial resistance.[31]

RSV and influenza virus were the most commonly detected viruses in this study and may be associated with Indonesia's wet/rainy season.[32–34] A high prevalence of RSV was also observed in the GABRIEL and PERCH international case-control studies of childhood pneumonia etiology.[22,30] In terms of mixed infections, we found that RSV + *H. influenzae* non-type B and RSV + *S. pneumoniae* were most common. Since respiratory viruses such as RSV can predispose to secondary bacterial infections, particularly *S. pneumoniae* and *H. influenzae*[35], and conversely bacteria can increase RSV susceptibility[35,36], these co-infections highlight the need for optimizing RSV surveillance, prevention and treatment.

Though influenza virus also increases risk for secondary bacterial infections and is a major cause of childhood morbidity and mortality worldwide, data from developing countries is scarce.[37] In a previous Indonesian study of hospitalized patients with a severe acute respiratory infection (SARI), the prevalence of the influenza virus was 10.6% in children under 5 years old, and was never diagnosed during hospitalization.[38] PEER-PePPeS confirms the need for improved diagnostic strategies, management optimization, and influenza vaccination in children. Of note, our study was conducted before identification of COVID-19 in Indonesia[39], so did not address the role of COVID-19 in childhood pneumonia.

We also found that 66% of cases were caused by bacterial infection (bacteria only + mixed infection). Overall, *H. influenzae* non-type B was the most common bacteria implicated, followed by *K. pneumoniae* and *S. pneumoniae*. *H. influenzae* non-type B predominance was also observed in a Malaysian study, where 90% of enrolled children were vaccinated against Hib as part of the national immunization program.[24] With Indonesia's moderate (56.4%) Hib vaccine coverage, high incidence of *H. influenzae* non-type B may represent its true prevalence or strains not covered by Hib

For peer review only - http://bmjopen.bmj.com/site/about/guidelines.xhtml

vaccine.[40] This finding agrees with current data that non-typeable *H. influenzae* (NTHi) can cause significant illness, and argues for strengthening pediatric diagnostic laboratory capacity.

Our identification of *K. pneumoniae* as the second most common bacterial etiology is consistent with high carriage rates (~7%) in healthy Indonesian children. Carriage has been related to poor food and water sanitation and may give rise to pneumonia, especially in children with malnutrition.[41] Given *K. pneumoniae's* potential for antibiotic resistance and high virulence of some strains, proactive detection and management strategies should be prioritized.[42]

The relatively low prevalence (15.4%) of *S. pneumoniae* in PEER-PePPes was surprising since carriage rates are high and PCV coverage low in Indonesia.[43] Low prevalence has also been reported from Malaysia, where PCV coverage is 8.7%[24] and in the PERCH study, reflecting temporal shifts in childhood pneumonia etiologies.[22] As only 4.8% of PEER-PePPeS subjects had received PCV, vaccination alone cannot account for the low *S. pneumoniae* prevalence. Antibiotic exposure prior to specimen collection may have reduced colonization density and lowered the yield of *S. pneumoniae* by both culture and PCR.[44] Moreover, our panel did not include *S. pneumoniae* paired serology, which may be useful to increase pneumococcal diagnosis in young children.[45] Nonetheless, *S. pneumoniae* remains an important etiological agent of severe/complicated CAP globally.[46] Our finding that *S. pneumoniae* was significantly associated with severe cases by the WHO classification system supports the need for ongoing surveillance, vaccination and prevention of transmission between adults and children.

Inclusion of several pathogen identification strategies in PEER-PePPes demonstrates the differential utility of assays and specimen types. Our findings highlight the value of molecular assays, especially in culture-negative cases where microorganisms may be nonrecoverable in culture due to prior antibiotics or presence of otherwise difficult to culture bacteria.[47,48] PCR is also less laborious and can identify genes associated with antibiotic resistance, though conventional culture methods are required to confirm phenotypic resistance.[49,50] Even with the limited PCR panels used in our study, molecular assays had greater sensitivity for identification of bacterial pathogens than blood or sputum culture.

Although sensitive for detection, PCR does not provide information regarding infectiousness or viability. Genome fragments from dead organisms may be detected, often at a low level, even after clinical resolution.[48] Furthermore, negative results may occur due to differential viral kinetics along the respiratory tract. Lower respiratory tract specimens, such as IS, should be sought as they originate from the site of infection.[12,13] Accordingly, we observed a higher yield from PCR on IS than NP specimens. We also found that the use of paired serologies increased the diagnostic yield and was

For peer review only - http://bmjopen.bmj.com/site/about/guidelines.xhtml

useful for pathogen confirmation, particularly in the setting of innocent bystander viruses and atypical bacteria.[12]

PEER-PePPeS used a comprehensive approach for pathogen detection to increase diagnostic yield. It also enrolled patients over a 27-month study period, facilitating assessment of seasonality. However, our study has several limitations. First, the relatively small sample size, and observational design may limit generalizability and causal inference. Second, most subjects (79.8%) received antibiotics before specimen collection in accordance with national guidelines. To address this, we enrolled subjects within 24 hours of admission, and specimens were collected as soon as possible to minimize the effects of antibiotics on culture results. Third, we did not enroll healthy control children, limiting the ability to estimate the adjusted population attributable fraction (aPaF) of each pathogen.[29,30] A healthy control group could have revealed baseline carriage rates, minimizing over-attribution of disease to non-pathogenic organisms.[21,22,29,30] Fourth, we did not collect lung aspirates or pleural fluid specimens, which are superior for determination of pneumonia etiology. [15] Fifth, several subjects had pneumonia of unknown etiology; this may have been due to administration of antibiotics before culture which could reduce sensitivity, poor IS quality, the limited panel of bacterial and viral pathogens tested, lack of fungal testing, or currently unrecognized causes of pediatric pneumonia.

In conclusion, the epidemiology of childhood CAP is constantly evolving in step with social and environmental factors and thus, should be regularly assessed. Our study found that *H. influenzae* non-type B and RSV were the most common pathogens causing hospitalized CAP among Indonesian children aged 2-59 months old, reflecting temporally dynamic etiologies of childhood CAP; studies from the 1970-1990s mainly detected *S. pneumoniae* and *H. influenzae* type B as the most important causes of childhood pneumonia in LMICs.[3–5] PCR on IS demonstrated the best sensitivity for pathogen identification. We recommend incorporating molecular assays for pathogen detection, preferably multiplexed point-of-care assays, into practice guidelines. Improvements in Indonesia's lab infrastructure during the COVID-19 pandemic can be leveraged to facilitate use of molecular assays for evaluation of childhood CAP. Optimization of pathogen detection to understand changing childhood CAP epidemiology will also inform public policy on prevention and management.

ACKNOWLEDGMENTS:

We are grateful to the parents and children who participated in this study. We thank the site study teams (Isabella Puspa Dewi, Fenny Aztari, Mohammad Rosyid Ridho, Aisyah Pratiwi, Niken Maretasari, Firsty Ilminova, Yanantri Binga Ramsif, Yuli Mawarti, Nenes Prastiwi, and Yanantri Binga Ramsif), INA-

For peer review only - http://bmjopen.bmj.com/site/about/guidelines.xhtml

RESPOND Reference Laboratory team (Gustiani, Deni Pepy, Wahyu Nawang Wulan, Rizki Amalia Sari, Yuanita Djajady, and Ungke Anton Jaya), US-NIAID and Indonesia NIHRD. We appreciate the collaboration from the hospital Directors at Tangerang District Hospital, An-Nisa Hospital, Kariadi Hospital, Adhyatma Hospital, Bhakti Wira Tamtama Hospital, and Sardjito Hospital. We also would like to thank Aly Diana for her assistance during manuscript submission.

CONTRIBUTORS:

DL, HF, RT, YM, HK, AMN, ATA, CYL, HCL designed and conceptualized the study. DL, HF, RT, AB, C, MSA, DW, M, SD, AS performed clinical assessments and were responsible for data entry. DL, HF, RT, YM, HK, AMN, NL, AK, CYL designed the methodology for pathogen identification. YM, HK, AMN performed data analysis, interpretation and drafted the first manuscript. DL, HK, ATA, MK, AN, CYL, HCL assisted with manuscript writing, analysis, and interpretation of data. All authors contributed to manuscript development, edited for critical content, and have approved the final version.

FUNDING STATEMENT:

This manuscript has been funded in whole or in part with MoH Indonesia, National Academy of Sciences (Sub-Grant Number: 2000007599), and Federal funds from the NIAID, NIH, under contract Nos. HHSN261200800001E and HHSN261201500003I. The content of this publication does not necessarily reflect the views or policies of the Department of Health and Human Services, nor does mention of trade names, commercial products, or organizations imply endorsement by the U.S. Government.

COMPETING INTERESTS:

The authors declare that the research was conducted in the absence of any commercial or financial relationships that could be construed as a potential conflict of interest.

ETHICS APPROVAL:

This study was approved by the Ethical Clearance Committee of Faculty of Medicine, Universitas Indonesia (No. 567/UN2.F1/ETIK/2017). The study was conducted in accordance with the Declaration of Helsinki. Written informed consent was obtained from parents or guardians before enrollment.

For peer review only

For peer review only - http://bmjopen.bmj.com/site/about/guidelines.xhtml

DATA AVAILABILITY STATEMENT:

Data are available upon reasonable request. The anonymized data set will be shared following the signing of a data-sharing agreement, with permission of the ethical clearance committee, study authors, and all project partners, exclusively for non-commercial purposes. Please contact the corresponding author with any queries.

REFERENCES:

1 McAllister DA, Liu L, Shi T, *et al.* Global, regional, and national estimates of pneumonia morbidity and mortality in children younger than 5 years between 2000 and 2015: a systematic analysis. *Lancet Glob Heal* 2019;**7**:e47–57. doi:10.1016/S2214-109X(18)30408-X

2 Windi R, Efendi F, Qona'ah A, *et al.* Determinants of acute respiratory infection among children under-five years in Indonesia. *J Pediatr Nurs* 2021;**S0882-5963**:00080–4. doi:10.1016/j.pedn.2021.03.010

3 Reñosa MD, Dalglish S, Bärnighausen K, *et al.* Key challenges of health care workers in implementing the integrated management of childhood illnesses (IMCI) program: a scoping review. *Glob Health Action* 2020;**13**:1732669. doi:10.1080/16549716.2020.1732669

4 Shann F. Etiology of severe pneumonia in children in developing countries. *Pediatr Infect Dis* 1986;**5**:247–52. doi:10.1097/00006454-198603000-00017

5 Feikin DR, Hammitt LL, Murdoch DR, *et al.* The Enduring Challenge of Determining Pneumonia Etiology in Children: Considerations for Future Research Priorities. *Clin Infect Dis* 2017;**64**:S188–96. doi:10.1093/cid/cix143

6 Bradley JS, Byington CL, Shah SS, *et al.* The Management of Community-Acquired Pneumonia in Infants and Children Older Than 3 Months of Age: Clinical Practice Guidelines by the Pediatric Infectious Diseases Society and the Infectious Diseases Society of America. *Clin Infect Dis* 2011;**53**:e25–76. doi:10.1093/cid/cir531

7 Harris M, Clark J, Coote N, *et al.* British Thoracic Society guidelines for the management of community acquired pneumonia in children: update 2011. *Thorax* 2011;**66**:ii1 LP-ii23. doi:10.1136/thoraxjnl-2011-200598

8 Mathur S, Fuchs A, Bielicki J, *et al.* Antibiotic use for community-acquired pneumonia in neonates and children: WHO evidence review. *Paediatr Int Child Health* 2018;**38**:S66–75. doi:10.1080/20469047.2017.1409455

9 Yusuf Y, Murni I, Setyati A. Irrational use of antibiotics and clinical outcomes in children with

For peer review only - http://bmjopen.bmj.com/site/about/guidelines.xhtml

pneumonia. *Paediatr Indones* 2018;**57**:211–5. doi:10.14238/pi57.4.2017.211-5

10 Tannous R, Haddad RN, Torbey P-H. Management of Community-Acquired Pneumonia in pediatrics: adherence to clinical guidelines. *Front Pediatr* 2020;**8**:1–6. doi:10.3389/fped.2020.00302

11 Ginsburg AS, Klugman KP. Vaccination to reduce antimicrobial resistance. *Lancet Glob Heal* 2017;**5**:e1176–7. doi:10.1016/S2214-109X(17)30364-9

12 Mardian Y, Menur Naysilla A, Lokida D, *et al.* Approach to Identifying Causative Pathogens of Community-Acquired Pneumonia in Children Using Culture, Molecular, and Serology Tests. Front. Pediatr. 2021;**9**:498. doi:10.3389/fped.2021.629318

13 Mardian Y, Kosasih H, Karyana M, *et al.* Review of Current COVID-19 Diagnostics and Opportunities for Further Development. Front. Med. 2021;**8**:562. doi:10.3389/fmed.2021.615099

14 Levine OS, O'Brien KL, Deloria-Knoll M, *et al.* The Pneumonia Etiology Research for Child Health Project: A 21st Century Childhood Pneumonia Etiology Study. *Clin Infect Dis* 2012;**54**:S93–101. doi:10.1093/cid/cir1052

15 Hammitt LL, Murdoch DR, Scott JAG, *et al.* Specimen collection for the diagnosis of pediatric pneumonia. *Clin Infect Dis* 2012;**54 Suppl 2**:S132–9. doi:10.1093/cid/cir1068

16 Zar HJ, Andronikou S, Nicol MP. Advances in the diagnosis of pneumonia in children. *BMJ* 2017;**358**:j2739. doi:10.1136/bmj.j2739

17 Thomas J, Pociute A, Kevalas R, *et al.* Blood biomarkers differentiating viral versus bacterial pneumonia aetiology: a literature review. *Ital J Pediatr* 2020;**46**:4. doi:10.1186/s13052-020-0770-3

18 Jiao F, Chen J, Wang M, *et al.* Determination of Procalcitonin, C-Reactive Protein and White Blood Cell Levels to Diagnose Community-Acquired Pneumonia (CAP). *Indian J Pediatr* 2019;**86**:763. doi:10.1007/s12098-019-02938-z

19 World Health Organization. *Revised WHO classification and treatment of childhood pneumonia at health facilities: evidence summaries*. Geneva PP - Geneva: : World Health Organization 2014. https://www.ncbi.nlm.nih.gov/books/NBK264162/

20 Walker CM, Abbott GF, Greene RE, *et al.* Imaging pulmonary infection: classic signs and patterns. *AJR Am J Roentgenol* 2014;**202**:479–92. doi:10.2214/AJR.13.11463

21 Jain S, Williams DJ, Arnold SR, *et al.* Community-Acquired Pneumonia Requiring Hospitalization among U.S. Children. *N Engl J Med* 2015;**372**:835–45. doi:10.1056/NEJMoa1405870

22 Group PER for CH (PERCH) S. Causes of severe pneumonia requiring hospital admission in

For peer review only

For peer review only - http://bmjopen.bmj.com/site/about/guidelines.xhtml

children without HIV infection from Africa and Asia: the PERCH multi-country case-control study. *Lancet (London, England)* 2019;**394**:757–79. doi:10.1016/S0140-6736(19)30721-4

23 Jansen RR, Schinkel J, Koekkoek S, *et al.* Development and evaluation of a four-tube real time multiplex PCR assay covering fourteen respiratory viruses, and comparison to its corresponding single target counterparts. *J Clin Virol Off Publ Pan Am Soc Clin Virol* 2011;**51**:179–85. doi:10.1016/j.jcv.2011.04.010

24 Nathan AM, Teh CSJ, Jabar KA, *et al.* Bacterial pneumonia and its associated factors in children from a developing country: A prospective cohort study. *PLoS One* 2020;**15**:e0228056. doi:10.1371/journal.pone.0228056

25 Lahti E, Peltola V, Waris M, *et al.* Induced sputum in the diagnosis of childhood community-acquired pneumonia. *Thorax* 2009;**64**:252 LP – 257. doi:10.1136/thx.2008.099051

26 Honkinen M, Lahti E, Österback R, *et al.* Viruses and bacteria in sputum samples of children with community-acquired pneumonia. *Clin Microbiol Infect* 2012;**18**:300–7. doi:https://doi.org/10.1111/j.1469-0691.2011.03603.x

27 Dean P, Florin TA. Factors Associated With Pneumonia Severity in Children: A Systematic Review. *J Pediatric Infect Dis Soc* 2018;**7**:323–34. doi:10.1093/jpids/piy046

28 Chen L, Miao C, Chen Y, *et al.* Age-specific risk factors of severe pneumonia among pediatric patients hospitalized with community-acquired pneumonia. *Ital J Pediatr* 2021;**47**:100. doi:10.1186/s13052-021-01042-3

29 Bhuiyan MU, Snelling TL, West R, *et al.* The contribution of viruses and bacteria to community-acquired pneumonia in vaccinated children: a case-control study. *Thorax* 2019;**74**:261 LP – 269. doi:10.1136/thoraxjnl-2018-212096

30 Bénet T, Sánchez Picot V, Messaoudi M, *et al.* Microorganisms Associated With Pneumonia in Children <5 Years of Age in Developing and Emerging Countries: The GABRIEL Pneumonia Multicenter, Prospective, Case-Control Study. *Clin Infect Dis an Off Publ Infect Dis Soc Am* 2017;**65**:604–12. doi:10.1093/cid/cix378

31 Lucien MAB, Canarie MF, Kilgore PE, *et al.* Antibiotics and antimicrobial resistance in the COVID-19 era: Perspective from resource-limited settings. *Int J Infect Dis* 2021;**104**:250–4. doi:https://doi.org/10.1016/j.ijid.2020.12.087

32 Kosasih H, Klimov A, Xiyan X, *et al.* Surveillance of Influenza in Indonesia, 2003–2007. *Influenza Other Respi Viruses* 2013;**7**:312–20. doi:https://doi.org/10.1111/j.1750-2659.2012.00403.x

33 Suryadevara M, Domachowske JB. Epidemiology and Seasonality of Childhood Respiratory Syncytial Virus Infections in the Tropics. Viruses. 2021;**13**. doi:10.3390/v13040696

For peer review only

For peer review only - http://bmjopen.bmj.com/site/about/guidelines.xhtml

34 Hirve S, Newman LP, Paget J, *et al.* Influenza Seasonality in the Tropics and Subtropics – When to Vaccinate? *PLoS One* 2016;**11**:e0153003. doi:10.1371/journal.pone.0153003

35 Griffiths C, Drews SJ, Marchant DJ. Respiratory syncytial virus: Infection, detection, and new options for prevention and treatment. *Clin Microbiol Rev* 2017;**30**:277–319. doi:10.1128/CMR.00010-16

36 Gulraiz F, Bellinghausen C, Bruggeman CA, *et al.* Haemophilus influenzae increases the susceptibility and inflammatory response of airway epithelial cells to viral infections. *FASEB J Off Publ Fed Am Soc Exp Biol* 2015;**29**:849–58. doi:10.1096/fj.14-254359

37 Dananché C, Sánchez Picot V, Bénet T, *et al.* Burden of Influenza in Less Than 5-Year-Old Children Admitted to Hospital with Pneumonia in Developing and Emerging Countries: A Descriptive, Multicenter Study. *Am J Trop Med Hyg* 2018;**98**:1805–10. doi:10.4269/ajtmh.17-0494

38 Aman AT, Wibawa T, Kosasih H, *et al.* Etiologies of severe acute respiratory infection (SARI) and misdiagnosis of influenza in Indonesia, 2013-2016. *Influenza Other Respi Viruses* 2021;**15**:34–44. doi:https://doi.org/10.1111/irv.12781

39 Setiawaty V, Kosasih H, Mardian Y, *et al.* The Identification of First COVID-19 Cluster in Indonesia. *Am J Trop Med Hyg*;**103**:2339–42. doi:10.4269/ajtmh.20-0554

40 Agrawal A, Murphy TF. Haemophilus influenzae infections in the H. influenzae type b conjugate vaccine era. *J Clin Microbiol* 2011;**49**:3728–32. doi:10.1128/JCM.05476-11

41 Helmia F, A. SJ, Hussein GM, *et al.* Nasopharyngeal Carriage of Klebsiella pneumoniae and Other Gram-Negative Bacilli in Pneumonia-Prone Age Groups in Semarang, Indonesia. *J Clin Microbiol* 2013;**51**:1614–6. doi:10.1128/JCM.00589-13

42 Pranavathiyani G, Prava J, Rajeev AC, *et al.* Novel Target Exploration from Hypothetical Proteins of Klebsiella pneumoniae MGH 78578 Reveals a Protein Involved in Host-Pathogen Interaction. *Front Cell Infect Microbiol* 2020;**10**:109. doi:10.3389/fcimb.2020.00109

43 Farida H, Severin JA, Gasem MH, *et al.* Nasopharyngeal Carriage of Streptococcus pneumonia in Pneumonia-Prone Age Groups in Semarang, Java Island, Indonesia. *PLoS One* 2014;**9**:e87431. doi:10.1371/journal.pone.0087431

44 Driscoll AJ, Deloria Knoll M, Hammitt LL, *et al.* The Effect of Antibiotic Exposure and Specimen Volume on the Detection of Bacterial Pathogens in Children With Pneumonia. *Clin Infect Dis* 2017;**64**:S368–77. doi:10.1093/cid/cix101

45 Andrade DC, Borges IC, Vilas-Boas AL, *et al.* Infection by Streptococcus pneumoniae in children with or without radiologically confirmed pneumonia. *J Pediatr (Rio J)* 2018;**94**:23–30. doi:10.1016/j.jped.2017.03.004

For peer review only

For peer review only - http://bmjopen.bmj.com/site/about/guidelines.xhtml

46 Principi N, Esposito S. Management of severe community-acquired pneumonia of children in developing and developed countries. *Thorax* 2011;**66**:815–22. doi:10.1136/thx.2010.142604

47 Lleo MM, Ghidini V, Tafi MC, *et al.* Detecting the presence of bacterial DNA by PCR can be useful in diagnosing culture-negative cases of infection, especially in patients with suspected infection and antibiotic therapy. *FEMS Microbiol Lett* 2014;**354**:153–60. doi:10.1111/1574-6968.12422

48 Torres A, Lee N, Cilloniz C, *et al.* Laboratory diagnosis of pneumonia in the molecular age. *Eur Respir J* 2016;**48**:1764 LP – 1778. doi:10.1183/13993003.01144-2016

49 Morozumi M, Chiba N, Ubukata K, *et al.* Antibiotic susceptibility in relation to genotype of Streptococcus pneumoniae, Haemophilus influenzae, and Mycoplasma pneumoniae responsible for community-acquired pneumonia in children. *J Infect Chemother* 2013;**19**:432–40. doi:10.1007/s10156-012-0500-x

50 Aydemir O, Aydemir Y, Ozdemir M. The role of multiplex PCR test in identification of bacterial pathogens in lower respiratory tract infections. *Pakistan J Med Sci* 2014;**30**:1011–6. doi:10.12669/pjms.305.5098

FIGURE CAPTIONS:

Figure 1. Subject screening, enrolment, and monitoring flowchart. CAP, community-acquired pneumonia; RR, respiratory rate; CXR, chest X-Ray; CRP, C-reactive protein; PCT, procalcitonin; NP, nasopharyngeal; IS, induced sputum; WB, whole blood; BC, blood culture; IS, induced sputum culture; PCR, polymerase chain reaction.

Figure 2. Pathogen Distribution. (A) Overall proportion of identified viral/bacterial/mixed pathogen, (B) Viral/bacterial/mixed pathogens by age group, (C) Pattern of detection of the ten most identified pathogens, (D) Distribution of ten most identified pathogens by age group. *$P<0.05$

Figure 3. Distribution of the (A) monthly count and (B) seasonal pattern of infection caused by *H. influenzae* non-type B, RSV, *K. pneumoniae*, *S. pneumoniae*, and Influenza virus during a 27-month study period.

SUPPLEMENTARY INFORMATION:

Details of Microbiological, Molecular and Serologic Methods, **Supplementary Table 1**

Antibiotic regimens administered prior to blood culture, **Supplementary Table 2**

Pathogen distribution by WHO severity classification status and mortality, **Supplementary Table 3**

For peer review only

Summary of fatal cases, **Supplementary Table 4.**

PEER-PePPeS Study sites, **Supplementary Figure 1**

Proportion of Identified Pathogen in each Sites, **Supplementary Figure 2**

Proportion of Identified Pathogen between WHO Severity Status, **Supplementary Figure 3**

For peer review only

For peer review only - http://bmjopen.bmj.com/site/about/guidelines.xhtml

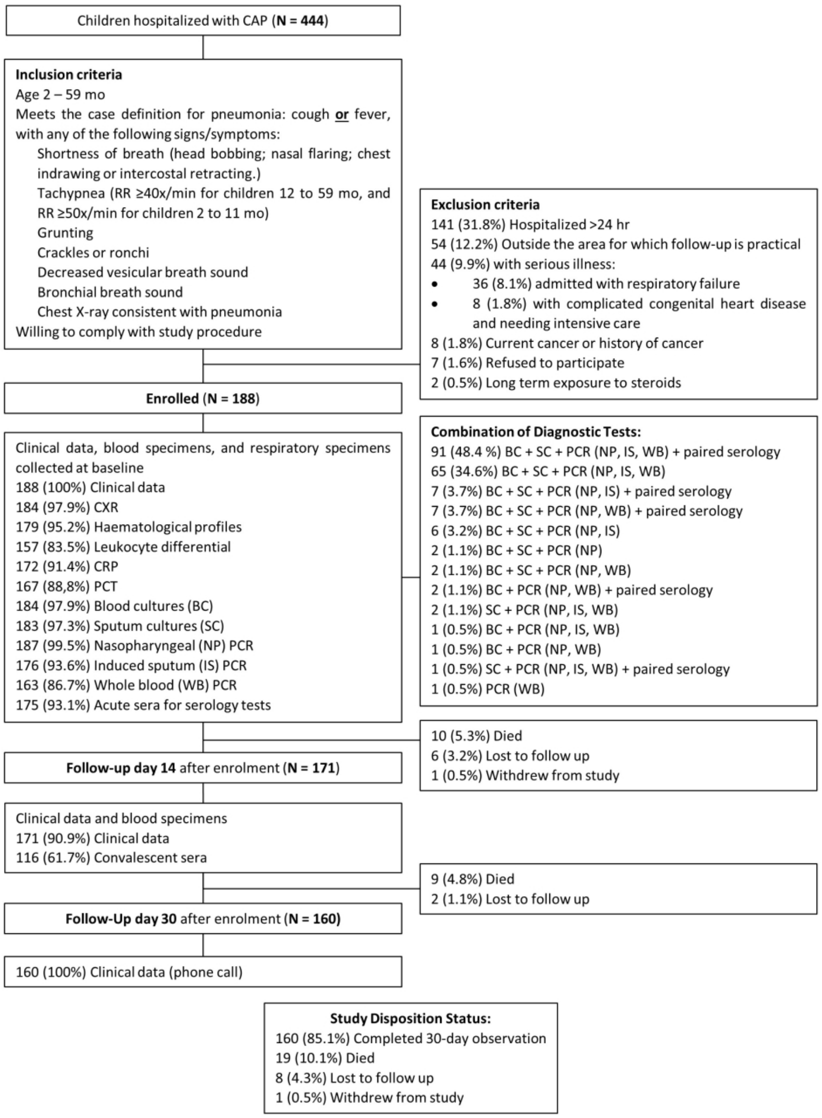

Figure 1. Subject screening, enrolment, and monitoring flowchart. CAP, community-acquired pneumonia; RR, respiratory rate; CXR, chest X-Ray; CRP, C-reactive protein; PCT, procalcitonin; NP, nasopharyngeal; IS, induced sputum; WB, whole blood; BC, blood culture; IS, induced sputum culture; PCR, polymerase chain reaction.

190x275mm (300 x 300 DPI)

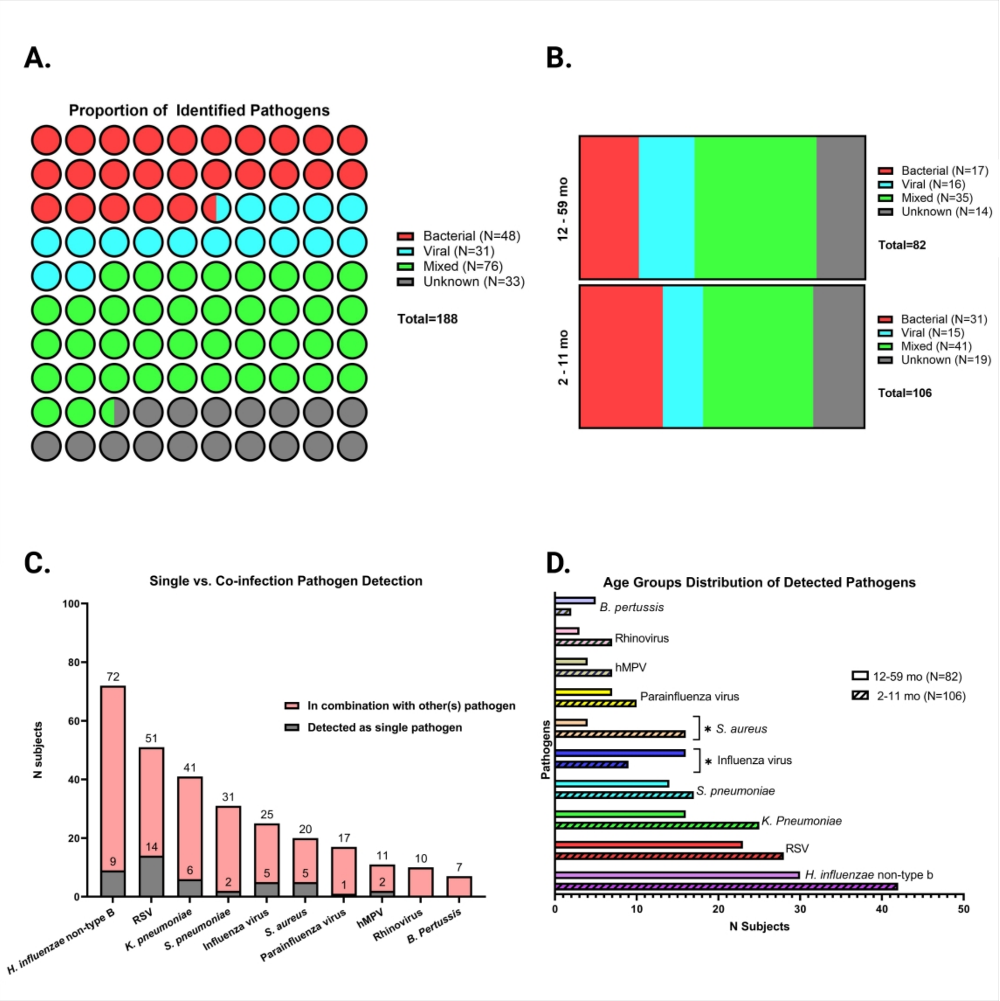

Figure 2. Pathogen Distribution. (A) Overall proportion of identified viral/bacterial/mixed pathogen, (B) Viral/bacterial/mixed pathogens by age group, (C) Pattern of detection of the ten most identified pathogens, (D) Distribution of ten most identified pathogens by age group. *P<0.05

381x381mm (300 x 300 DPI)

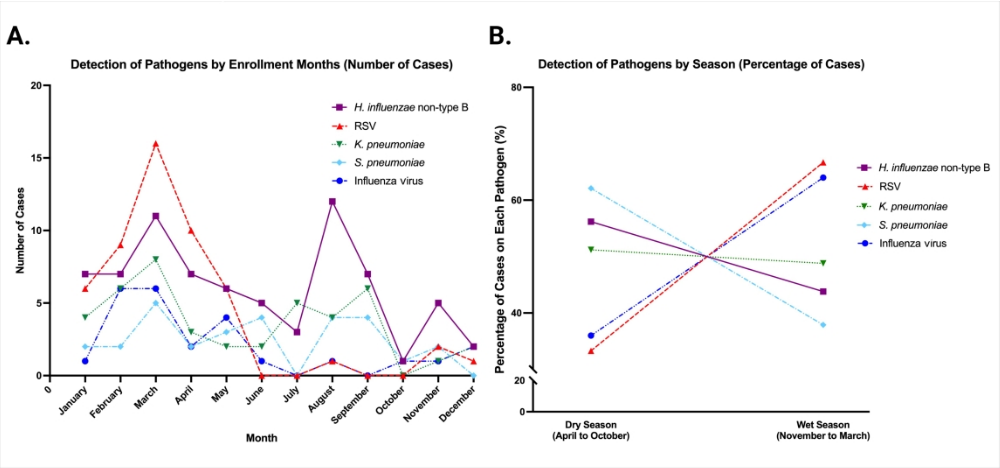

Figure 3. Distribution of the (A) monthly count and (B) seasonal pattern of infection caused by H. influenzae non-type B, RSV, K. pneumoniae, S. pneumoniae, and Influenza virus during a 27-month study period.

381x177mm (300 x 300 DPI)

SUPPLEMENTARY MATERIALS.

Supplementary Table 1. Microbiological, Molecular and Serologic Methods

No.	Assays	Procedures
1.	Gram stain	Gram-stained smears were obtained from the most purulent portion of each induced sputum specimen. The good quality specimen was defined as <10 squamous epithelium per low-power field (magnification, 100×) [1]. The procedure of the Gram stain required four basic steps that include applied a primary stain (crystal violet) to a heat-fixed smear, followed by the addition of a mordant (Gram's Iodine), rapid decolorization with alcohol, acetone, or a mixture of alcohol and acetone and lastly, counterstained with safranin [2]. The Gram-stained smears interpreted as follows: Gram-positive lancet-shaped diplococci (GPDC) suggest *Streptococcus pneumoniae*; Gram-positive diplococci (GPDC) or cocci in chains suggest *Streptococcus pyogenes*; Gram-positive cocci in clusters (GPC-cluster) suggest *Staphylococcus aureus*; Gram-negative coccobacilli (GNCB) suggest *Hemophilus influenzae*, *Bordetella pertussis* or *Acinetobacter baumannii*; Gram-negative diploccoci (GNDC) suggest *Moraxella catarrhalis*; large Gram-negative rods (GNR-large) suggest *Klebsiella pneumoniae* or *Escherichia coli*; and small Gram-negative rods (GNR-small) suggest *Pseudomonas aeruginosa* [3].
2.	Induced Sputum Culture	The most purulent portion of induced sputum was inoculated onto sheep blood, chocolate, and MacConkey agars, streaked out using a standard 4-quadrant streaking method, and incubated at 35°C for 48 hours. Cultures were examined at 24 hours and 48 hours, and predominant bacteria were identified and quantified according to the farthest quadrant with visible colonies (first quadrant, scanty; second quadrant, 1+; third quadrant, 2+; fourth quadrant, 3+) [4]. Then, the predominant bacteria isolates were inoculated into the appropriate VITEK identification strip using the VITEK® 2 COMPACT (BioMérieux, Germany). Briefly, a bacterial suspension was adjusted to a McFarland standard of 0.50 in a solution of 0.45 % sodium chloride using DensiLameter. The time between preparation of the solution and filling of the card was always less than 1 h. Analysis was done using the identification card and automatically read every 15 min. Bacteria identification and antibiotic susceptibility testing results were analyzed using the VITEK 2 software according to the manufacturer's instructions [5].
3.	Blood Culture	Up to 2 mL of blood samples (2 bottle sets) were collected and sent to the site laboratory with standardized procedures. Blood cultures were incubated for at least 5 days, unless positive, using automated systems (BacT/ALERT in Tangerang Hospital; BACTEC at other sites) [6]. Organisms were identified according to standard microbiological methods as described in induced sputum culture section. The following organisms were considered to be contaminants when identified in blood cultures: Coagulase-negative *staphylococci*, *Micrococcus* spp., *Propionibacterium* spp., Alpha-hemolytic streptococci (except

For peer review only

No.	Assays	Procedures
		pneumococcus, *Streptococcus anginosus*, and *Streptococcus mitis*), *Enterococcus* spp., *Corynebacterium* spp. (diphtheroids), *Bacillus* spp. (except *Bacillus anthracis*), *Pseudomonas* spp. (except *Pseudomonas aeruginosa*), *Stomatococcus, Aeroccocus, Neiserria subflava, Veillonella* spp., other environmental non-fermenting Gram negative rods, and *Candida* spp [7].
4.	Viral RNA Extraction	Viral RNA was extracted from viral transport media (VTM) containing respiratory swab as well as sputum, using the QIAamp Viral RNA Mini Kit (Qiagen, Hilden, Germany) according to the manufacturer's protocol. Briefly, 140 µl of VTM or sputum coat was lysed in 560 of carrier RNA-containing AVL buffer, followed by the binding of viral RNA to the QIAamp membrane. Contaminants were removed from viral RNA in two separate washing steps using two different wash buffers, AW1 and AW2. Viral RNA was eluted in 60 µl of AVE buffer and kept in -80° C if not directly used [8,9].
5.	Bacterial DNA Extraction	Bacterial DNA was extracted from viral transport media (VTM) containing respiratory swab as well as sputum, using the QIAamp® DNA Mini Kit (Qiagen, Hilden, Germany) according to the manufacturer's protocol. Briefly, 20 µl of QIAGEN Protease and 200 µl of VTM or sputum coat was lysed in 200 of AL buffer, followed binding of DNA to the QIAamp membrane. Contaminants were removed from DNA in two separate washing steps using two different wash buffers, AW1 and AW2. Bacterial DNA was eluted in 200 µl of AE buffer and kept in -80° C if not directly used
6.	qPCR for Respiratory Viruses	The realtime PCR for respiratory virus detection was done followed the protocol of Beld et al., 2004 and Jansen et al., 2011. Positive control is a synthetic plasmid carrying the nucleotide sequence of the detection target. Primers, probes, and positive controls were synthesized and purified by an outside vendor (Integrated DNA Technologies, Iowa, US). Realtime PCR was done using the TaqManTM Fast Virus 1-Step Master Mix (Thermo Fisher Scientific; Cat#: 4444432) in an Applied Biosystems 7500 Fast Realtime PCR System (Thermo Fisher Scientific, MA, US). The reaction mixture composition was 1X TaqManTM Fast Virus 1-Step Master Mix, 0.5 µM of each primer, 0.25 µM probe, and 4 µl RNA, in a total 20 µl volume. The cycle condition was 50° reverse transcription for 5 minutes, 95° C initial denaturation for 20 seconds, followed by 45 cycles of denaturation (95° C, 3 seconds) and annealing/elongation (55° C, 30 seconds). Realtime PCR works correctly when the positive control demonstrates the amplification curve and the template-free (negative) control demonstrates no amplification curve (no Ct values) [8,9].
7.	qPCR for Respiratory Bacteria	In real-time PCR (qPCR) a portion of bacterial DNA genome specific to the pathogen(s) of interest is amplified using a specific pair of primers and probes for each bacteria, that were selected from the available literature [10–14]. A detector (TaqMan® probe) is used in the reaction. Mastermix is prepared in a 1.5-ml tube for total reaction. qPCR assays were carried out in a total volume of 20 µL, comprising 10 µL of TaqMan® Fast Universal PCR Master Mix, 1.4 µL of nuclease-free water (Promega), 3.6 µL of oligonucleotide mixtures, and 4 µL of

No.	Assays	Procedures
		DNA extract. The cycle condition was 95° C initial denaturation for 20 seconds, followed by 45 cycles of denaturation (95° C, 3 seconds) and annealing/elongation (58° C, 30 seconds). Realtime PCR works correctly when the positive control demonstrates the amplification curve and the template-free (negative) control demonstrates no amplification curve (no Ct values)
8.	Serology Test	Assays were obtained from SERION ELISA classic kit (Institut Virion/Serion Laboratories, Germany) and used according to the insert of SERION kit. SERION ELISA classic is a qualitative and quantitative immunoassay for detecting human antibodies in serum or plasma with their corresponding antigen. The indirect enzyme immunosorbent assay in this kit was coated with specific antigens of the pathogen of interest. Patient sera are diluted in a rheumatoid factor and then diluted in Sample Diluent (containing phosphate with tween 20 and Bromphenol blue) and incubated in the coated microwells to bind serum antibody to the solid-phase antigen. The microwells are then washed to remove unreacted serum proteins, and enzyme conjugate (anti-human IgA, IgG, or IgM APC_Alkaline phosphatase) is added to label the bound antibody. After further incubation, the microwells are washed to remove unbound APC Conjugate. The pNPP (para-nitrophenyl phosphate) substrate is then added to quantitate the Conjugate-bound p-nitrophenyl phosphate portion. The colorless substrate p-nitrophenyl phosphate is then converted into the colored product p-nitrophenol. The signal intensity of this reaction product is proportional to the concentration of the analyte in the serum antibody. This timed reaction is interrupted with a Stop Solution (sodium hydroxide). Color intensity (Absorbance) is measured at a wavelength of 405nm on a microtiter plate reader or spectrophotometer within 15 minutes of adding the stop solution. Antibody activities are calculated by the SERION evaluation software [15].

Footnote References:

1. Murdoch DR, Morpeth SC, Hammitt LL, et al. Microscopic Analysis and Quality Assessment of Induced Sputum From Children With Pneumonia in the PERCH Study. *Clin Infect Dis an Off Publ Infect Dis Soc Am*. 2017;64(suppl_3):S271-S279. doi:10.1093/cid/cix083
2. Smith AC, Hussey MA. Gram stain protocols. *Am Soc Microbiol*. 2005;1:14.
3. Mardian Y, Menur Naysilla A, Lokida D, et al. Approach to Identifying Causative Pathogens of Community-Acquired Pneumonia in Children Using Culture, Molecular, and Serology Tests. *Front Pediatr*. 2021;9:498. doi:10.3389/fped.2021.629318
4. Murdoch DR, Morpeth SC, Hammitt LL, et al. The Diagnostic Utility of Induced Sputum Microscopy and Culture in Childhood Pneumonia. *Clin Infect Dis an Off Publ Infect Dis Soc Am*. 2017;64(suppl_3):S280-S288. doi:10.1093/cid/cix090
5. Książczyk M, Kuczkowski M, Dudek B, et al. Application of Routine Diagnostic Procedure, VITEK 2 Compact, MALDI-TOF MS, and PCR Assays in Identification Procedure of Bacterial Strain with Ambiguous Phenotype. *Curr Microbiol*. 2016;72(5):570-582. doi:10.1007/s00284-016-0993-0
6. Kirn TJ, Weinstein MP. Update on blood cultures: how to obtain, process, report, and interpret. *Clin Microbiol Infect Off Publ Eur Soc Clin Microbiol Infect Dis*. 2013;19(6):513-520. doi:10.1111/1469-0691.12180
7. O'Brien KL, Baggett HC, Brooks WA, et al. Causes of severe pneumonia requiring hospital admission in children without HIV infection from Africa and Asia: the PERCH multi-country case-control study. *Lancet*. 2019;394(10200):757-779. doi:10.1016/S0140-6736(19)30721-4
8. Beld M, Minnaar R, Weel J, et al. Highly sensitive assay for detection of enterovirus in clinical specimens by reverse transcription-PCR with an armored RNA internal control. *J Clin Microbiol*. 2004;42(7):3059-3064. doi:10.1128/JCM.42.7.3059-3064.2004
9. Jansen RR, Schinkel J, Koekkoek S, et al. Development and evaluation of a four-tube real time multiplex PCR assay covering fourteen respiratory viruses, and comparison to its corresponding single target counterparts. *J Clin Virol Off Publ Pan Am Soc Clin Virol*. 2011;51(3):179-185. doi:10.1016/j.jcv.2011.04.010

For peer review only - http://bmjopen.bmj.com/site/about/guidelines.xhtml

10. Reischl U, Lehn N, Sanden GN, Loeffelholz MJ. Real-time PCR assay targeting IS481 of Bordetella pertussis and molecular basis for detecting Bordetella holmesii. *J Clin Microbiol*. 2001;39(5):1963-1966. doi:10.1128/JCM.39.5.1963-1966.2001
11. Pitcher D, Chalker VJ, Sheppard C, George RC, Harrison TG. Real-time detection of Mycoplasma pneumoniae in respiratory samples with an internal processing control. *J Med Microbiol*. 2006;55(Pt 2):149-155. doi:10.1099/jmm.0.46281-0
12. Greiner O, Day PJR, Altwegg M, Nadal D. Quantitative detection of Moraxella catarrhalis in nasopharyngeal secretions by real-time PCR. *J Clin Microbiol*. 2003;41(4):1386-1390. doi:10.1128/JCM.41.4.1386-1390.2003
13. Meyler KL, Meehan M, Bennett D, Cunney R, Cafferkey M. Development of a diagnostic real-time polymerase chain reaction assay for the detection of invasive Haemophilus influenzae in clinical samples. *Diagn Microbiol Infect Dis*. 2012;74(4):356-362. doi:10.1016/j.diagmicrobio.2012.08.018
14. Gadsby NJ, McHugh MP, Russell CD, et al. Development of two real-time multiplex PCR assays for the detection and quantification of eight key bacterial pathogens in lower respiratory tract infections. *Clin Microbiol Infect Off Publ Eur Soc Clin Microbiol Infect Dis*. 2015;21(8):788.e1-788.e13. doi:10.1016/j.cmi.2015.05.004
15. Gasem MH, Kosasih H, Tjitra E, et al. An observational prospective cohort study of the epidemiology of hospitalized patients with acute febrile illness in Indonesia. *PLoS Negl Trop Dis*. 2020;14(1):e0007927. doi:10.1371/journal.pntd.0007927

Supplementary Table 2. Antibiotic regimens administered prior to blood culture

Antibiotic Regimen, (Dose)	All sites (N=188), Administered Dose(s) prior to blood culture, N (%)	Semarang (N=47) Administered Dose(s) prior to blood culture, N (%)	Yogyakarta (N=52) Administered Dose(s) prior to blood culture, N (%)	Tangerang (N=89) Administered Dose(s) prior to blood culture, N (%)
Ampicillin (50 mg/kg IV q6hr) + Gentamicin (2 – 7.5 mg/kg IV q24hr)	65 (34.6)<br>1x: 45 (24.0)<br>2x: 20 (10.6)	25 (53.2)<br>1x: 20 (42.6)<br>2x: 5 (10.6)	40 (76.9)<br>1x: 25 (48.1)<br>2x: 15 (28.8)	0 (0)
Cefotaxime (50 – 100 mg/kg IV q6hr)	32 (17.0)<br>All received 1 dose	0 (0)	0 (0)	32 (36.0)<br>All received 1 dose
Ceftriaxone (50 mg/kg IV q12hr)	27 (14.4)<br>All received 1 dose	0 (0)	0 (0)	27 (30.3)<br>All received 1 dose
Ampicillin (50 mg/kg IV q6hr)	14 (7.4)<br>1x: 10 (5.3)<br>2x: 4 (2.1)	5 (10.6)<br>All received 1 dose	9 (17.3)<br>1x: 5 (9.6)<br>2x: 4 (7.7)	0 (0)
Gentamicin (2 – 7.5 mg/kg IV q24hr)	3 (1.6)<br>1x: 2 (1.1)<br>2x: 1 (0.5)	3 (6.4)<br>1x: 2 (4.3)<br>2x: 1 (2.1)	0 (0)	0 (0)
Ceftazidime (50 – 100 mg/kg IV q8hr)	3 (1.6)<br>All received 1 dose	0 (0)	0 (0)	3 (3.4)<br>All received 1 dose
Cefamandole (50 – 100 mg/kg IV q12hr)	2 (1.1)<br>1x: 1 (0.5)<br>2x: 1 (0.5)	2 (4.3)<br>1x: 1 (2.1)<br>2x: 1 (2.1)	0 (0)	0 (0)
Ceftriaxone (50 mg/kg IV q12hr) + Gentamicin (2 – 7.5 mg/kg IV q24hr)	2 (1.1)<br>All received 1 dose	2 (4.3)<br>All received 1 dose	0 (0)	0 (0)
Amikacin (15 mg/kg IV q8hr) + Cefotaxime (50 – 100 mg/kg IV q6hr)	1 (0.5)<br>All received 1 dose	1 (2.1)<br>All received 1 dose	0 (0)	0 (0)
Amoxicillin syrup (40 mg/kg PO q12hr)	1 (0.5)<br>All received 1 dose	1 (2.1)<br>All received 1 dose	0 (0)	0 (0)

IV = intravenous; PO = peroral; qXhr = given at X hour intervals.

For peer review only

Supplementary Table 3. Pathogen distribution by WHO severity classification status and mortality.

Pathogens	WHO Classification System		*p-value*	Mortality Outcome		*p-value*
	Severe (N=89)	Non-severe (N=99)		Died (N=19)	Alive (N=169)	
**Causative Pathogen**						
H. influenzae non-type b	31 (34.8%)	42 (42.4%)	0.286	8 (42.1%)	65 (38.5%)	0.757
RSV	25 (28.1%)	26 (26.3%)	0.778	2 (10.5%)	49 (29.0%)	0.086
K. pneumoniae	15 (16.9%)	28 (28.3%)	0.062	6 (31.6%)	37 (21.9%)	0.388
S. pneumoniae	19 (21.3%)	10 (10.1%)	0.033	1 (5.2%)	28 (16.6%)	0.317
Influenza virus	9 (10.1%)	16 (16.2%)	0.223	3 (15.8%)	22 (13.0%)	0.723
S. aureus	8 (9.0%)	12 (12.1%)	0.487	0 (0.0%)	20 (11.8%)	0.230
PIV	8 (9.0%)	9 (9.1%)	0.981	1 (5.3%)	16 (9.5%)	1.000
hMPV	6 (6.7%)	5 (5.1%)	0.622	1 (5.3%)	10 (5.9%)	1.000
Rhinovirus	7 (7.9%)	3 (3.0%)	0.196	1 (5.3%)	9 (5.3%)	1.000
B. pertussis	4 (4.5%)	3 (3.0%)	0.709	2 (10.5%)	5 (3.0%)	0.150
**Infection Type**						
Bacterial pathogen	17 (19.1%)	31 (31.3%)	0.055	7 (36.8%)	41 (24.3%)	0.268
Viral pathogen	16 (18.0%)	15 (15.2%)	0.602	2 (10.5%)	29 (17.2%)	0.744
Mixed pathogen	38 (42.7%)	38 (38.4%)	0.547	5 (26.3%)	71 (42.0%)	0.186
Unknown pathogen	18 (20.2%)	15 (15.2%)	0.361	5 (26.3%)	28 (16.6%)	0.337

Differences in categorical variables were compared using Pearson χ2 or Fisher's exact test when the expected values in any of the contingency table cells were below 5.

Supplementary Table 4. Summary of fatal cases.

Case, Site, Gender (Age, mo)	Medical History	Signs and Symptoms (SS), Vital Signs (VS), Laboratory parameter (Lab) at admission	CXR	Causative Pathogen	ABX during Hospitalization	Hospitalization status	Cause of Death
#01, SMG, Male (4)	Recurrent pneumonia, congenital heart disease, severe malnutrition	• **SS:** Cough, fever, dyspnea, chest indrawing, intercostal retraction, rhonchi<br>• **VS:** 38°C, RR 44x/min, SpO_2 97%<br>• **Lab:** Hb 9.6 g/dL, WBC 24.1 $\times 10^9$/L, PLT 350 $\times 10^9$/L, NLR 4.63, CRP 25.70 mg/L, PCT 2.41 ng/mL	Alveolar infiltrate	Rhinovirus, *H. influenzae non-type b*	Ampicillin, Gentamicin, Ceftriaxon, Cefoperazone Sulbactam	On mechanical ventilator<br>ICU admission (25 days)<br>Died on Day-26	Cardiopulmonary failure<br>Sepsis
#02, SMG, Female (23)	Recurrent pneumonia, congenital heart disease, incomplete NIP (DPT-Hib), malnutrition, developmental delay	• **SS:** Cough, fever, dyspnea, chest indrawing, intercostal retraction, rhonchi<br>• **VS:** 37.5°C, RR 56x/min, SpO_2 95%<br>• **Lab:** Hb 10.6 g/dL, WBC 14.1 $\times 10^9$/L, PLT 405 $\times 10^9$/L, NLR 9.63, CRP 14.90 mg/L, PCT 0.37 ng/mL	Alveolar and interstitial infiltrates	Influenza A (H1N1)	Ampicillin, Gentamicin, Metronidazole, Ceftriaxon, Meropenem	On mechanical ventilator<br>ICU admission (9 days)<br>Died on Day 21	Cardiopulmonary failure
#03, SMG, Female (11)	Low birth weight, congenital heart disease, incomplete NIP (Measles), severe malnutrition, developmental delay	• **SS:** Cough, fever, dyspnea, diarrhea, nasal flaring, chest indrawing, intercostal retraction, rhonchi<br>• **VS:** 38.3°C, RR 45x/min, SpO_2 96%<br>• **Lab:** Hb 8.1 g/dL, WBC 15.9 $\times 10^9$/L, PLT 677 $\times 10^9$/L, NLR 1.87	Alveolar and interstitial infiltrates	Influenza A (H3N2), *B. pertussis, H. influenzae non-type b*, *K. pneumoniae*	Ampicillin, Gentamicin, Azithromycin	On nasal cannula<br>Died on day 19	Cardiopulmonary failure
#04, SMG, Male (45)	Recurrent pneumonia, frontometaphyseal dysplasia syndrome,	• **SS:** Cough, fever, dyspnea, nasal flaring, intercostal retraction, rhonchi, wheezing<br>• **VS:** 36.7°C, RR 40x/min, SpO_2 99%	Alveolar infiltrate	Unknown	Ampicillin, Gentamicin	On Nasal cannula<br>Died on day 2	Respiratory failure

For peer review only

Case, Site, Gender (Age, mo)	Medical History	Signs and Symptoms (SS), Vital Signs (VS), Laboratory parameter (Lab) at admission	CXR	Causative Pathogen	ABX during Hospitalization	Hospitalization status	Cause of Death
	epilepsy, developmental delay	• **Lab:** Hb 13.7 g/dL, WBC 11.3 $\times10^9$/L, PLT 277 $\times10^9$/L, NLR 0.98, CRP 0.10 mg/L, PCT 0.05 ng/mL					
#05, SMG, Male (5)	Premature birth, low birth weight, recurrent pneumonia, congenital heart disease, incomplete NIP (DPT-Hib)	• **SS:** Cough, dyspnea, nasal flaring, chest indrawing, intercostal retraction,<br>• **VS:** 36.8°C, RR 30x/min, SpO_2 98%<br>• **Lab:** Hb 10.9 g/dL, WBC 12.4 $\times10^9$/L, PLT 396 $\times10^9$/L, CRP 0.80 mg/L, PCT 128 ng/mL	Alveolar infiltrate	*K. pneumoniae*	Ampicillin, Gentamicin	On Simple mask<br>ICU admission (1 day)<br>Died on day 6	Cardiopulmonary failure
#06, SMG, Female (3)	Recurrent pneumonia, incomplete NIP (DPT-Hib), malnutrition	• **SS:** Cough, dyspnea, chest indrawing, intercostal retraction, rhonchi<br>• **VS:** 36.7°C, RR 42x/min, SpO_2 99%<br>• **Lab:** Hb 8.2 g/dL, WBC 16 $\times10^9$/L, PLT 499 $\times10^9$/L, ANC 6.7, NLR 0.76, CRP 13.10 mg/L, PCT 0.28 ng/mL	Alveolar infiltrate	Unknown	Ampicillin, Gentamicin, Vancomycin, Metronidazol, Meropenem	On mechanical ventilator<br>ICU admission (7 days)<br>Died on day 18	Septic shock, respiratory failure
#07, YGY, Female (10)	Congenital heart disease, incomplete NIP (DPT-Hib, and Measles), severe malnutrition, developmental delay	• **SS:** Cough, fever, dyspnea, head bobbing, chest indrawing, intercostal retraction, rhonchi<br>• **VS:** 39.0 °C, RR 64x/min, SpO_2 96%<br>• **Lab:** Hb 10.1 g/dL, WBC 12.1 $\times10^9$/L, PLT 415 $\times10^9$/L, ANC 6.0, NLR 1.15, CRP 4.90 mg/L, PCT 0.11 ng/mL	Alveolar infiltrate	hMPV, RSV A	Ampicillin, Gentamicin, Ceftriaxone, Cotrimoxazole	On mechanical ventilator/ ICU admission (13 days)<br>Died on day 17	Sepsis, Pulmonary crisis due to pulmonary hypertension
#08, YGY, Female (3)	Low birth weight, congenital heart disease, incomplete NIP (DPT-Hib), severe malnutrition	• **SS:** Cough, fever, dyspnea, chest indrawing, intercostal retraction, rhonchi<br>• **VS:** 37.2 °C, RR 49x/min, SpO_2 56%<br>• **Lab:** Hb 9.7 g/dL, WBC 11.3 $\times10^9$/L, PLT 115 $\times10^9$/L, ANC 7.0, NLR 1.92	Alveolar and interstitial infiltrates	Unknown	Ampicillin, Ceftriaxone	On nasal cannula<br>Hospital discharge on day 10<br>Died on day 29 (outside hospitalization)	Acute Respiratory Distress Syndrome
#09, YGY, Female (5)	Congenital heart disease, incomplete NIP (DPT-Hib), severe malnutrition	• **SS:** Cough, dyspnea, inability to drink, nasal flaring, chest indrawing, intercostal retraction, rhonchi<br>• **VS:** 37.0 °C, RR 60x/min, SpO_2 96%<br>• **Lab:** Hb 10.3 g/dL, WBC 26.9 $\times10^9$/L, PLT 788 $\times10^9$/L, ANC 18.5, NLR 2.97	Alveolar and interstitial infiltrates	*H. influenzae non-type b, K. pneumoniae*	Ampicillin, Gentamicin	On nasal cannula<br>Died on day 15	Aspiration, mucous hypersecretion
#10, YGY, Male (6)	Recurrent pneumonia, congenital heart disease, tuberculosis, incomplete NIP (DPT-Hib)	• **SS:** Cough, fever, dyspnea, nasal flaring, chest indrawing, intercostal retraction, rhonchi, wheezing<br>• **VS:** 37.3 °C, RR 50x/min, SpO_2 89%<br>• **Lab:** Hb 11.6 g/dL, WBC 13.3 $\times10^9$/L, PLT 189 $\times10^9$/L, ANC 3.7, NLR 0.48, CRP 4.90 mg/L, PCT 0.08 ng/mL	Alveolar and interstitial infiltrates, pleural effusion	*K. pneumoniae*	Ampicillin, Gentamicin, Ceftriaxone	On non-rebreather mask<br>Died on day 4	Septic shock
#11, TRG, Female (5)	Premature birth, developmental delay	• **SS:** Cough, fever, dyspnea, nasal flaring, rhonchi, wheezing<br>• **VS:** 37.5 °C, RR 48x/min, SpO_2 31%<br>• **Lab:** Hb 8.5 g/dL, WBC 12.1 $\times10^9$/L, PLT 208 $\times10^9$/L, ANC 8.6, NLR 3.23, CRP 0.91 mg/L, PCT 0.74 ng/mL	Alveolar infiltrate	*A. baumannii* (MDR)	Cefotaxime	On Nasal cannula<br>Hospital discharge on day 7<br>Died on day 17 (outside hospitalization)	Unknown death
#12, TRG, Female (2)	Incomplete NIP (DPT-Hib)	• **SS:** Cough, fever, dyspnea, diarrhea, skin rash, intercostal retraction, rhonchi, wheezing<br>• **VS:** 37.6 °C, RR 63x/min, SpO_2 93%<br>• **Lab:** Hb 10.5 g/dL, WBC 13.6 $\times10^9$/L, PLT 289 $\times10^9$/L, ANC 10.2, NLR 3.95, CRP 175.30 mg/L, PCT 0.7 ng/mL	Alveolar and interstitial infiltrates	Unknown	Ceftriaxone, Ceftazidime, Azithromycin	On Nasal cannula<br>Died on day 8	Sepsis
#13, TRG, Female (2)	Incomplete NIP (DPT-Hib)	• **SS:** Cough, fever, dyspnea, nasal flaring, chest indrawing, intercostal retraction, rhonchi<br>• **VS:** 36 °C, RR 45x/min, SpO_2 96%<br>• **Lab:** Hb 7.8 g/dL, WBC 21.2 $\times10^9$/L, PLT 563 $\times10^9$/L, ANC 16.5, NLR 3.9, CRP 280.30 mg/L, PCT 0.09 ng/mL	Alveolar and interstitial infiltrates, pleural effusion	Influenza B, *S. mitis* (MDR)	Ceftazidime	On non-rebreather mask ICU admission (3 days)<br>Died on day 3	Respiratory Failure

For peer review only

Case, Site, Gender (Age, mo)	Medical History	Signs and Symptoms (SS), Vital Signs (VS), Laboratory parameter (Lab) at admission	CXR	Causative Pathogen	ABX during Hospitalization	Hospitalization status	Cause of Death
#14, TRG, Female (2)	Congenital heart disease, incomplete NIP (DPT-Hib), severe malnutrition	• **SS:** Cough, fever, dyspnea, nasal flaring, chest indrawing, intercostal retraction, rhonchi, wheezing<br>• **VS:** 37 ^{o}C, RR 60x/min, SpO_2 76%<br>• **Lab:** Hb 9.5 g/dL, WBC 17.2 $\times10^9$/L, PLT 296 $\times10^9$/L, ANC 8.8, NLR 1.42, CRP 0.70 mg/L, PCT 0.02 ng/mL	Interstitial infiltrate	Unknown	Cefotaxime	On Simple mask<br>Died on day 2	Respiratory Failure
#15, TRG, Male (9)	Incomplete NIP (Measles)	• **SS:** Cough, fever, dyspnea, nasal flaring, chest indrawing, intercostal retraction, rhonchi<br>• **VS:** 37 ^{o}C, RR 30x/min, SpO_2 89%<br>• **Lab:** Hb 6.4 g/dL, WBC 25.7 $\times10^9$/L, PLT 801 $\times10^9$/L, ANC 18.5, NLR 3.43, CRP 33.35 mg/L, PCT 0.34 ng/mL	Interstitial infiltrate	*H. influenzae non-type b*	Cefotaxime, Ceftriaxone, Meropenem	On mechanical ventilator<br>ICU admission (8 days)<br>Died on day 12	Meningoencephalitis, Respiratory Failure
#16, TRG, Female (4)	Premature birth, low birth weight, congenital heart disease, incomplete NIP (DPT-Hib)	• **SS:** Cough, fever, dyspnea, diarrhea, chest indrawing, intercostal retraction, rhonchi<br>• **VS:** 38 ^{o}C, RR 32x/min, SpO_2 85%<br>• **Lab:** Hb 9.2 g/dL, WBC 16.8 $\times10^9$/L, PLT 224 $\times10^9$/L, ANC 9.4, NLR 2.24, CRP 2.46 mg/L, PCT 2.24 ng/mL	Alveolar and interstitial infiltrates,	*H. influenzae non-type b, K. pneumoniae*	Cefotaxime, Gentamicin, Ceftriaxone	On nasal cannula<br>Died on day 11	Unknown death
#17, TRG, Female (20)	Developmental delay, incomplete NIP (DPT-Hib)	• **SS:** Cough, fever, dyspnea, chest indrawing, intercostal retraction, rhonchi<br>• **VS:** 36.3^{o}C, RR 40x/min, SpO_2 75%<br>• **Lab:** Hb 7.0 g/dL, WBC 15.2 $\times10^9$/L, PLT 668 $\times10^9$/L, ANC 9.7, NLR 2.13, CRP 55.10 mg/L	Alveolar and interstitial infiltrates, pleural effusion	*H. influenzae non-type b, K. pneumoniae*	Cefotaxime, Gentamicin, Ceftriaxone	On mechanical ventilator<br>ICU admission (3 days)<br>Died on day 8	Septic shock, Cardiopulmonary failure
#18, TRG, Male (4)	Low birth weight, developmental delay, recurrent pneumonia, incomplete NIP (DPT-Hib), severe malnutrition	• **SS:** Cough, fever, dyspnea, nasal flaring, chest indrawing, intercostal retraction, rhonchi<br>• **VS:** 36.7 ^{o}C, RR 30x/min, SpO_2 92%<br>• **Lab:** Hb 11.6 g/dL, WBC 20.5 $\times10^9$/L, PLT 433 $\times10^9$/L, ANC 11.9, NLR 2.52, CRP 16.80 mg/L, PCT 20.1 ng/mL	Alveolar and interstitial infiltrates,	PIV 3, *H. influenzae non-type b, S. pneumoniae*	Ceftazidime	On non-rebreather mask<br>Died on day 3	Respiratory failure
#19, TRG, Male (15)	Incomplete NIP (DPT-Hib and Measles)	• **SS:** Cough, fever, dyspnea, rhonchi<br>• **VS:** 37.8 ^{o}C, RR 52x/min, SpO_2 80%<br>• **Lab:** Hb 9.4 g/dL, WBC 23.6 $\times10^9$/L, PLT 786 $\times10^9$/L, CRP 3.30 mg/L, PCT 0.07 ng/mL	Interstitial infiltrate	RSV B, *B. pertussis, H. influenzae non-type b*	Cefotaxime	On nasal cannula<br>Hospital discharged on day 5<br>Died on day 20 (outside hospitalization)	Unknown death

Abbreviation. SMG: Semarang site; YGY: Yogyakarta site; TGR: Tangerang site; NIP: mandatory National Immunization Program; DPT-Hib: a combined vaccine of adsorbed diphtheria, tetanus toxoids, acellular pertussis and of *Haemophilus influenzae* type b conjugate vaccines; CXR: chest X-ray; ABX: Antibiotics; RSV: Respiratory Syncytial Virus; hMPV: Human Metapneumovirus; PIV: Parainfluenza Virus; MDR: Multiple drug resistance.

For peer review only

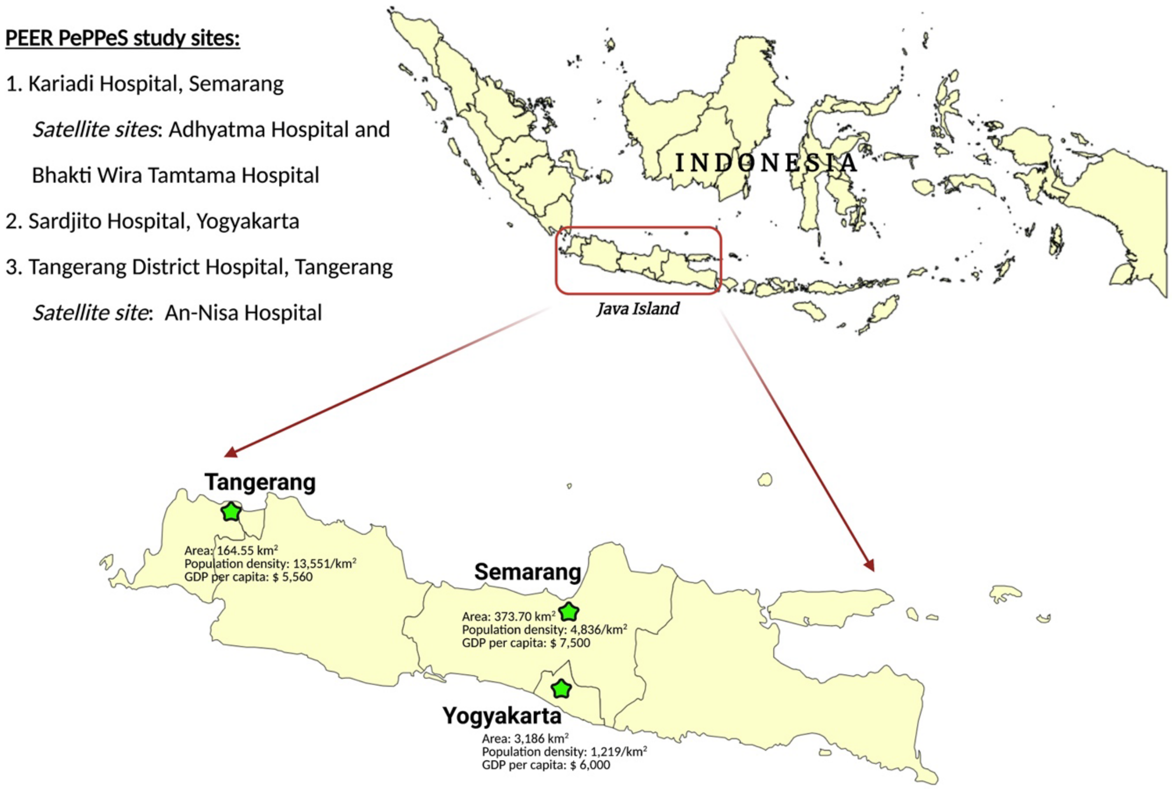

Supplementary Figure 1. PEER-PePPeS Study sites

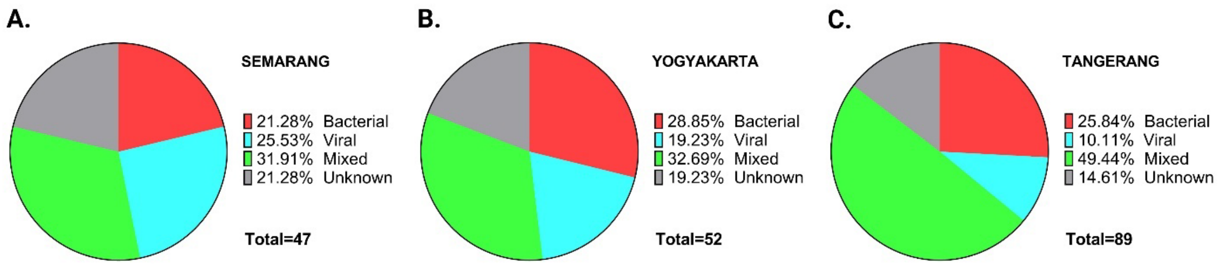

Supplementary Figure 2. Proportion of Identified Pathogen in each Sites. (A) Semarang, (B) Yogyakarta, and (C) Tangerang

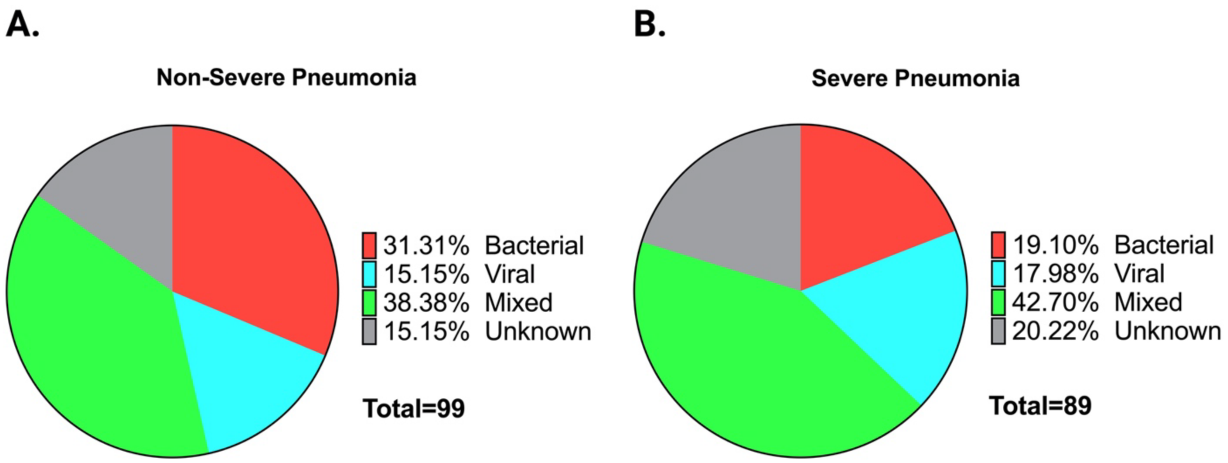

Supplementary Figure 3. Proportion of Identified Pathogen between WHO Severity Status. (A) Non-severe Pneumonia, (B) Severe Pneumonia.

STROBE Statement—Checklist of items that should be included in reports of ***cohort studies***

	Paragraph/ Line number	Recommendation
**Title and abstract**	Page 1-2	(*a*) Indicate the study's design with a commonly used term in the title or the abstract
		(*b*) Provide in the abstract an informative and balanced summary of what was done and what was found
**Introduction**		
Background/rationale	Introduction, paragraph 1-3 (Page 3-4)	Explain the scientific background and rationale for the investigation being reported
Objectives	Introduction, paragraph 4 (Page 4)	State specific objectives, including any prespecified hypotheses
**Methods**		
Study design	Methods, paragraph 1 (Page 4)	Present key elements of study design early in the paper
Setting	Methods, paragraph 1 and 4 (Page 4-5)	Describe the setting, locations, and relevant dates, including periods of recruitment, exposure, follow-up, and data collection
Participants	Methods, paragraph 4 (Page 5)	(*a*) Give the eligibility criteria, and the sources and methods of selection of participants
Variables	Methods, paragraph 2, 3, and 5 (Page 4-5)	Clearly define all outcomes, exposures, predictors, potential confounders, and effect modifiers. Give diagnostic criteria, if applicable
Data sources/ measurement	Methods, paragraph 5, 6, 7, and 8 (Page 5-6)	For each variable of interest, give sources of data and details of methods of assessment (measurement). Describe comparability of assessment methods if there is more than one group
Bias	Methods, paragraph 9 and 10 (Page 6-7)	Describe any efforts to address potential sources of bias
Study size	Methods, paragraph 1 and 4 (Page 4-5)	Explain how the study size was arrived at
Quantitative variables	Methods, paragraph 9 (Page 6)	Explain how quantitative variables were handled in the analyses. If applicable, describe which groupings were chosen and why
Statistical methods	Methods, paragraph 9 (Page 6)	(*a*) Describe all statistical methods, including those used to control for confounding
		(*b*) Describe any methods used to examine subgroups and interactions
		(*c*) Explain how missing data were addressed
		(*d*) If applicable, describe analytical methods taking account of sampling strategy
		(*e*) Describe any sensitivity analyses
**Results**		
Participants	Results, paragraph 1 (Page 7) and Flow diagram/Fig. 1	(a) Report numbers of individuals at each stage of study—eg numbers potentially eligible, examined for eligibility, confirmed eligible, included in the study, completing follow-up, and analysed
		(b) Give reasons for non-participation at each stage
		(c) Consider use of a flow diagram
Descriptive data	Results, paragraph 2 and 3. Table 1. (Page 7-9)	(a) Give characteristics of study participants (eg demographic, clinical, social) and information on exposures and potential confounders
		(b) Indicate number of participants with missing data for each variable of interest
Outcome data	Results, paragraph 4 and 5 (Page 9-10)	Report numbers of outcome events or summary measures
Main results	Results, paragraph 5, 7, and 9. Table 2. (Page 10-	(*a*) Give unadjusted estimates and, if applicable, confounder-adjusted estimates and their precision (eg, 95% confidence

For peer review only - http://bmjopen.bmj.com/site/about/guidelines.xhtml

	12). Fig. 2 and Fig. 3	interval). Make clear which confounders were adjusted for and why they were included
		(*b*) Report category boundaries when continuous variables were categorized
		(*c*) If relevant, consider translating estimates of relative risk into absolute risk for a meaningful time period
Other analyses	Results, paragraph 6 (Page 10). Results, paragraph 8. (Page 11-12).	Report other analyses done—eg analyses of subgroups and interactions, and sensitivity analyses
**Discussion**		
Key results	Discussion, paragraph 1 (Page 12)	Summarise key results with reference to study objectives
Limitations	Discussion, paragraph 11 (Page 15)	Discuss limitations of the study, taking into account sources of potential bias or imprecision. Discuss both direction and magnitude of any potential bias
Interpretation	Discussion, paragraph 2-10 (Page 12-15)	Give a cautious overall interpretation of results considering objectives, limitations, multiplicity of analyses, results from similar studies, and other relevant evidence
Generalisability	Discussion, paragraph 3-8 (Page 13-14) Discussion, paragraph 12 (Page 15)	Discuss the generalisability (external validity) of the study results
**Other information**		
Funding	Funding statement (Page 16)	Give the source of funding and the role of the funders for the present study and, if applicable, for the original study on which the present article is based

*Give information separately for exposed and unexposed groups.

Note: An Explanation and Elaboration article discusses each checklist item and gives methodological background and published examples of transparent reporting. The STROBE checklist is best used in conjunction with this article (freely available on the Web sites of PLoS Medicine at http://www.plosmedicine.org/, Annals of Internal Medicine at http://www.annals.org/, and Epidemiology at http://www.epidem.com/). Information on the STROBE Initiative is available at www.strobe-statement.org.

For peer review only

For peer review only - http://bmjopen.bmj.com/site/about/guidelines.xhtml
